# Ultrasound innovations in diaphragm assessment: an integrative review of expanding clinical applications

**DOI:** 10.1183/16000617.0089-2025

**Published:** 2025-10-08

**Authors:** Ivo Neto Silva, Claire Bennett, José Alberto Duarte, Karim Bendjelid

**Affiliations:** 1 Intensive Care Division, Department of Acute Medicine, Geneva University Hospitals, Geneva, Switzerland; 2 Geneva Hemodynamic Research Group, Faculty of Medicine, University of Geneva, Geneva, Switzerland; 3 Care Directorate, Geneva University Hospitals, Geneva, Switzerland; 4 Centre of Physical Activity, Health and Leisure (CIAFEL), Faculty of Sport, University of Porto, Porto, Portugal; 5 Division of Respiratory Medicine, Medicine Department, Geneva University Hospitals, Geneva, Switzerland; 6 TOXRUN-Toxicology Research Unit, University Institute of Health Sciences - CESPU, Gandra, Portugal

## Abstract

**Introduction:**

Diaphragm dysfunction is prevalent across various patient populations, requiring precise structural and functional assessment. Ultrasound, being bedside-accessible and radiation-free, has gained relevance for evaluating the diaphragm and other respiratory muscle. Recent advancements have introduced novel techniques that have expanding its assessment scope. This review aims to identify emerging ultrasound methods for quantitative diaphragm assessment in adults, emphasising reliability and clinical relevance.

**Methods:**

A systematic literature search was conducted using keywords related to the diaphragm, ultrasound techniques and innovation. We included original studies on adult participants using innovative ultrasound methods extending beyond conventional assessments. Studies lacking original data, case reports, animal studies and studies on automated analysis techniques were excluded. Screening and data extraction followed a structured process, with one researcher extracting data and a second verifying accuracy. Results were categorised by reliability and by physiological and clinical outcomes.

**Results:**

Of 1411 records screened, 288 full-text articles were reviewed, and 36 studies met inclusion criteria, with four additional studies identified *via* reference analysis. These studies, published between 2013 and 2024, explored seven innovative techniques: the area method, contrast-enhanced ultrasound, echogenicity/echodensity, excursion of the zone of apposition, shear wave/strain elastography, speckle tracking and pulsed-wave tissue Doppler imaging. Studies focused on both healthy subjects and critically ill, surgical and COPD patients.

**Conclusions:**

Recent ultrasound advancements enhance diaphragm assessment by evaluating muscle quality, functional mechanical properties and blood flow. These innovative methods also provide alternatives when conventional approaches are limited. Further research is essential to refine protocols, validate clinical applications and standardise assessments for broader implementation.

## Shareable abstract

**Emerging ultrasound techniques allow for a multidimensional approach to diaphragm assessment. This review explores their potential value in complementing conventional methods and their applicability regarding reliability and clinical outcomes.** https://bit.ly/4m8JWJJ

## Introduction

The diaphragm is a dome-shaped muscular structure and the primary inspiratory muscle [1, 2]. With its strategic anatomical location, the diaphragm plays a vital role beyond respiration, influencing posture [3–6], continence [7–9] and other physiological functions. Diaphragm dysfunction is common in critically ill patients [10–13], patients with COPD [14–18] and patients with neuromuscular disorders [19–22], significantly affecting clinical outcomes.

The respiratory muscle testing guidelines from the European Respiratory Society allow us to appreciate the advances made over the past 20 years and the existing multidimensional approach to assessing diaphragm structure and function. These guidelines also outline a wide range of assessment methods, including the use of ultrasound/ultrasonography [23]. Ultrasound is a highly useful tool, offering bedside accessibility, a radiation-free approach and broad availability in clinical settings. Key consensus documents [24–30] primarily focus on three established diaphragm ultrasound (DUS) markers: excursion (EXdi), thickness (Tdi) and thickening fraction (TFdi), which assess motion, atrophy and contractility. While highly valid, reproducible and rapidly applicable, these markers have technical and practical limitations.

Several new ultrasound-based techniques have emerged in recent years. These innovative methods not only serve as alternatives to assessing these same dimensions, such as motion, but also provide new cutting-edge perspectives, including diaphragm biomechanics. This evolution in techniques parallels our growing understanding of the mechanisms underlying diaphragmatic dysfunction, such as ventilator-induced myotrauma [31]. These approaches enhance the depth of an evaluation and provide valuable alternatives when standard methods are limited or challenging to implement.

The present article reviews emerging ultrasound techniques for the quantitative assessment of diaphragm structure and function in adult subjects. We also aimed to describe their role and applicability in terms of reliability and physiological and clinical outcomes. This analysis seeks to extend beyond conventional methods frequently cited in the literature, such as EXdi, Tdi and TFdi.

## Methods

### Eligibility criteria

Our inclusion criteria were 1) original studies with adult participants (≥18 years old), 2) the use of innovative ultrasound techniques that extend beyond conventional diaphragm assessments (*e.g.* EXdi, Tdi and TFdi) and 3) results from quantitative assessment of diaphragm structure and function using ultrasound.

We excluded studies lacking original data, case reports, conference abstracts and animal or *in vitro* studies. Only studies in English or French (the research team language) were considered. Additionally, studies focusing on automated analysis, machine learning and deep learning [32] were excluded because these methods require complex post-processing that diverge from our emphasis on direct or quasi-direct ultrasound techniques. We also excluded studies using ultrasound sensors for waveform acquisition, because they do not provide visualisation of diaphragm structure and function.

Of note, our definition of conventional DUS techniques includes exploratory markers derived from these methods, as also noted in the literature (*e.g.* thickening ratio instead of TFdi, inspiratory slope from M-mode EXdi, or EXdi measured at a low intercostal lateral probe position).

### Information sources

We conducted several computerised searches in the PubMed database, which includes MEDLINE (1946 to present), PubMed Central and National Center of Biotechnology Information Bookshelf, without date restrictions. The final search was performed on 14 January 2025. Additionally, we examined the reference lists of included studies and those that cited them through backwards–forwards citation searching using a specialised application (https://estech.shinyapps.io/citationchaser/) [33], conducted on 12 January 2025.

### Search strategy

Our search strategy focused on three concepts: 1) diaphragm muscle as a structure of interest; 2) ultrasound-based assessment method, including related terms that may replace “ultrasound” in titles and abstracts (*e.g.* elastography); and 3) innovative ultrasound techniques, categorised by dimensions such as “novelty”, “ultrasound technical features”, “blood flow” and “mechanical properties” (supplementary material 1).

### Selection process

All the records identified through our search strategy were transferred to the Rayyan web application [34] (Qatar Computing Research Institute, Doha, Qatar) for screening. Initially, one researcher (I. Neto Silva) screened the titles and abstracts based on the established inclusion and exclusion criteria. Records identified as related to DUS were carried forward to the next phase. A full-text screening was then conducted by the same researcher (I. Neto Silva), and in cases of uncertainty, a senior researcher (K. Bendjelid) was consulted.

### Data collection process

From each eligible record, we extracted the following data: first author's name, year of publication, country, study design, population characteristics, type of innovative ultrasound technique and its approach, comparators used and main results. The results were categorised into three sections: reliability data, physiological outcomes (*i.e.* other surrogate measures of diaphragm or respiratory function) and clinical outcomes related to the new ultrasound technique (supplementary material 2). Data extraction was conducted by one researcher (I. Neto Silva) and verified by a second researcher (C. Bennett) for accuracy and consistency.

## Results

### Study selection

Our search retrieved 1411 records, from which we reviewed 288 full-text documents. Of these, 36 papers met the inclusion criteria [35–70]. Additionally, we examined the reference lists of the included studies, resulting in the inclusion of four more papers from a total of 1008 cited/citing references [71–74].

**FIGURE 1:**
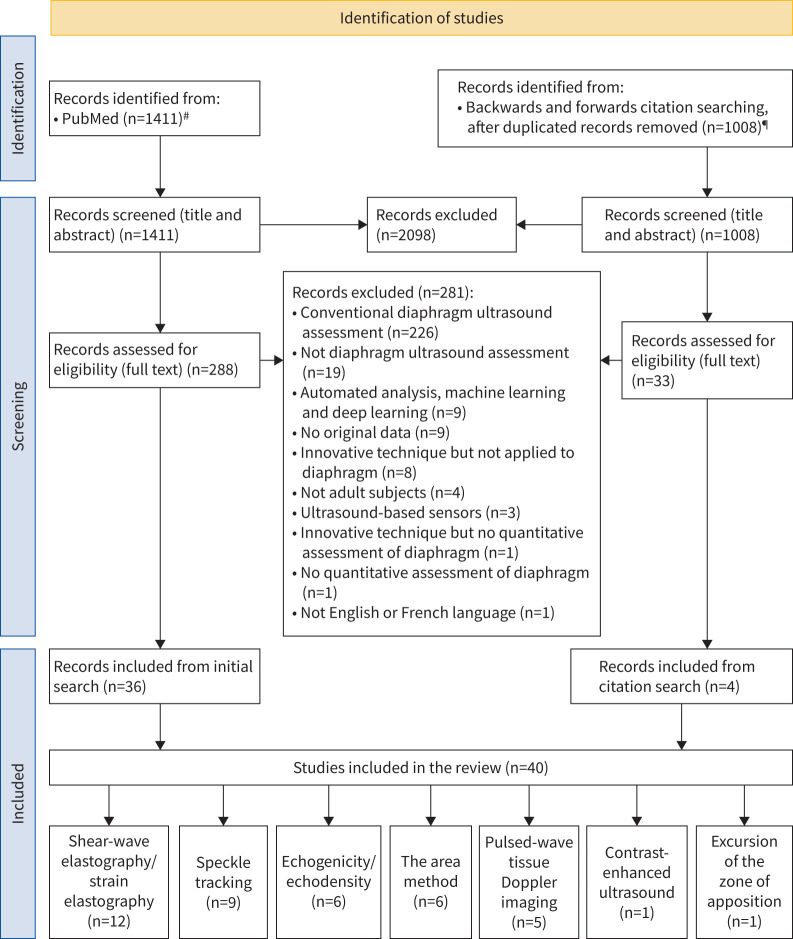
Flow diagram for study identification and selection, with reasons for exclusion. ^#^: last search on 14 January 2025; ^¶^: last search on 21 January 2025.

The primary reason for exclusion was the use of only conventional DUS assessments (n=226). Other main reasons included not being a DUS assessment (n=19), absence of original data (n=9) and the use of an innovative technique not applied to the diaphragm (n=8). Additionally, some excluded records employed innovative techniques but focused on automated analysis, machine learning and/or deep learning (n=9) [75–83]. A summary of the study selection process is presented in figure 1.

### Study characteristics

From the 40 included studies, we identified seven innovative ultrasound techniques for diaphragm assessment: the area method [35–37, 71–73], contrast-enhanced ultrasound (CEUS) [38], echogenicity/echodensity [39–44], excursion of the zone of apposition (EXdi-ZOA) [45], shear wave elastography (SWE)/strain elastography (SE) [46–56, 74], speckle tracking (ST) [57–65] and pulsed-wave tissue Doppler imaging (PW-TDI) [66–70]. These techniques were applied in studies published between 2013 and 2024, reflecting the recent and dynamic development of the field. The majority of studies were observational, with only two exceptions. Four studies employed an experimental design involving stepwise loading protocols in healthy participants. In terms of ultrasound protocols, only eight studies assessed both hemidiaphragms; the remainder focused solely on the right side. Characteristics of included studies are presented in table 1, including technical approaches. A full interactive version of table 1 is available online (Tableau Public) and can be accessed directly *via* the following link: https://public.tableau.com/app/profile/ivo.neto.silva/viz/UltrasoundInnovationsinDiaphragm-Table1/MapStudiesperCountry. Detailed information on ultrasound protocols is provided in supplementary material 2.

**TABLE 1:** Characteristics of the included studies (participants, study design and ultrasound assessment)

Innovative ultrasound technique	Publication, country	Population studied (n)	Study design	Assessed hemidiaphragm, probe type and position	Using the built-in measurement function of the ultrasound machine?	Diaphragmatic ultrasound markers
**Area method**	Skaarup 2018, Denmark [35]	Healthy adult volunteers (n=19)	Cohort study, single centre	Both left and right sides, 3–5 MHz curved probe, intercostal approach: mid-axillary line	Yes	Area change (cm^2^) (diaphragm movement in two dimensions: cranio-caudal and posterior-anterior)
	Skaarup 2020, Italy [71]	Patients with unilateral pleural effusion needing thoracentesis (n=32)	Prospective observational study, single centre	Both left and right sides, curved probe, intercostal approach: mid-axillary line	Yes	Area change (cm^2^) (diaphragm movement in two dimensions: cranio-caudal and posterior-anterior)
	Fjaellegaard 2024, Denmark [72]	Patients with unilateral pleural effusion needing thoracentesis (n=104)	Prospective observational study, single centre	Both left and right sides, 2–5 MHz curved abdominal probe (C1-5) or a 4–8 MHz micro-convex probe (C42), intercostal approach: mid-axillary line	Yes	Area change (cm^2^) (diaphragm movement in two dimensions: cranio-caudal and posterior-anterior)
	Nørskov 2024, Denmark [73]	Patients undergoing oesophageal resection or pulmonary lobectomy (n=40)	Prospective observational study, single centre	Both left and right sides, cardiac sector probe (phased array, M5Sc-D), intercostal approach: mid-axillary line	Yes	Area change (cm^2^) (diaphragm movement in two dimensions: cranio-caudal and posterior-anterior)
	Petersen 2024, Denmark [36]	Patients with pleural effusion needing thoracentesis (n=49)	Prospective observational study, single centre	Both left and right sides, 2–5 MHz curved (C1-6-D) probe, intercostal approach: mid-axillary line	Yes	Area change (cm^2^) (diaphragm movement in two dimensions: cranio-caudal and posterior-anterior)
	Skaarup 2024, Denmark [37]	Patients with COPD or interstitial lung disease, or post-thoracic surgery (heart transplantation or LVAD implant) or post-COVID-19 infection, healthy volunteers (n=42)	Prospective observational study, single centre	Both left and right sides, 3–5 MHz curved probe, intercostal approach: mid-axillary line	Yes	Area change (cm^2^) (diaphragm movement in two dimensions: cranio-caudal and posterior-anterior)
**Contrast-enhanced ultrasound**	Bird 2024, Canada [38]	Healthy adult volunteers (n=16)	Observational study (experimental design), single centre	Right side, linear probe (GE HealthCare 9L-D), intercostal approach (not specified)	No, publicly available software (https://www.narnarhealth.com/)	Microvascular blood volume of the diaphragm (AU), microvascular blood flux of the diaphragm (s^−1^), diaphragm blood flow (AU·s^−1^) and vascular conductance of the diaphragm (AU·s^−1^·mmHg^−1^)
**Echogenicity/echodensity**	Sarwal 2015, Australia and USA [39]	Critically ill patients (n=20)	Cross-sectional observational study, multicentric study (two centres)	Right side, 6–15 MHz linear probe, intercostal approach (ZOA)	No, public domain image-processing program (ImageJ, National Institutes of Health, Bethesda, MD, USA)	ED_mean_, expressed in greyscale units ranging from 0 (black) to 255 (white) derived from both trace and square methods
	Coiffard 2021, Canada [40]	Critically ill patients undergoing MV (n=44) and healthy controls (n=10)	Observational study, multicentric study (two centres)	Right side, 6–15 MHz linear probe, intercostal approach (ZOA)	No, public domain image-processing program (ImageJ)	ED_50_, ED_85_ and HEA_65_, expressed in greyscale units ranging from 0 (black) to 255 (white) or percentage of pixel (for HEA_65_) derived from trace method
	Umbrello 2021, Italy [41]	Critically ill patients diagnosed with COVID-19, admitted with acute hypoxaemic respiratory failure (n=36)	Prospective observational study, single centre	Right side, 6–14 MHz linear probe, intercostal approach (ZOA)	No, public domain image-processing program (ImageJ)	ED_mean_, expressed in greyscale units ranging from 0 (black) to 255 (white) derived from trace method
	Formenti 2022, Italy [42]	Intubated ICU patients with confirmed COVID-19 and ARDS (n=32)	Prospective observational study, single centre	Right side, 6–14 MHz linear probe, intercostal approach (ZOA)	No, public domain image-processing program (ImageJ)	ED_mean_, expressed in greyscale units ranging from 0 (black) to 255 (white) derived from square method
	Fu 2022, China [43]	Patients undergoing major abdominal surgery (n=117)	Prospective observational study, single centre	Right side, high frequency linear probe, intercostal approach (ZOA)	No, public domain image-processing program (ImageJ)	ED_50_, ED_85_ and ED_mean_, expressed in greyscale units ranging from 0 (black) to 255 (white) derived from trace method
	van Doorn 2022, Netherlands [44]	Healthy subjects (n=83)	Retrospective observational study, single centre	Right side, 3–13 MHz linear probe (LA533), intercostal approach (ZOA)	No, custom-developed software in MATLAB (R2018a, Mathworks, Natick, MA, USA)	ED_mean_, expressed in greyscale units ranging from 0 (black) to 255 (white) derived from trace method
**Excursion of the zone of apposition**	Da Conceição 2024, Canada [45]	Elective surgery patients with normal diaphragmatic function (n=75)	Prospective observational study, single centre	Both left and right sides, 13 MHz linear probe, intercostal approach (ZOA)	Not needed	EXdi-ZOA (mm), calculations included the difference in diaphragm position from expiration to inspiration to quantify movement
**Shear wave elastography/strain elastography**	Chino 2018, Japan [46]	Healthy subjects (n=14)	Observational study (experimental design), single centre	Right side, 4–15 MHz linear probe (SL 15-4), intercostal approach (ZOA)	Yes	SMdi at end-inspiration (kPa), absolute values and “ratio value” relative to the shear modulus at resting end-expiration
	Bachasson 2019, France [47]	Healthy subjects (n=15)	Observational study (experimental design), single centre	Right side, 2–10 MHz linear probe (SL 10-2), intercostal approach (ZOA)	No, MATLAB (MathWorks)	ΔSMdi during inspiratory/contraction time minus end-expiratory value (kPa)
	Ando 2020, Japan [74]	Healthy subjects (n=19) (elite collegiate swimmers), training group (n=10) and control group (n=9)	Randomised controlled trial, single centre	Right side, 4–15 MHz linear probe (SL 15-4), intercostal approach (ZOA)	Yes	SMdi at end-inspiration (kPa), absolute values
	Ciloglu 2020, Turkey [48]	Patients with hyperkyphosis due to osteoporotic vertebral fracture (n=42) and healthy controls (n=36)	Prospective case–control study, single centre	Right side, high-resolution linear probe (Philips L5-18), intercostal approach (ZOA)	Yes	Strain (colour code), strain ratio at end-expiration and end-inspiration, no specific quantification^#^
	Flatres 2020, France [49]	Healthy subjects (n=31) and critically ill patients (n=12)	Prospective observational study, single centre	Right side, 4–15 MHz linear probe (SL 15-4), intercostal approach (ZOA)	Yes	SMdi at end-expiration (kPa)
	Fossé 2020, France [50]	Critically ill patients undergoing MV (n=30)	Prospective observational study, single centre	Right side, 2–10 MHz linear probe (SL 10-2), intercostal approach (ZOA)	No, MATLAB (MathWorks)	ΔSMdi during inspiratory/contraction time (kPa)
	Aarab 2021, France [51]	Critically ill patients (n=102)	Prospective observational study, single centre	Right side, 4–15 MHz linear probe (SL 15-4), intercostal approach (ZOA)	No, OsiriX DICOM Viewer software (Pixmeo, Geneva, Switzerland)	SMdi at end-expiration (kPa)
	Xu 2021, China [52]	Patients with stable COPD (n=42) and healthy controls (n=34)	Prospective observational study, single centre	Right side, 9 MHz linear probe (SL 15-4), intercostal approach (ZOA)	Yes	SWVdi at end-expiration (m·s^−1^)
	Chen 2022, China [53]	Patients with COPD (n=219) and healthy adults (n=20)	Prospective observational study, single centre	Right side, 4–15 MHz linear probe (SL 15-4), intercostal approach (ZOA)	Yes	SMdi at end-inspiration (kPa)
	Şendur 2022, Turkey [54]	Healthy volunteers (n=40) and patients with COPD (n=8)	Prospective observational study, single centre	Right side, 9-MHz linear probe, intercostal approach (ZOA)	Yes	SMdi at end-expiration and end-inspiration (kPa)
	Zhang 2023, China [55]	Patients with acute exacerbation of COPD (n=112)	Prospective observational study, single centre	Right side, 9-MHz linear probe, intercostal approach (ZOA)	Yes	SMdi at end-expiration and % change at end-inspiration (%SMdi) (kPa)
	Zhang 2024, China [56]	Healthy subjects with normal spirometry (n=212)	Prospective observational study, single centre	Right side, 3–11 MHz linear probe, intercostal approach (ZOA)	Yes	SMdi (timing of the respiratory cycle not defined): mean, maximum, minimum, sd values recorded (kPa)
**Speckle tracking**	Ye 2013, China [57]	Healthy volunteers (n=21)	Observational study (cross-sectional), single centre	Right side, 2–4 MHz phased array probe (M5S), subcostal approach (midclavicular alignment)	No, EchoPAC (GE HealthCare, Milwaukee, MI, USA)	Strain (%), Dlcos (%), Dldome (%), Dlcru (%)
	Hatam 2014, Germany [58]	Healthy volunteers (n=13)	Observational study, single centre	Right side, 2–4 MHz phased array probe (M5S), intercostal approach (anterior axillary line)	No, EchoPAC (GE HealthCare)	Inspiratory peak longitudinal strain (–%) and peak transverse strain (+%); inspiratory peak longitudinal strain rate (–1·s^−1^) and peak transverse strain rate (+1·s^−1^); expiratory peak longitudinal strain rate (+1·s^−1^); cranio-caudal displacement (mm)
	Orde 2016, USA [59]	Healthy volunteers (n=50)	Observational study, single centre	Right side, 2.5–8 MHz linear array probe, intercostal approach (ZOA)	No, EchoPAC (GE HealthCare)	Strain (%), calculated as strain=(D2−D1)/D1×100, where D1 is expiratory time and D2 is inspiratory time
	Goutman 2017, USA [60]	Healthy volunteers (n=6)	Observational study, single centre	Both left and right sides, curved probe (C5-1), intercostal approach (mid-axillary line)	No, EchoInsight software (Epsilon Imaging, Ann Arbor, MI, USA)	Diaphragm movement (excursion) (cm) in two dimensions: cephalocaudad and mediolateral
	Oppersma 2017, Netherlands [61]	Healthy volunteers (n=15)	Observational study (experimental design), single centre	Right side, 9 MHz linear probe, intercostal approach (ZOA)	No, EchoPAC (GE HealthCare)	Strain (%), which represents the relative change in length from an initial state, and strain rate (s^−1^), which measures the rate of deformation and is an instantaneous measurement
	Fritsch 2022, Germany [62]	Patients undergoing elective CABG surgery (n=20)	Observational study, single centre	Right side, 9 MHz linear probe, intercostal approach (ZOA)	No, EchoPAC (GE HealthCare)	Strain (%), which represents the relative change in length from an initial state, and strain rate (s^−1^), which measures the rate of deformation and is an instantaneous measurement
	Xu 2022, China [63]	Critically ill patients (n=116) and healthy subjects (n=25)	Prospective and retrospective observational study, multicentric study (two centres)	Right side, 4.0–13.0 MHz or 4.0–11.0 MHz linear probe, intercostal approach (ZOA)	No, EchoPAC (GE HealthCare)	Strain (%), which represents the relative change in length from an initial state
	Li 2024, China [64]	Critically ill patients (n=86)	Prospective observational study, single centre	Right side, phased array probe (SP5-1s), subcostal approach (midclavicular alignment)	Yes	Strain (%), Dlcos (%), Dldome (%), Dlcru (%)
	Watanabe 2024, Japan [65]	Patients with ALS (n=19) and healthy controls (n=21)	Prospective cohort study, single centre	Right side, 10 MHz linear probe (L4-12t-RS), intercostal approach (ZOA)	No, dedicated prototype software developed in Microsoft Visual C++	DMD (mm), strain of the diaphragm (%)
**Tissue Doppler imaging**	Fayssoil 2019, France [66]	Patients with genetically confirmed neuromuscular diseases (n=89) and healthy adult individuals (n=27)	Retrospective observational study, single centre	Both left and right sides, phased array probe, subcostal approach between the midclavicular and anterior axillary lines	Yes	Inspiratory moment: PCVdi (cm·s^−1^)
	Soilemezi 2020, Greece [67]	Critically ill ICU patients (n=116) and healthy volunteers (n=20)	Prospective observational study, single centre	Right side, 2–4 MHz phased array probe, subcostal approach between the midclavicular and anterior axillary lines	Yes	Inspiratory moment: PCVdi (cm·s^−1^), VTIdi (cm) Expiratory moment: PRVdi (cm·s^−1^), MRRdi (cm·s^−2^)
	Cammarota 2021, Italy [68]	Critically ill patients under MV (n=100)	Prospective observational study, single centre	Right side, 1.8–4.2 MHz phased array probe, subcostal approach between the midclavicular and anterior axillary lines	Yes	Inspiratory moment: PCVdi (cm·s^−1^), VTIdi, (cm), MCVdi (cm·s^−1^), inspiratory acceleration (cm·s^−2^), Expiratory moment: PRVdi (cm·s^−1^), MRRdi (cm·s^−2^), expiratory mean velocity (cm·s^−1^)
	Benli 2024, Turley [69]	Critically ill patients undergoing MV for >2 days (n=20) and healthy individuals (n=10)	Randomised controlled trial, single centre	Right side, 2.7 MHz curved probe, subcostal approach between the midclavicular and anterior axillary lines	Yes	Inspiratory moment: PCVdi (cm·s^−1^) Expiratory moment: PRVdi (cm·s^−1^)
	Xin 2024, China [70]	Critically ill patients in ICU undergoing MV for >48 h (n=89)	Prospective observational study, single centre	Right side, 2–4 MHz phased array probe, subcostal approach between the midclavicular and anterior axillary lines	Yes	Inspiratory moment: PCVdi (cm·s^−1^), mean contraction velocity (cm·s^−1^), inspiratory acceleration (cm·s^−2^) Expiratory moment: PRVdi (cm·s^−1^), MRRdi (cm·s^−2^)

ALS: amyotrophic lateral sclerosis; ARDS: acute respiratory distress syndrome; AU: acoustic unit; CABG: coronary artery bypass graft; COVID-19: coronavirus disease 2019; Dlcos: costal/zone of apposition diaphragm strain; Dlcru: crural diaphragm strain; Dldome: diaphragmatic dome strain; DMD: diaphragm moving distance; ED_50_: 50th percentile of the echogenicity/echodensity greyscale; ED_85_: 85th percentile of the echogenicity/echodensity greyscale; ED_mean_: mean echogenicity/echodensity greyscale value; EXdi-ZOA: excursion of the zone of apposition; HEA_65_: percentage of pixels above the echogenicity/echodensity greyscale value of 65; ICU: intensive care unit; LVAD: left ventricular assist device; MCVdi: diaphragm mean contraction velocity; MRRdi: maximal relaxation rate; MV: mechanical ventilation; PCVdi: peak contraction velocity; PRVdi: peak relaxation velocity; SMdi: shear modulus of diaphragm; ΔSMdi: difference between end-inspiratory and end-expiratory diaphragm shear modulus; %SMdi: percentage change of diaphragm shear modulus from end-expiration to end-inspiration; SWVdi: diaphragm shear wave velocity; VTIdi: velocity-time integral; ZOA: zone of apposition. ^#^: from strain elastography.

### Results of individual studies

Each new ultrasound marker and the results of individual studies are presented according to the various DUS techniques identified in the literature and summarised in tables 1 and 2.

**TABLE 2:** Main results of the included studies (reliability/feasibility, physiological outcomes, clinical outcomes)

Innovative ultrasound technique	Publication, country	Reliability/physiological outcomes/clinical outcomes
**Area method**	Skaarup 2018, Denmark [35]	**Reliability:** Inter-rater reliability was high, with an ICC of 0.90 (p<0.001). Five novice operators conducted ultrasound assessment. The diaphragm was successfully visualised on both the right and left sides from a mid-axillary view in 100% of examinations (95% CI 69–100%), which is used for the area method. However, they could not obtain images of the left and right diaphragm at the mid-clavicular line in 80% (95% CI 44–97%) and 10% (95% CI 0–45%) of cases, respectively. **Physiological outcomes:** The area method correlated strongly with expired lung volume (0.88, 95% CI 0.81–0.95), slightly outperforming M-mode (0.84, 95% CI 0.75–0.92). In low volume, correlations remained similar, but in high volume, both methods showed decreased correlation: area method at 0.29 (95% CI 0.07–0.64) and M-mode at 0.43 (95% CI 0.17–0.68). **Clinical outcomes:** N/A
	Skaarup 2020, Italy [71]	**Reliability:** N/A **Physiological outcomes:** N/A **Clinical outcomes:** The affected hemidiaphragm's movement, assessed by the area method, significantly improved after thoracentesis, increasing from 7.4 cm^2^ (95% CI 5.14–9.56 cm^2^) to 26.0 cm^2^ (95% CI 20.81–31.13 cm^2^, p<0.0001). In contrast, the unaffected side showed a minor, nonsignificant increase from 26.3 cm^2^ (95% CI 21.00–31.63 cm^2^) to 27.9 cm^2^ (95% CI 20.35–35.47 cm^2^, p=0.52).
	Fjaellegaard 2024, Denmark [72]	**Reliability:** N/A **Physiological outcomes:** Area method and conventional M-mode EXdi before and after thoracentesis presented very poor correlations (highest coefficient of 0.06). **Clinical outcomes:** No association between baseline diaphragm movement measured by the area method and being a responder to thoracentesis (OR 1.04, 95% CI 0.97–1.11).
	Nørskov 2024, Denmark [73]	**Reliability:** N/A **Physiological outcomes:** N/A **Clinical outcomes:** Diaphragmatic caudal displacement measured by the area method showed a significant reduction in intrathoracic area on the surgical side (p<0.001), with a mean reduction of −18.9 cm^2^ (95% CI −12.3– −25.4 cm^2^) from the day before surgery to 3 days after, and −13.3 cm^2^ (95% CI −5.2– −21.3 cm^2^) to 10–14 days post-surgery. No significant change was noted on the nonsurgical side (p=0.88).
	Petersen 2024, Denmark [36]	**Reliability:** N/A **Physiological outcomes:** N/A **Clinical outcomes:** Area method diagnostic properties for non-expandable lung post-thoracentesis: the AUC for the area method's Δ was 0.60 (95% CI 0.40–0.79), lower than M-mode lung movement, M-mode diaphragm movement and B-mode diaphragm movement, and only higher than shear wave elastography for parietal pleura, pleural effusion and visceral pleura.
	Skaarup 2024, Denmark [37]	**Reliability:** N/A **Physiological outcomes:** Compared to fluoroscopy, the left hemidiaphragm showed a regression coefficient of 0.10 (95% CI 0.08–0.12) and Pearson correlation of 0.48 during IC, and 0.04 (95% CI 0.03–0.05) with Pearson correlation of 0.20 during sniff manoeuvre. For the right hemidiaphragm, the coefficients were 0.10 (95% CI 0.07–0.11) with Pearson correlation of 0.34 during IC, and 0.05 (95% CI 0.03–0.07) with Pearson correlation of 0.02 during sniff manoeuvre. **Clinical outcomes:** N/A
**Contrast-enhanced ultrasound**	Bird 2024, Canada [38]	**Reliability:** *Q̇*_DIA_ showed good to excellent test–retest reliability (ICC 0.86, 95% CI 0.77–0.92) and excellent inter-analyser reproducibility (ICC 0.93, 95% CI 0.90–0.95). **Physiological outcomes:** During four respiratory loading stages (unloaded, 10%, 18% and 25% of MIP), *Q̇*_DIA_ increased with each stage (3.1±3.1, 6.9±3.6, 11.0±4.9 and 13.5±5.4 AU·s^−1^, respectively; p<0.0001). MFR_DIA_ and *Q̇*_DIA_ increased with load but were consistent across days. Increased *P*di correlated with higher *Q̇*_DIA_ and VC_DIA_ (p<0.0001; η_p_^2^>0.94), also showing reproducibility across days (p>0.34). **Clinical outcomes:** N/A
**Echogenicity/echodensity**	Sarwal 2015, Australia and USA [39]	**Reliability:** No significant differences in diaphragm muscle echodensity were found between novice and experienced raters using either the trace (p=0.86) or square method (p=0.57). **Inter-observer reliability:** ICC values ranged from 0.851 (95% CI 0.667–0.938) to 0.984 (95% CI 0.959–0.993). A significant difference in mean echodensity was observed between the square and trace methods (1.14, 95% CI 0.23–2.05; p=0.02), with novice raters showing wider limits of agreement compared to experienced assessors. **Physiological outcomes:** N/A **Clinical outcomes:** N/A
	Coiffard 2021, Canada [40]	**Reliability:** Echodensity between analysers (one image) showed an average difference of −1.5 (limits −8.6 to 5.7) based on 30 images. Between images (two from the same patient), the average difference was −2.8 (limits −15.8 to 10.2). Echodensity at end-expiration and end-inspiration (same respiratory cycle) had an average difference of −1.3 (limits −9.8 to 7.2). **Physiological outcomes:** Both increases and decreases in Tdi from baseline and during follow-up correlated with increases in ED_50_ over time (within-subject R^2^=0.78, p=0.03). On day 1 of ventilation, cumulative fluid balance had a correlation coefficient of −0.2 (95% CI −1.4–1.0, p=0.72) and on day 3, it was 1.2 (95% CI −1–3, p=0.24). No significant differences in cumulative fluid balance were found on day 1 (2.1 L, IQR 0.1–5.1 L *versus* 0.3 L IQR 0.7–0.9 L, p=0.36) or day 3 (3.7±6.2 L *versus* 4.6±3.5 L, p=0.60) between patients with a ≤10-point change in ED_50_ and those with a >10-point increase. **Clinical outcomes:** Patients on MV had higher diaphragm echodensity than healthy subjects at both ED_50_ and ED_85_ (p=0.07 and 0.04, respectively). Baseline ED_50_ was not linked to ventilator-free days by day 60, ICU duration or mortality. Increased echodensity (>10-point increase in ED_50_) occurred in 13 patients (38%), with no association with patient characteristics. Patients with increased echodensity had fewer ventilator-free days by day 60 (median 46 days, IQR 0–52 days *versus* median 53 days, IQR 49–56 days, p=0.03) and were more likely to require ventilation for ≥7 days (85% *versus* 33%, p=0.01). Patients needing ventilation for ≥7 days showed significant increases in ED_50_ on day 2 (+40% from baseline, IQR +4–54%) compared to those needing <7 days (−9% change, IQR −45–+4%, p=0.007), with the association persisting after adjusting for Tdi changes (adjusted p=0.01).
	Umbrello 2021, Italy [41]	**Reliability:** Intra-rater ICC for diaphragm echodensity was 0.998 (95% CI 0.996–0.999), and inter-rater ICC was 0.998 (95% CI 0.997–0.999). **Physiological outcomes:** Changes in diaphragm echodensity during follow-up were positively related to cumulative fluid balance (R^2^=0.417, p<0.001) but not to cumulative protein deficit (R^2^=0.083, p=0.137). **Clinical outcomes:** Diaphragm echodensity increased during the first 7 days of ICU stay (p<0.0001). The increase in diaphragm echodensity from baseline to day 7 was greater in non-survivors; at admission, the percentage change for survivors was 0.1% (IQR −12.6–17.5%), while for non-survivors, the percentage change was 14.6% (IQR 9.5–24.3%) (p=0.0169).
	Formenti 2022, Italy [42]	**Reliability:** N/A **Physiological outcomes:** Right diaphragm echodensity correlated with right parasternal intercostal muscle echodensity (R^2^=0.3225, p=0.001) and cumulative fluid balance (R^2^=0.315, p=0.001). **Clinical outcomes:** Right diaphragm echodensity differed between survivors and non-survivors, with medians of 65 (IQR 62.6–68) and 77 (IQR 74.2–94) in greyscale, respectively (p=0.0002).
	Fu 2022, China [43]	**Reliability:** Between analysers, the average difference for ED_50_ was 1.63 (limits: −17 to 20, p=0.158), for ED_85_ it was 3.24 (−32 to 38) and for ED_mean_ it was 1.24 (−19 to 22). Between images, the difference in ED_50_ was 0.30 (−8 to 8, p=0.700), while ED_85_ was −0.14 (−13 to 13) and ED_mean_ was −0.07 (−7 to 7). Within a single respiratory cycle, the ED_50_ difference between end-expiration and end-inspiration was 0.50 (−7 to 8, p=0.476), while ED_85_ was 2.14 (−12 to 17) and ED_mean_ was 1.94 (−4 to 8). **Physiological outcomes:** N/A **Clinical outcomes:** Patients who developed PPCs had significantly higher echodensity values: ED_50_ (p<0.001), ED_85_ (p<0.001) and ED_mean_ (p<0.001). ROC curve analysis showed AUCs for ED_50_, ED_mean_ and ED_85_ predicting PPCs of 0.611 (95% CI 0.516–0.699), 0.603 (95% CI 0.508–0.692) and 0.612 (95% CI 0.517–0.700), respectively, with an optimal ED_50_ cut-off of 36. Univariate logistic regression identified diaphragm echodensity as a factor linked to PPCs, with ORs of 1.032 for ED_mean_, 1.037 for ED_50_ and 1.018 for ED_85_. Multivariable analysis confirmed that higher diaphragm echodensity was independently associated with PPCs: ED_mean_ OR 1.026, 95% CI 1.022–1.029; ED_50_ OR 1.032, 95% CI 1.027–1.036; and ED_85_ OR 1.014, 95% CI 1.012–1.017 (all p<0.001).
	van Doorn 2022, Netherlands [44]	**Reliability:** Intra-observer reliability for end-expiration echodensity was ICC 0.93 (95% CI 0.89–0.96). **Physiological outcomes:** N/A **Clinical outcomes:** Regression analyses showed that echodensity increased with age (p<0.001), described by the formula “Echodensity=71.443+0.390×cAge,” where cAge is the centred age, calculated by subtracting 39.0 from age in years.
**Excursion of the zone of apposition**	Da Conceição 2024, Canada [45]	**Reliability:** The evaluation of EXdi-ZOA was consistently successful (100% bilaterally) compared to the conventional excursion of the DOD (98.7% on the right and 34.7% on the left). **Physiological outcomes:** DOD excursion between sides showed a CC of 0.57 (p<0.01). EXdi-ZOA between sides had a CC of 0.62 (p<0.001). The CC between EXdi-ZOA and DOD measurements was 0.28 on the right and 0.42 on the left (p<0.05). No relation was found between either assessment and TFdi. **Clinical outcomes:** N/A
**Shear wave elastography/strain elastography**	Chino 2018, Japan [46]	**Reliability:** During submaximal sustained inspiratory efforts at target levels, the intra-subject coefficient of variation for SMdi ranged from 3.7% to a maximum of 5.9%. **Physiological outcomes:** Significant differences in the relative ratio of SMdi were observed across five inspiratory target levels (p<0.001; η_p_^2^=0.89), with all *post hoc* comparisons differing significantly (p≤0.007; Cohen's d=0.54–3.39). The relationship between inspiratory mouth pressure and SMdi yielded coefficients of determination (R^2^) of 0.94±0.05 for eight out of 14 subjects (R^2^≥0.95) and 0.99±0.01 for all subjects (R^2^≥0.95) for simple linear and second-order polynomial equations, respectively. **Clinical outcomes:** N/A
	Bachasson 2019, France [47]	**Reliability:** The mean coefficient of variation for SMdi was 16.2% during isovolumetric inspiratory effort against closed airways. **Physiological outcomes:** During isovolumetric inspiratory effort, mean *P*di significantly correlated with mean ΔSMdi across participants, with CCs ranging from 0.77 to 0.96 (all p<0.01; r=0.82, 95% CIs 0.76–0.86). During ventilation against inspiratory threshold loading, maximal ΔSMdi correlated with the *P*di swing in all participants, with coefficients ranging from 0.40 to 0.90 (all p<0.01; r=0.70, 95% CIs 0.66–0.73, p<0.001). **Clinical outcomes:** N/A
	Ando 2020, Japan [74]	**Reliability:** N/A **Physiological outcomes:** N/A **Clinical outcomes:** After 6 weeks, at inspiratory mouth pressures of 15%, 45% and 75% of MIP, the control group had increased SMdi from 58.7±16.8, 96.9±33.2 and 146.4±55.7 kPa pre-training to 70.6±21.8, 138.2±39.1 and 194.8±47.1 kPa post-training. The training group had increased SMdi from 59.2±17.9, 108.9±24.0 and 149.9±31.1 kPa to 71.0±24.3, 137.9±39.3 and 186.7±49.4 kPa. Based on a linear regression equation, SMdi during the MIP manoeuvre (100%) significantly increased in both groups (p<0.05), from 188.1±21.9 kPa to 257.0±22.1 kPa in controls and 197.5±12.1 kPa to 248.7±20.1 kPa in training.
	Ciloglu 2020, Turkey [48]	**Reliability:** The ICCs for end-inspiration and end-expiration strain ratios were 0.897 and 0.926, indicating good to excellent intra-observer reliability. Agreement for colour grades in strain elastography was very high, with Kendall's τ-values of 0.984 for expiration and 0.952 for inspiration (p<0.001). **Physiological outcomes:** Strain ratio values correlated inversely with FEV_1_ (%) (r= −0.929, p<0.001) and FVC (%) (r= −0.791, p<0.001). Strain ratio (% change) values positively correlated with Cobb angle (r=0.905, p<0.001) and number of vertebra fractures (r=0.782, p<0.001). **Clinical outcomes:** Strain ratio values were significantly higher in the kyphosis group at end-inspiration (3.56±0.1 *versus* 2.88±0.1, p<0.001) and for percentage change (0.58±0.1% *versus* 0.27±0.1%, p<0.001). The rate of the hardest colour code was significantly higher in the control group (p<0.001).
	Flatres 2020, France [49]	**Reliability:** In the training set of healthy subjects (n=16), the longitudinal view (transducer parallel to fibres) yielded means of 19.4±6.2 kPa for Operator 1 and 20.1±7 kPa for Operator 2, with inter-operator reproducibility (ICC) at 0.83 (95% CI 0.50–0.94). In the transverse view (transducer perpendicular to fibres), means were 25.4±7.3 kPa for Operator 1 and 22.4±6.3 kPa for Operator 2, with ICC at 0.3 (95% CI −0.86–0.75). In the validation set of healthy subjects (n=15), means were 20±7.3 kPa for Operator 1 and 20.6±6.1 kPa for Operator 2, with ICC at 0.96 (95% CI 0.85–0.99). Intra-operator reliability was 0.95 (95% CI 0.82–0.99) for Operator 1 and 0.90 (95% CI 0.70–0.98) for Operator 2. For critically ill patients (n=12), means were 13.1±4.2 kPa for Operator 1 and 14.2±4.6 kPa for Operator 2, with ICC at 0.92 (95% CI 0.71–0.98). Intra-operator reliability was 0.93 (95% CI 0.82–0.98) for Operator 1 and 0.92 (95% CI 0.81–0.98) for Operator 2. **Physiological outcomes:** N/A **Clinical outcomes:** N/A
	Fossé 2020, France [50]	**Reliability:** N/A **Physiological outcomes:** A correlation was found between Δ*P*di and ΔSMdi (r=0.45, 95% CIs 0.35–0.54, p<0.001). Among 25 patients, significant correlations were observed in eight cases (r=0.55–0.86, all p<0.05), while the remaining correlations ranged from r= −0.43 to 0.52 and were nonsignificant (all p>0.06). Patients with a nonsignificant Δ*P*di–ΔSMdi correlation had a higher respiratory rate (median 25, IQR 18–33 breaths·min^−1^) compared to those with a significant correlation (median 21, IQR 15–26 breaths·min^−1^). **Clinical outcomes:** Significant differences in ΔSMdi values were noted between conditions: SBT–Start (median 12.2 kPa, IQR 7.7–14.3 kPa) and SBT-End (median 7.5 kPa, IQR 4.8–13.1 kPa) were significantly different from PS+25% inspiratory pressure support (median 5.5 kPa, IQR 3.8–9.0 kPa), PS (median 5.4 kPa, IQR 3.5–8.8 kPa), PS−25% inspiratory pressure support (median 7.0 kPa, IQR 5.8–8.6 kPa) and PS with baseline inspiratory support and zero end-expiratory pressure (median 7.7 kPa, IQR 4.0–11.8 kPa), all with p<0.05.
	Aarab 2021, France [51]	**Reliability:** N/A **Physiological outcomes:** N/A **Clinical outcomes:** SMdi varied among patients: stable in 8%, decreased by >10% in 41%, and increased by >10% in 51%. Multivariable analysis showed that SMdi declined over time in older patients (β −0.05±0.02, p<0.05), those treated for sepsis (β −1.79±0.82, p=0.03) and those receiving steroids (β −0.11±0.04, p=0.01). Patients with increased Tdi during ICU stay also had reduced SM (β −8.92±4.46, p=0.03). The duration of controlled MV was linked to decreased SMdi compared to pressure support or no ventilatory assistance (β 1.45±0.40, p<0.05). No significant correlations were found between changes in diaphragm SM and ventilator-free days, ICU length of stay, weaning difficulties or mortality at day 28.
	Xu 2021, China [52]	**Reliability:** Intra-observer ICC for SWVdi at FRC was 0.93 (95% CI 0.82–0.98). **Physiological outcomes:** SWVdi at FRC correlated with FEV_1_ (r= −0.30, p=0.009) and FVC (r= −0.33, p=0.003). **Clinical outcomes:** SWVdi at FRC correlated with the mMRC score (r=0.30, p=0.001) and the CAT score (r=0.48, p<0.001). In the COPD group, median SWVdi was 2.5 m·s^−1^ (IQR 2.3–2.7 m·s^−1^) *versus* 2.1 m·s^−1^ (IQR 1.8–2.5 m·s^−1^) in controls (p=0.008). SWVdi in controls was significantly lower than in patients with severe COPD (p=0.021), but no significant difference was found between controls and mild–moderate COPD (p=0.333). Mild–moderate COPD did not differ from severe COPD in SWVdi (p=1.000).
	Chen 2022, China [53]	**Reliability:** Intra-observer reliability for end-inspiratory SMdi had an ICC of 0.756 (95% CI 0.482–0.896) and inter-observer reliability had an ICC of 0.775 (95% CI 0.511–0.905), based on data from healthy controls. **Physiological outcomes:** In patients with COPD, end-inspiratory SMdi significantly correlated with pulmonary function parameters: FEV_1_/FVC (r= −0.81), predicted FEV_1_% (r= −0.63), RV (r=0.65), TLC (r=0.54), RV/TLC ratio (r=0.60), FRC (r=0.72) and IC (r= −0.41) (all p<0.001). These correlations were stronger than those for intercostal muscle stiffness (r= −0.76– −0.33, all p<0.001). Additionally, SMdi was positively correlated with intercostal muscle shear modulus (r=0.56, p<0.001). **Clinical outcomes:** In patients with COPD, end-inspiratory SMdi increased with disease severity (F=224.50, p<0.001). No significant differences in SMdi were found between groups with severe and with very severe COPD, but SMdi increased with COPD severity in the other groups.
	Şendur 2022, Turkey [54]	**Reliability:** SMdi inter-observer reliability showed ICCs of 0.667 (95% CI 0.452–0.809) at peak inspiration and 0.736 (95% CI 0.553–0.851) at end expiration. **Physiological outcomes:** In patients with COPD, Tdi significantly increased at peak inspiration (2.69±0.46 mm *versus* 2.25±0.35 mm, p=0.012), but no significant difference in SMdi measurements between respiratory phases was observed (35.83±8.64 kPa *versus* 33.25±10.76 kPa, p>0.05). **Clinical outcomes:** N/A
	Zhang 2023, China [55]	**Reliability:** Intra-observer ICC was 0.789 (95% CI 0.584–0.915) and inter-observer ICC was 0.727 (95% CI 0.355–0.900). **Physiological outcomes:** In the “high-risk, few symptoms” group, SMdi had moderate negative correlations with TFdi and FEV_1_/FVC (r= −0.408– −0.492, p<0.05) and weak negative correlations with FEV_1_ and FVC (r= −0.293– −0.373, p<0.05). %SMdi (% change) also showed moderate negative correlations with TFdi and FEV_1_/FVC (r= −0.429– −0.430, p<0.05) and weak negative correlations with FEV_1_ and FVC (r= −0.308, p<0.05). In the “high-risk, many symptoms” group, SMdi had strong negative correlations with TFdi (r= −0.697, p<0.000) and moderate negative correlations with FEV_1_/FVC (r= −0.538, p<0.001), along with weak negative correlations with FEV_1_ and FVC (r= −0.306– −0.373, p<0.05). %SMdi also had strong negative correlations with TFdi and FEV_1_/FVC (r= −0.623– −0.697, p=0.000) and weak negative correlations with FEV_1_ and FVC (r= −0.304– −0.386, p<0.05). **Clinical outcomes:** In a comparison of two high-risk groups, the “high-risk, many symptoms” group had significantly higher SMdi (median 20.58 kPa) and %SMdi (mean 0.50) than the “high-risk, few symptoms” group (SMdi median 18.52 kPa, %SMdi mean 0.41; p=0.040 and p=0.004, respectively). In the “few symptoms” group, SMdi showed weak positive correlations with the CAT (r=0.306) and mMRC scores (r=0.274), while %SMdi correlated positively with both scores (r=0.303–0.398). In the “many symptoms” group, both SMdi and %SMdi had weak positive correlations with the CAT score (r=0.302–0.395), and SMdi correlated with the mMRC score (r=0.349). %SMdi showed a moderate correlation with the mMRC score (r=0.462).
	Zhang 2024, China [56]	**Reliability:** N/A **Physiological outcomes:** The study population exhibited the following results for SMdi descriptors: 16.72±4.07 kPa for SMdi mean value descriptor, 25.04±5.58 kPa for SMdi maximum value descriptor, 11.06±3.88 kPa for SMdi minimum value descriptor and 2.56±0.98 kPa for SMdi sd value descriptor. **Clinical outcomes:** No significant differences were found in mean SMdi values across age groups, with averages of 17.22 kPa (young), 16.06 kPa (adult) and 16.86 kPa (older adult) (p=0.159). Similarly, BMI groups showed no significant variation in mean SMdi values, ranging from 16.01 kPa (low weight) to 17.12 kPa (normal weight) (p=0.506). Lifestyle groups also exhibited no significant differences in mean SMdi values, with the physically active group averaging 17.29 kPa (p=0.226). SMdi maximum, minimum and sd values across all groups had comparable differences (p-values=0.107–0.773). Overall, the analysis suggests that SMdi values and their variability are similar regardless of age, BMI or lifestyle factors.
**Speckle tracking**	Ye 2013, China [57]	**Reliability:** N/A **Physiological outcomes:** Diaphragm deformation patterns revealed strain values during quiet breathing: Dlcru at −5.24±3.00%, Dldome at 3.24±1.64% and Dlcos at −6.24±2.91%. During forced breathing, Dlcru decreased to −7.42±5.10% (p=0.0709), Dldome to 4.10±2.34% (p=0.2780) and Dlcos to −10.00±4.58% (p=0.0051). Overall diaphragm strain was −2.14±1.80% for quiet breathing and −4.62±2.56% for forced breathing (p=0.0002). No significant difference was found in the right diaphragm's crura and ZOA during quiet breathing (p=0.198), but a significant difference emerged during forced breathing (p=0.024). **Clinical outcomes:** N/A
	Hatam 2014, Germany [58]	**Reliability:** N/A **Physiological outcomes:** Transverse strain values showed a strong correlation with TFdi, with both TID peak strain and TFdi significantly increasing during continuous positive airway pressure and PSV (Pearson's r=0.753, p<0.001). The peak transverse strain rate also significantly increased in the treatment groups. However, longitudinal measurements did not show significant differences across ventilator settings. Isolated correlation analyses for positive end-expiratory pressure and PSV indicated weaker correlations for longitudinal displacement. Additionally, there were no significant changes in the peak expiratory longitudinal strain rate or the time to peak expiratory strain rate based on inspiratory duration. **Clinical outcomes:** N/A
	Orde 2016, USA [59]	**Reliability:** Inter-observer reliability for longitudinal strain was ICC 0.90 (95% CI 0.61–0.98) with a coefficient of repeatability of 24.3%. Intra-observer reliability was ICC 0.96 (95% CI 0.88–0.99) with a coefficient of repeatability of 19.4%.
		**Physiological outcomes:** Two-dimensional ST imaging showed an average longitudinal strain value of the right diaphragm at −40.3±9%, with a moderate correlation with TFdi (R^2^=0.44, p<0.0001) and a very weak correlation with caudal displacement (EXdi) (R^2^=0.14, p<0.01). **Clinical outcomes:** N/A
	Goutman 2017, USA [60]	**Reliability:** ST method visualised the left hemidiaphragm in all six subjects during normal breathing and five during deep breathing, while M-mode visualised it in only two subjects during normal breathing and none during deep breathing. **Physiological outcomes:** The average differences in right hemidiaphragm measurements were 0.30 cm for normal inspiration and −0.65 cm for deep inspiration. For the left hemidiaphragm, the average difference during normal inspiration was 1.00 cm. **Clinical outcomes:** N/A
	Oppersma 2017, Netherlands [61]	**Reliability:** N/A **Physiological outcomes:** During the inspiratory threshold loading protocol (0–50% MIP), both strain and strain rate significantly increased with load (p<0.001). Strain rose from −22±7.6% at zero loading to −41.5±10.1% at 50% loading, while strain rate increased from −0.48±0.2 s to −1.5±0.7 s. ST showed superior assessment of diaphragm contractility, with strong correlations to *P*di (strain R^2^=0.72; strain rate R^2^=0.80) and diaphragm electrical activity (strain R^2^=0.60; strain rate R^2^=0.66). No significant correlations were found between TFdi and strain (p=0.654) or strain rate (p=0.364). **Clinical outcomes:** N/A
	Fritsch 2022, Germany [62]	**Reliability:** N/A **Physiological outcomes:** N/A **Clinical outcomes:** Within 24 h post-extubation, all patients showed a decrease in strain during basal respiration, with median strain dropping to 73% of preoperative levels. By 48 h, strain values nearly returned to baseline, with significant changes (p<0.001). The median strain rate remained stable initially but significantly increased at 48 h, with most patients recovering to or exceeding preoperative levels (p=0.010 compared to preoperative; p<0.001 compared to the first assessment). No correlations were found between strain or strain rate and age, BMI or MV duration. However, a negative correlation was observed between diaphragm deformation and fluid volume administered in the first 24 h in the ICU, with Spearman coefficients of −0.531 for strain and −0.495 for strain rate at 48 h post-extubation (p=0.023 for strain; p=0.037 for strain rate). A similar nonsignificant trend was noted 24 h earlier.
	Xu 2022, China [63]	**Reliability:** In healthy volunteers at rest, intra-operator reliability was good, with ICC values of 0.86 (95% CI 0.63–0.95) for the first operator and 0.87 (95% CI 0.74–0.94) for the second. Inter-operator reliability was also good at 0.87 (95% CI 0.73–0.94). During deep breathing, intra-operator reliability remained good (ICCs: 0.84 for the first operator, 0.80 for the second), while inter-operator reliability was 0.78. In patients on MV, intra-operator reliability was excellent (ICCs: 0.95 for the first operator, 0.92 for the second), and inter-operator reliability was also excellent at 0.94. **Physiological outcomes:** Diaphragmatic strain showed a strong linear relationship with TFdi (R^2^=0.73, p<0.0001) and EXdi (R^2^=0.61, p<0.0001), but a weak relationship with expiratory and inspiratory thicknesses (R^2^=0.01, p=0.3336; R^2^=0.07, p=0.0071). **Clinical outcomes:** Differences were found between success and failure groups for strain (%) (success: −25.00%, IQR −34– −16%; failure: −13.00%, IQR −18.00– −7.00%; p<0.001), TFdi (success: 25.40%, IQR 21.18–32.84%; failure: 20.82%, IQR 17.05–25.86%; p<0.001) and EXdi (success: 14.50 mm, IQR 11.55–19.50 mm; failure: 10.80 mm, IQR 8.20–13.30 mm; p<0.001). AUC values for strain, Rapid Shallow Breathing Index, TFdi and EXdi in predicting successful weaning were 0.794, 0.794, 0.723 and 0.728, respectively. Optimal cut-off values for predicting weaning success were: Strain <−21% (sensitivity 89.19%, specificity 64.41%), TFdi >83% (sensitivity 59.46%, specificity 88.14%) and EXdi >11.2 mm (sensitivity 56.76%, specificity 79.66%).
	Li 2024, China [64]	**Reliability:** N/A **Physiological outcomes:** N/A **Clinical outcomes:** Significant differences were found between the successful and the weaning failure groups regarding strain values after SBT. The failure group exhibited lower levels of “whole strain” (p<0.001), Dlcos (p<0.001), Dlcru strain (p=0.001), EXdi (p<0.001) and TFdi (p<0.001). ROC curve analysis showed that a Dlcos value >−9.836% had an AUC of 0.760, with 80% sensitivity and 72.5% specificity for predicting successful weaning. An EXdi value >1.015 cm had an AUC of 0.785, with high specificity (90.2%) but lower sensitivity (60%). Combining EXdi with whole strain increased the AUC to 0.856, achieving balanced sensitivity (80%) and specificity (80.4%). Univariate regression analyses indicated strong associations between whole strain, Dlcos, Dlcru, EXdi and TFdi with weaning outcomes. Multivariate logistic regression identified whole strain (OR 1.962, 95% CI 1.042–3.655, p=0.037) and EXdi (OR 0.107, 95% CI 0.024–0.486, p=0.004) as independent predictors of weaning outcomes in ICU patients.
	Watanabe 2024, Japan [65]	**Reliability:** In healthy controls, reliability analysis of a kernel in the central diaphragm layer showed intra-observer reliability (ICC) of 0.985 and inter-observer reliability (ICC) of 0.972 for measuring DMD. **Physiological outcomes:** In healthy controls, EXdi significantly correlated with DMD (r=0.76) and strain (r= −0.61), while DMD and strain were negatively correlated (r= −0.67). No correlation was found between DMD and age, %FVC, phrenic CMAP amplitude, Tdi or TFdi. In ALS patients, DMD was greatest in the deep diaphragm layer and significantly correlated with strain (r= −0.64). Central DMD (0.6±1.4 mm) and strain (−11.0±6.2%) were significantly lower than in healthy controls. DMD correlated with phrenic CMAP amplitude (r=0.63) and respiratory rate (r= −0.55). DMD decreased in some patients despite normal %FVC, with no significant correlation found between DMD and Tdi or TFdi. **Clinical outcomes:** DMD was negatively correlated with the change in ALS Functional Rating Scale-Revised scores per month after the examination (r= −0.61, p=0.02), and patients with a larger rate of decline had significantly lower DMD (p=0.03).
**Tissue Doppler imaging**	Fayssoil 2019, France [66]	**Reliability:** N/A **Physiological outcomes:** Right sniff PCVdi showed strong correlations with FVC (r=0.72, p<0.0001) and sniff nasal pressure (r=0.66, p<0.0001). Supine FVC was also significantly associated with right diaphragm PCVdi (r=0.59, p=0.0007, n=29). This relationship was consistent across neuromuscular disorders, including Duchenne muscular dystrophy, myotonic dystrophy type 1 and other myopathies. **Clinical outcomes:** A right PCVdi cut-off of 7.5 cm·s^−1^ predicted FVC <60% with 84% sensitivity and 89% specificity, while a right EXdi cut-off of 25 mm achieved 100% sensitivity and 64% specificity. The AUC was 0.93 (p<0.0001) for sniff right EXdi and 0.86 (p<0.001) for right PCVdi. In patients with more severe respiratory impairment (FVC <30%), the right PCVdi cut-off decreased to 6.5 cm·s^−1^, with 90% sensitivity and 56% specificity, while the right EXdi cut-off of 10.5 mm provided 84% sensitivity and specificity. The AUC for right PCVdi remained high but slightly decreased to 0.76 (p<0.017).
	Soilemezi 2020, Greece [67]	**Reliability:** All variables (PCVdi, PRVdi, VTIdi, MRRdi) demonstrated excellent intra- and inter-observer reproducibility, with ICCs >0.89 for each measurement. Coefficient of variance values were consistently <10%, ranging from 2.49% to 8.81%. **Physiological outcomes:** Significant correlations were found between peak *P*di and PCVdi (R^2^=0.727, p<0.001), PTPdi and PCVdi (R^2^=0.650, p=0.007), and *P*di-MRR and PW-TDI-MRRdi (R^2^=0.634, p<0.001). A weaker correlation was noted between VTIdi and PTPdi (R^2^=0.285). **Clinical outcomes:** Healthy volunteers and patients with successful weaning had lower values for all PW-TDI parameters compared to those with weaning failure, except for VTIdi. Values were: PCVdi: 1.35±0.34 cm·s^−1^ (healthy), 1.50±0.59 cm·s^−1^ (successful weaning), 2.66±2.14 cm·s^−1^ (weaning failure) (p<0.001); PRVdi: 1.19±0.39 cm·s^−1^ (healthy), 1.53±0.73 cm·s^−1^ (successful weaning), 3.36±2.40 cm·s^−1^ (weaning failure) (p<0.001); MRRdi: 3.64±2.02 cm·s^−2^ (healthy), 10.25±5.88 cm·s^−2^ (successful weaning), 29.47±23.95 cm·s^−2^ (weaning failure) (p<0.001).
	Cammarota 2021, Italy [68]	**Reliability:** Intra-observer reliability for respiratory parameters was assessed using Pearson and ICC. Assessor 1 had high reliability for PCVdi (0.98, 95% CI 0.97–0.99), VTIdi (0.97, 95% CI 0.95–0.99) and PRVdi (0.96, 95% CI 0.94–0.98), with ICCs ranging from 0.86 to 0.98. Assessor 2 also showed strong reliability, especially for PCVdi (0.97, 95% CI 0.96–0.98) and PRVdi (0.98, 95% CI 0.98–0.99), with ICCs between 0.87 and 0.98. Inter-observer reliability was strong for PCVdi (0.97, 95% CI 0.95–0.98) and VTIdi (0.96, 95% CI 0.94–0.97), with ICCs ranging from 0.85 to 0.97. **Physiological outcomes:** N/A **Clinical outcomes:** PW-TDI evaluated across 300 breaths (237 extubation successes, 63 failures). No differences in VTIdi were found at the end of SBT. However, patients with successful extubation had significantly lower PCVdi (1.8 cm·s^−1^ *versus* 3.1 cm·s^−1^, p<0.001), MCVdi (1.1 cm·s^−1^ *versus* 1.6 cm·s^−1^, p<0.001) and inspiratory acceleration (4.2 cm·s^−2^ *versus* 8.8 cm·s^−2^, p=0.002) compared to patients with failed extubation. During expiration, patients with failed extubation showed higher PRVdi (1.8 cm·s^−1^ *versus* 2.6 cm·s^−1^, p<0.001), expiratory mean velocity (0.9 cm·s^−1^ *versus* 1.1 cm·s^−1^, p=0.002) and MRRdi (7.1 cm·s^−2^ *versus* 11.2 cm·s^−2^, p=0.004). The predictive accuracy of PW-TDI variables for extubation failure showed PCVdi with an AUC of 0.80 (p<0.001) at a cut-off of >2.2 cm·s^−1^ (sensitivity 76.2%, specificity 62.0%) and MCVdi with an AUC of 0.80 (p<0.001) at a cut-off of >1.4 cm·s^−1^ (sensitivity 71.4%, specificity 77.2%). AUC values for inspiratory velocities were significantly higher than for conventional Rapid Shallow Breathing Index (p=0.036 for PCVdi; p=0.042 for MCVdi).
	Benli 2024, Turkey [69]	**Reliability:** N/A **Physiological outcomes:** N/A **Clinical outcomes:** PCVdi measurements increased significantly more in the IMT and healthy controls groups compared to the conventional physiotherapy group (p=0.028 and p=0.015, respectively). There was a significant difference in the change in PRVdi pre- and post-intervention between the IMT and healthy controls groups (p=0.029 and p=0.020, respectively). In the conventional physiotherapy group, EXdi did not change significantly (p=0.285), while it significantly increased in the healthy controls and IMT groups post-intervention (p=0.005 for both).
	Xin 2024, China [70]	**Reliability:** N/A **Physiological outcomes:** N/A **Clinical outcomes:** MCVdi showed no significant differences between successful and failed weaning groups (p>0.05). In contrast, PCVdi, PRVdi, inspiratory acceleration and MRRdi were lower in the successful weaning group, while EXdi was significantly higher (p<0.05). For diagnostic performance, PCVdi had an AUC of 0.812 (95% CI 0.718–0.906) with a cut-off of 2.82 cm·s^−1^, sensitivity 82.1%, specificity 78.7%, PPV 89.3%, NPV 66.7%, +LR 3.85 and −LR 0.23. PRVdi demonstrated an AUC of 0.85 (95% CI 0.773–0.933) with a cut-off of 3.33 cm·s^−1^, sensitivity 92.9%, specificity 65.6%, PPV 84.8%, NPV 78.3%, +LR 2.7 and −LR 0.11. Inspiratory acceleration had an AUC of 0.74 (95% CI 0.630–0.859) with a cut-off of 4.32 cm·s^−2^, sensitivity 60.7%, specificity 83.6%, PPV 88.1%, NPV 48.9%, +LR 3.70 and −LR 0.47. MRRdi had an AUC of 0.856 (95% CI 0.781–0.936) with a cut-off of 9.25 cm·s^−2^, sensitivity 89.3%, specificity 75.4%, PPV 88.5%, NPV 75.0%, +LR 3.63 and −LR 0.14. Finally, EXdi showed an AUC of 0.567 (95% CI 0.440–0.695) with a cut-off of 1.56 cm·s^−1^, sensitivity 75.1% and specificity 47.5%.

+LR: positive likelihood ratio; −LR: negative likelihood ratio; ALS: amyotrophic lateral sclerosis; AU: acoustic unit; AUC: area under the curve; BMI: body mass index; CAT: COPD Assessment Test; CC: correlation coefficient; CI: confidence interval; CMAP: compound motor action potential; Dlcos: costal/zone of apposition diaphragm strain; Dlcru: crural diaphragm strain; Dldome: diaphragmatic dome strain; DMD: diaphragm moving distance; DOD: dome of diaphragm; ED_50_: 50th percentile of the echogenicity/echodensity greyscale; ED_85_: 85th percentile of the echogenicity/echodensity greyscale; ED_mean_: mean echogenicity/echodensity greyscale value; EXdi: diaphragm excursion; EXdi-ZOA: excursion of the zone of apposition; FEV_1_: forced expiratory volume in 1 s; FRC: functional residual capacity; FVC: forced vital capacity; HEA_65_: percentage of pixels above the echogenicity/echodensity greyscale value of 65; IC: inspiratory capacity; ICC: intra-class correlation coefficient; ICU: intensive care unit; IMT: inspiratory muscle training; IQR: interquartile range; MCVdi: diaphragm mean contraction velocity; MFR_DIA_: microvascular blood flux rate of the diaphragm; MIP: maximal inspiratory pressure; mMRC: modified Medical Research Council score; MRRdi: diaphragm maximal relaxation rate; MV: mechanical ventilation; N/A: not available; NPV: negative predictive value; OR: odds ratio; PCVdi: diaphragm peak contraction velocity; *P*di: transdiaphragmatic pressure; PPC: postoperative pulmonary complications; PPV: positive predictive value; PRVdi: diaphragm peak relaxation velocity; PS: pressure support; PSV: pressure support ventilation; PTPdi: pressure–time product of the diaphragm; PW-TDI: pulsed-wave tissue Doppler imaging; *Q̇*_DIA_: diaphragm blood flow; ROC: receiver operating characteristic; RV: residual volume; SBT: spontaneous breathing trial; SM: shear modulus; SMdi: diaphragm shear modulus; ST: speckle tracking; SWVdi: diaphragm shear wave velocity; Tdi: diaphragm thickness; TFdi: diaphragm thickening fraction; TID: transverse inspiratory deformation; TLC: total lung capacity; VTIdi: diaphragm velocity-time integral; VC_DIA_: vascular conductance of the diaphragm; ZOA: zone of apposition.

#### The area method

The area method allows the calculation of the area change (in cm^2^) and intends to estimate diaphragm movement in two dimensions: cranio-caudal and posterior-anterior [35, 37]. Image frames showing the diaphragm's position at end-inspiration and at end-expiration are identified. The entire visible portion of the diaphragm is then traced using the ultrasound machine's area-calculation function. The change in intrathoracic area is determined by subtracting the area at minimal contraction from the area at maximal contraction: Δintrathoracic area (*i.e.* area change)=area at end-inspiration−area at end-expiration (supplementary material 3, figure E1). The main results of the included studies [35–37, 71–73] are summarised in table 2.

In a preliminary study involving healthy individuals, the area method demonstrated high inter-rater reliability and good feasibility among novice operators. All were able to assess both the right and left hemidiaphragms [35], which is notable given the typical challenges associated with visualising the left side using a subcostal approach. In the same study, the area method showed a strong correlation with expired lung volume; however, this association weakened as lung volumes increased [35]. When compared to fluoroscopy, often considered a reference method for diaphragm excursion, the area method showed only moderate to weak correlations for both hemidiaphragms, with performance declining further during sniff manoeuvres [37]. These findings suggest that the area method is particularly sensitive to variations in breathing patterns, and performs best during calm, near-tidal breathing. Also, the correlation between the area method and conventional EXdi before and after thoracentesis was found to be very poor, reinforcing the idea that these two modalities may capture different aspects of diaphragmatic motion. Clinically, the area method has been mainly studied in patients undergoing thoracentesis. It successfully detected significant improvement in diaphragmatic movement following the procedure [71], yet it failed to predict symptomatic response to thoracic drainage, similar to conventional EXdi [72]. For the detection of non-expandable lung post-thoracentesis (based on both radiological findings and clinical signs), the diagnostic accuracy of the area method was limited, performing worse than M-mode and B-mode assessments, and only surpassed elastography assessment of the pleura and effusion [36]. Building on these findings, it is now important to assess the area method in other patient populations. Broader validation could help define its role across a range of respiratory conditions.

#### Contrast-enhanced ultrasound

The application of CEUS for assessing diaphragm function was explored recently (supplementary material 3, figure E2) [38]. CEUS allows real-time analysis of the wash-in and wash-out phases of ultrasound contrast agents, which consist of gas microbubbles. This method provides insights into the vascular architecture of various structures over time, and requires nonlinear imaging modes and low mechanical index settings to preserve microbubble integrity. For further technical details, refer to sources [84, 85].

The main results of the included study [38] are summarised in table 2. DUS metrics for assessing diaphragm perfusion included microvascular blood volume, microvascular blood flux rate, diaphragm blood flow (*Q̇*_DIA_) and vascular conductance (VC_DIA_). In this physiological study, Bird *et al*. [38] evaluated diaphragm perfusion in healthy individuals under varying inspiratory loads. They reported excellent test–retest and inter-analyser reliability for *Q̇*_DIA_ measurements. *Q̇*_DIA_ also increased significantly with each level of inspiratory loading, and higher transdiaphragmatic pressures (*P*di) were associated with greater *Q̇*_DIA_ and VC_DIA_. In particular, >94% of the variance in *Q̇*_DIA_ and VC_DIA_ was attributable to diaphragm pressure generation. These results were consistent across sessions, underscoring the robustness of the findings. However, technical challenges currently prevent the broader clinical application of CEUS, particularly the requirement for sustained end expiratory apnoea, and the need for further preclinical validation.

#### Echogenicity/echodensity

Echogenicity (or echodensity) reflects the sonographic properties of muscle and can be evaluated through greyscale analysis of a predefined region of interest using image-processing software, which generates histograms for muscle quality evaluation. Skeletal muscle typically appears darker due to a lower fibrous tissue content but ageing and disease can increase brightness due to fat and fibrous tissue accumulation [86–88]. The main results of the included studies [39–44] are summarised in table 2.

Two methods exist for region of interest selection: the trace and the square methods (supplementary material 3, figure E3). Four studies used the trace method [40, 41, 43, 44], while one used the square method [42] and the remaining study used both methods [39]. Five studies reported on the reliability of diaphragm echogenicity measurements, with one study focusing exclusively on this topic [39]. Multiple reports demonstrated strong intra- and inter-observer reliability for diaphragm echogenicity assessments [39, 41, 44]. One study reported a slight difference between the square and trace methods but no inter-rater discrepancies, while novice raters showed greater variability [39].

In general, diaphragm echogenicity may serve as a surrogate marker of tissue quality, because it appears to reflect underlying physiological changes; however, its determinants are not yet fully understood. Nonetheless, diaphragm echogenicity tends to increase with age, as shown by a regression model in healthy individuals [44]. Among critically ill patients, increased diaphragm echogenicity correlated with diaphragm thickness, intercostal muscle echogenicity and cumulative fluid balance, although the strength and consistency of the latter association remain variable across studies [40–42]. Mechanically ventilated patients tended to present with higher baseline echogenicity compared to healthy controls, and intensive care unit non-survivors exhibited greater increases in echogenicity over time, as well as fewer ventilator-free days when echogenicity rises [40, 41]. In surgical patients, one study found that increased diaphragm echogenicity was independently associated with the development of postoperative pulmonary complications, although its predictive performance was modest [43].

#### Excursion of the zone of apposition

A recent study introduced a novel method for estimating EXdi by visualising the zone of apposition (ZOA), as an alternative to the conventional M-mode EXdi assessment with a subcostal view [45]. This method involves marking the most cephalad points at end-inspiration and end-expiration on the skin, with the distance between these marks measured in millimetres to determine diaphragm excursion (supplementary material 3, figure E4). The main results of the included study [45] are summarised in table 2. Despite its simplicity, EXdi-ZOA achieved 100% bilateral visualisation, surpassing the conventional technique, which reached 98.7% on the right side but only 34.7% on the left [45]; the correlation between EXdi-ZOA and the conventional EXdi was weak on the right and moderate on the left. Finally, and as expected, no association was found between these assessments and TFdi, because they evaluate distinct dimensions of diaphragm function.

#### Shear-wave elastography/strain elastography

The functional mechanical properties of the diaphragm have been evaluated through elastography in 12 studies, with 11 using SWE [46, 47, 49–56, 74] and one employing SE [48]. Both techniques provide insights into tissue stiffness, a new but important aspect of diaphragm function. They share three common phases: application of excitation (stress), measurement of tissue response (strain) and estimation of mechanical parameters [89]. SWE measures the propagation of mechanical waves, while SE is a quasi-static method requiring static compression, making it operator-dependent [90]. SWE results are derived from shear wave velocity (SWV) to estimate shear modulus (SM) (supplementary material 3, figure E5), while SE provides strain and strain ratio without specific quantification. Comprehensive information is available elsewhere [89–91]. The main results of the included studies are summarised in table 2.

Studies evaluating the reliability of SWE or SE consistently reported high intra-class correlation coefficients (ICCs) for both intra- and inter-observer reliability, indicating good to excellent consistency [46–49, 52–55]. In healthy subjects under controlled laboratory conditions, intra-subject end-inspiratory diaphragmatic shear modulus (SMdi) showed a coefficient of variation ranging from 3.7% to 16.2% across different isovolumetric inspiratory efforts [46, 47]. Interestingly, one study examined the inter-operator reproducibility of end-expiratory SMdi with different probe positions, finding greater reliability in the longitudinal view (with the probe parallel to the fibres) compared to the transverse view [49]. This highlights an important consideration when assessing the diaphragm *via* SWE. The sole study on SE reported ICCs >0.90 for elastographic strain ratio measurements [48].

Several studies have examined the relationship between SWE parameters and other respiratory assessments [46, 47, 50, 52–56]. In healthy individuals, strong correlations have been observed between inspiratory mouth pressure and the SMdi ratio (end-inspiration to end-expiration), with 94% of the variation in one variable explained by the other [46]. Similarly, a strong relationship was found between mean *P*di and mean ΔSMdi (*i.e.* SMdi during inspiratory effort minus SMdi at functional residual capacity (FRC)) [47]. Both studies suggest that SWE parameters may offer valuable insights into inspiratory effort [46, 47]. In critically ill patients, changes in transdiaphragmatic pressure (Δ*P*di) showed only a moderate correlation with ΔSMdi overall, with limited intra-individual consistency [50]. This relationship appeared to be influenced by the presence of mechanical ventilation (MV) support and elevated respiratory rates. Nevertheless, ΔSMdi significantly increased during spontaneous breathing trials compared to different levels of pressure support ventilation [50], supporting the concept that higher ΔSMdi values correspond to greater diaphragmatic activation. Over time, basal SMdi fluctuated in intensive care unit (ICU) patients, decreasing in 41% and increasing in 51%, with reductions more frequently observed in older or septic patients, and in those with increased diaphragm thickness or prolonged exposure to controlled MV [51].

In populations with chronic disease such as COPD, various diaphragm SWE-derived parameters have been found to significantly correlate with pulmonary function measures, including forced expiratory volume in 1 s (FEV_1_), forced vital capacity (FVC) and FEV_1_/FVC ratios [52, 53, 55]. Notably, higher diaphragm stiffness is observed in COPD patients with more severe disease and a greater symptom burden [52, 55]. While diaphragm stiffness generally increases with disease severity, some studies report no significant differences between the severe and very severe stages of COPD [52, 53]. %SMdi (the percentage change in diaphragm stiffness from end-expiration to end-inspiration) has been shown to negatively correlate with TFdi, with a notably strong association observed in patients with a higher symptom burden [55]. These results suggest that diaphragm SWE could serve as a valuable biomarker for evaluating both functional and clinical status in COPD patients. However, the exact relationship between these measurements and lung function, particularly in conditions involving lung hyperinflation, remains unclear, because we cannot rule out some influence of the diaphragm's “resting position” on the measurements. Finally, in healthy individuals, no significant differences in SMdi were found across various age groups or body mass index categories [56].

#### Speckle tracking

Initially developed for heart assessment [92], ST echocardiography evaluates cardiac function by tracking stable myocardial speckles generated through ultrasound interactions with myocardial tissue [93]. This technique provides myocardial deformation, known as strain, by analysing the distances and displacements of these speckles throughout the cardiac cycle. ST is particularly effective for Lagrangian strain analysis, which measures deformation relative to the original length, distinguishing between normal strains (perpendicular to the surface) and shear strains (tangential) [94]. In diaphragm assessment, ST has been adapted to evaluate diaphragmatic deformation and strain (supplementary material 3, figure E6) [57–59, 61–65], also offering insights into cranio-caudal displacement at the ZOA [58, 65] and overall movement similar to EXdi [60]. The main results of the included studies are summarised in table 2.

Four studies assessed the reliability of different ST modalities, consistently reporting high intra- and inter-observer ICCs (≥0.87) in healthy individuals and patients on MV [59, 63, 65]. A small study also demonstrated ST's superiority over conventional EXdi in visualising the left hemidiaphragm during normal and deep breathing [60].

ST effectively evaluated diaphragm contractility under inspiratory threshold loading conditions, with both longitudinal strain and strain rate correlating strongly with *P*di and diaphragm electrical activity [61]. However, its relationship with TFdi (another DUS-derived estimate of inspiratory effort) remains inconsistent [58, 59, 61, 63]. Interestingly, strain assessment using a subcostal approach enabled partition-independent analysis, revealing significant changes, characterised by a shift towards more negative values, specifically in the crural and costal portions, but not in the dome portion, when passing from tidal to deep breathing [57]. Another study investigated the possibility of quantifying the diaphragm moving distance (DMD) at the ZOA by following a kernel (a square grid measuring 5 pixels) placed in the central layer of the diaphragm. The authors found significant correlations with conventional EXdi and negative correlations with strain in healthy individuals. In patients with amyotrophic lateral sclerosis (ALS), DMD and strain values were significantly lower than in healthy controls [65].

As for other DUS techniques, ST has been used to predict outcomes. In weaning trials, lower strain values were linked to success, with a threshold below −21% predicting successful extubation with high sensitivity but moderate specificity [63]. When focusing on the diaphragmatic portions, a strain value greater than −9.8% in the crural region showed a “fair” ability to predict weaning success, an accuracy that improved to “good” when combined with conventional EXdi measurements [64]. Beyond the acute/ICU context, lower DMD values in patients with ALS negatively correlated with greater functional decline, assessed using the ALS Functional Rating Scale-Revised score [65].

#### Pulsed-wave tissue Doppler imaging

PW-TDI is an ultrasound-based technique that emerged from cardiology for assessing left ventricular motion [95]. It uses the PW Doppler principle to evaluate cardiac tissue motion, focusing on low-velocity, high-amplitude signals by employing a high-pass filter [96, 97]. PW-TDI applied to the diaphragm yields various motion metrics found in the literature, such as peak contraction velocity (PCVdi), the maximal diaphragmatic velocity during inspiration, in cm·s^−1^; velocity-time integral (VTIdi), the area under the velocity curve for the entire inspiration phase, in cm; mean contraction velocity (MCVdi), the average velocity computed over the entire inspiratory phase, in cm·s^−1^; peak relaxation velocity (PRVdi), the maximal diaphragmatic velocity during expiration, in cm·s^−1^; and maximal relaxation rate (MRRdi), the slope of the steepest initial portion of the diaphragmatic motion velocity curve, in cm·s^−2^. Several of these elements and others are illustrated in supplementary material 3, figure E7. The main results of the included studies [66–70] are summarised in table 2.

Available studies confirm PW-TDI's strong reliability, with intra- and inter-observer ICCs exceeding 0.89 in both healthy individuals and patients on MV [67, 68]. In critically ill patients, PCVdi demonstrated a strong correlation with both *P*di and the diaphragm pressure–time product (PTPdi), whereas VTIdi showed a weaker association with PTPdi [67]. In a population of neuromuscular patients, PCVdi presented a strong association with FVC and sniff nasal pressure but did not outperform right conventional EXdi in predicting severe FVC reductions, *i.e.* below 60% and 30% of the predicted value [66].

In the context of MV, successful weaning was associated with significantly reduced values for PW-TDI parameters compared to the failure group [67, 68, 70], with PCVdi (>2.2 cm·s^−1^) and MCVdi (>1.4 cm·s^−1^) outperforming traditional indices like the Rapid Shallow Breathing Index [68]. The predictive value of PW-TDI was further confirmed in critically ill patients [70]. Additionally, a small study demonstrated PW-TDI's responsiveness to inspiratory muscle training, with significant post-intervention improvements in PCVdi and PRVdi [69].

## Discussion

**FIGURE 2:**
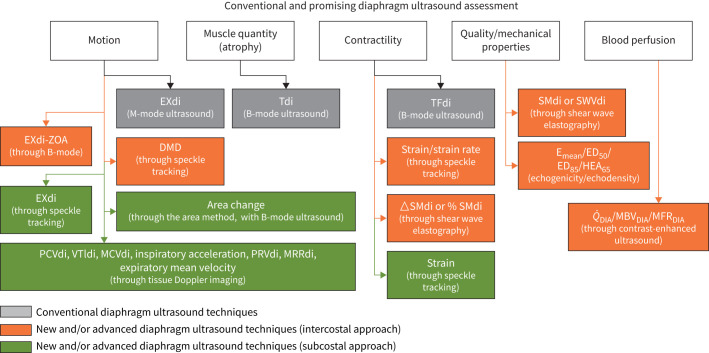
Schematic representation of conventional ultrasound techniques and the potential role of new and/or advanced techniques, identified through this literature review, in the multidimensional assessment of diaphragm structure and function. DMD: diaphragm moving distance; ED_50_: 50th percentile of the echogenicity/echodensity greyscale; ED_85_: 85th percentile of the echogenicity/echodensity greyscale; ED_mean_: mean echogenicity/echodensity greyscale value; EXdi: diaphragm excursion; EXdi-ZOA: excursion of the zone of apposition; HEA_65_: percentage of pixels above the echogenicity/echodensity greyscale value of 65; MBV_DIA_: microvascular blood volume of the diaphragm; MCVdi: diaphragm mean contraction velocity; MFR_DIA_: microvascular blood flux rate of the diaphragm; MRRdi: diaphragm maximal relaxation rate; PCVdi: diaphragm peak contraction velocity; PRVdi: diaphragm peak relaxation velocity; *Q̇*_DIA_: diaphragm blood flow; SMdi: diaphragm shear modulus; ΔSMdi: difference between end-inspiratory and end-expiratory diaphragm shear modulus; %SMdi: percentage change of diaphragm shear modulus from end-expiration to end-inspiration; SWVdi: diaphragm shear wave velocity; Tdi: diaphragm thickness; TFdi: diaphragm thickening fraction; VTIdi: diaphragm velocity-time integral.

This review aimed to identify new and/or advanced ultrasound techniques for assessing diaphragm structure and function in adults. We were able to describe seven innovative techniques. In figure 2, the ultrasound markers derived from these novel techniques were categorised based on the hypothetical information they provide for both established and newly recognised dimensions of DUS. New ultrasound markers were incorporated into the already covered dimensions, *i.e.* motion and contractility, from conventional ultrasound techniques. Additionally, we identified two new dimensions: quality/functional mechanical properties and blood flow assessment of the diaphragm. However, in terms of muscle quantity (primarily related to atrophy), assessment remains limited to Tdi.

Ultrasound assessment of diaphragm motion has traditionally relied on EXdi-related M-mode measurements from a subcostal window. While this method is popular for its simplicity and clinical relevance, it has some limitations. Key challenges include difficulties in visualising the left hemidiaphragm due to an insufficient acoustic window created by gastric and intestinal gas, mostly during maximal breathing [98], and the one-dimensional nature of the technique, which restricts the analysis to a single plane. Recent advancements in ultrasound methodologies have sought to overcome these limitations by providing more comprehensive assessments of diaphragmatic motion.

The area method represents one such innovation, grounded in the hypothesis that it can assess diaphragm movement in two dimensions [35]. Despite demonstrating feasibility in selected contexts, such as thoracentesis procedures, its broader clinical performance is still under evaluation. ST also shows potential for overcoming some of the limitations of excursion-based methods, especially in challenging conditions such as left hemidiaphragm visualisation. While early studies have demonstrated its feasibility [60], the technique is still in the developmental phase, requiring further validation to establish its diagnostic value and clinical utility.

Beyond traditional subcostal views, the EXdi-ZOA technique offers a simple lateral approach to quantify diaphragm excursion across both hemidiaphragms [45]. Separately, the DMD, derived from ST, enables detailed analysis of displacement at the ZOA [65]. While both methods provide region-specific insights, their added clinical value relative to conventional excursion has yet to be fully determined.

Among the emerging methods for assessing diaphragm motion, PW-TDI provides a detailed appraisal of contractile kinematics of the diaphragm, including both inspiratory and expiratory phases. Unlike traditional displacement-based methods, PW-TDI provides time-resolved measurements that reflect the dynamics of muscle effort and timing, parameters that may hold greater clinical relevance, particularly in settings such as ventilator weaning. Among these, contraction velocities have shown stronger alignment with invasive estimates of respiratory effort than conventional indices [67, 68, 70], underscoring their potential diagnostic value. Nevertheless, diaphragm displacement alone may not reliably reflect inspiratory effort [99, 100], which may explain the limited discriminative power observed with excursion-based PW-TDI parameters such as VTIdi [67, 68]. Although technically dependent on ultrasound settings and probe positioning, PW-TDI demonstrates strong reliability, supporting its potential value as a clinical monitoring tool.

The assessment of diaphragm quality and functional mechanical properties through ultrasound offers valuable insights into tissue stiffness and structural integrity, which are often missed by visual inspection or traditional methods. Echogenicity analysis, a reliable indicator of muscle quality, often shows increased echogenicity in conditions involving fat or fibrous tissue infiltration, common in neuromuscular diseases [101, 102]. It can serve as a sensitive marker for tracking diaphragm dysfunction, as seen in other structures like the rectus femoris, where increased echogenicity indicated muscle fibre necrosis in critically ill patients [103].

Ultrasound-based assessment of diaphragm stiffness using elastography provides insights into tissue quality and mechanical integrity that conventional techniques cannot capture. SWE-derived metrics, such as SMdi, decrease during controlled MV compared to assisted modes, suggesting disuse-related alterations in diaphragm properties [51]. While SMdi inversely correlates with end-expiratory thickness [51], this relationship remains complex and is likely influenced by confounders such as end-expiratory lung volume or baseline muscle tone. These nuances are particularly relevant in critically ill populations, where different stressors may elicit heterogeneous responses in muscle tissue, potentially reflected by distinct stiffness patterns. This rationale also applies to other conditions with altered muscle tone. Both SWE and SE have demonstrated good intra- and inter-observer reliability [48, 49, 52, 55]; however, SWE's performance may be limited in patients with elevated respiratory rates due to technical constraints [50]. SE remains less commonly used, primarily because its non-uniform stress distribution precludes quantitative stiffness estimation, though it may still offer useful indirect information [90].

Diaphragmatic contractility is essential for respiratory function, yet its gold-standard measurement, *P*di, relies on invasive techniques that limit its routine use. Consequently, noninvasive alternatives have emerged, among them ultrasound-based markers such as elastography-derived ΔSMdi and ST strain measurements. These indices show promising associations with physiological markers of diaphragmatic activation, including *P*di and diaphragm electrical activity [47, 61], and may offer a more nuanced understanding of contractile function than conventional thickening indices. In addition, ST offers additional advantages owing to its angle independence and its ability to assess diaphragmatic deformation from both intercostal and subcostal views, enhancing its clinical applicability [57, 64]. Strain-based ST markers have shown predictive value in ICU settings, with lower values associated with successful weaning [63, 64].

Blood flow and oxygenation play a vital role in skeletal muscle functioning. Blood flow appears to be regulated by a competitive relationship between respiratory and peripheral muscles, shifting based on the work of breathing [104]. In critically ill patients, skeletal muscle microvascular blood flow and oxygen transport can be impaired shortly after ICU admission [105]. Other studies showed that peripheral tissue oxygenation is an important predictor of mortality, especially in patients with sepsis and septic shock [106–108]. It is therefore plausible that respiratory muscles, particularly the diaphragm, may be similarly affected [109, 110]. In animal models, MV at both low and high positive end-expiratory pressure has been shown to reduce diaphragm blood flow [111], and prolonged MV further diminishes the diaphragm's ability to increase blood flow in response to contractile activity [112]. The recent introduction of CEUS provides a noninvasive method for quantifying diaphragmatic perfusion [38], opening new avenues for its study in clinical practice.

Taken together, the findings of this review suggest that these techniques should not be viewed as competing alternatives but rather as complementary tools, each offering insights into different dimensions of diaphragm structure or function (figure 2). No single technique emerges as clearly superior; instead, they provide nonredundant, multidimensional information that could enhance patient assessment if integrated thoughtfully.

This multidimensionality, however, also poses challenges for clinical translation. Variability in protocols, patient populations and outcome definitions limits cross-study comparability. Several techniques remain confined to exploratory or pilot-phase research. Moreover, the lack of standardised thresholds, acquisition protocols and reference values complicates broader implementation. Despite these limitations, several techniques have shown encouraging associations with physiological parameters, clinical outcomes and prognostic events such as weaning failure or surgical complications. To move toward routine application, future research should prioritise unified protocols and large-scale validation.

Finally, as ultrasound technology continues to evolve, its clinical potential will likely be amplified by the rise of artificial intelligence. Developments in automation, machine learning and deep learning are expected to enhance image interpretation, reproducibility and real-time feedback. While not covered in this review, these innovations represent the next horizon in diaphragm imaging. In the meantime, a strong understanding of diaphragm physiology remains essential for integrating these tools into clinical decision-making.

### Strengths and limitations of this review

This integrative review offers a timely and comprehensive synthesis of emerging ultrasound techniques for diaphragm assessment, encompassing both structural and functional aspects. A key strength of this work lies in its transparent and methodologically sound approach. We applied clearly defined eligibility criteria, a focused and systematic search strategy (including backwards and forwards citation tracking), and a structured process for study selection and data extraction, the latter performed by two independent reviewers. All extracted data are presented in a comprehensive supplementary file, enhancing transparency. While not a formal systematic review, several structural principles from the Preferred Reporting Items for Systematic Reviews and Meta-Analyses (PRISMA) guidelines were respected.

Another strength is the focus on novel and evolving techniques, many of which have only recently been applied to diaphragm imaging and remain in early stages of clinical validation. By mapping current evidence on their reliability and potential clinical applications, this review offers a valuable resource for both researchers and clinicians and helps identify priorities for future research.

Several limitations should nonetheless be acknowledged. First, although we aligned with several elements of PRISMA, this review does not meet all the criteria of a full systematic review. For example, we did not search across multiple databases or conduct a qualitative appraisal of the included studies. As such, the review was not registered in PROSPERO or another protocol registry. These omissions may affect the comprehensiveness and reproducibility typically expected of systematic reviews. Second, there was notable heterogeneity among the included studies in terms of populations, ultrasound protocols and outcome measures, limiting comparability and precluding quantitative synthesis. Finally, because many of the techniques reviewed are still in exploratory stages, their clinical utility remains promising but not yet established in routine practice.

## Conclusion

Innovative ultrasound techniques for assessing diaphragm structure and function have emerged over the past decade, offering new opportunities for a more comprehensive, multidimensional evaluation. Beyond motion, thickness and contractility, recent advancements now allow for the assessment of muscle quality, functional mechanical properties and blood flow. Additionally, some of these techniques may offer viable alternatives when conventional methods are not fully applicable or interpretable.

Nevertheless, further research is required to refine and standardise imaging protocols, validate these techniques against gold-standard measures and assess their utility across diverse clinical scenarios. Establishing standardised ultrasound-based assessments will be key to their broader clinical adoption. As these methods continue to evolve, their integration into routine practice has the potential to improve patient outcomes by providing physiologically relevant, accessible and dynamic evaluations of diaphragm function.

**Points for clinical practice**

- It is essential to recognise that no single ultrasound technique can provide a complete picture of diaphragm function. Clinicians should consider using a combination of different techniques to capture a more comprehensive concordant assessment of diaphragm structure and function.
- New ultrasound techniques may be promising and viable alternatives to conventional methods, particularly in cases where clinical context or patient-specific factors limit the applicability of traditional assessments.

**Questions for future research**

- Future research in the field should continue to focus on investigating new ultrasound techniques in larger studies, comparing their diagnostic capabilities against well-established assessments of diaphragmatic structure and function. This will help to better determine their diagnostic accuracy and reliability in diverse clinical populations.
- The creation of integrated multidimensional ultrasound-based models has a strong rationale and can support prognostic evaluations while improving the monitoring of diaphragm-related disease/dysfunction progression. Gaining insight into how these advanced assessments correlate with patient prognosis and treatment effectiveness will be essential for their integration into standard clinical practice.

## Supplementary material

**Please note:** supplementary material is not edited by the Editorial Office, and is uploaded as it has been supplied by the author. Supplementary material 1: Literature search strategy ERR-0089-2025.SUPPLEMENT1 Supplementary material 2: Characteristics of included studies ERR-0089-2025.SUPPLEMENT2 Supplementary material 3: Illustration of ultrasound techniques ERR-0089-2025.SUPPLEMENT3

## References

[1] McCool FD, Tzelepis GE. Dysfunction of the diaphragm. N Engl J Med 2012; 366: 932–942. doi:10.1056/NEJMra1007236 22397655

[2] Troyer AD, Wilson TA. Action of the diaphragm on the rib cage. J Appl Physiol (1985) 2016; 121: 391–400. doi:10.1152/japplphysiol.00268.2016 27283911

[3] Hodges PW, Gandevia SC. Activation of the human diaphragm during a repetitive postural task. J Physiol 2000; 522: 165–175. doi:10.1111/j.1469-7793.2000.t01-1-00165.xm 10618161 PMC2269747

[4] Hodges PW, Butler JE, McKenzie DK, et al. Contraction of the human diaphragm during rapid postural adjustments. J Physiol 1997; 505: 539–548. doi:10.1111/j.1469-7793.1997.539bb.x 9423192 PMC1160083

[5] Janssens L, Brumagne S, McConnell AK, et al. Greater diaphragm fatigability in individuals with recurrent low back pain. Respir Physiol Neurobiol 2013; 188: 119–123. doi:10.1016/j.resp.2013.05.028 23727158

[6] Beeckmans N, Vermeersch A, Lysens R, et al. The presence of respiratory disorders in individuals with low back pain: a systematic review. Man Ther 2016; 26: 77–86. doi:10.1016/j.math.2016.07.011 27501326

[7] Smith MD, Russell A, Hodges PW. The relationship between incontinence, breathing disorders, gastrointestinal symptoms, and back pain in women: a longitudinal cohort study. Clin J Pain 2014; 30: 162–167. doi:10.1097/AJP.0b013e31828b10fe 23486234

[8] Smith MD, Russell A, Hodges PW. Disorders of breathing and continence have a stronger association with back pain than obesity and physical activity. Aust J Physiother 2006; 52: 11–16. doi:10.1016/S0004-9514(06)70057-5 16515418

[9] Hodges PW, Gandevia SC. Changes in intra-abdominal pressure during postural and respiratory activation of the human diaphragm. J Appl Physiol (1985) 2000; 89: 967–976. doi:10.1152/jappl.2000.89.3.967 10956340

[10] Levine S, Nguyen T, Taylor N, et al. Rapid disuse atrophy of diaphragm fibers in mechanically ventilated humans. N Engl J Med 2008; 358: 1327–1335. doi:10.1056/NEJMoa070447 18367735

[11] Jaber S, Petrof BJ, Jung B, et al. Rapidly progressive diaphragmatic weakness and injury during mechanical ventilation in humans. Am J Respir Crit Care Med 2011; 183: 364–371. doi:10.1164/rccm.201004-0670OC 20813887

[12] van den Berg M, Shi Z, Claassen WJ, et al. Super-relaxed myosins contribute to respiratory muscle hibernation in mechanically ventilated patients. Sci Transl Med 2024; 16: eadg3894. doi:10.1126/scitranslmed.adg3894 39083588 PMC11586073

[13] Goligher EC, Dres M, Fan E, et al. Mechanical ventilation-induced diaphragm atrophy strongly impacts clinical outcomes. Am J Respir Crit Care Med 2018; 197: 204–213. doi:10.1164/rccm.201703-0536OC 28930478

[14] Barreiro E, Gea J. Respiratory and limb muscle dysfunction in COPD. COPD 2015; 12: 413–426. doi:10.3109/15412555.2014.974737 25438125

[15] Antenora F, Fantini R, Iattoni A, et al. Prevalence and outcomes of diaphragmatic dysfunction assessed by ultrasound technology during acute exacerbation of COPD: a pilot study. Respirology 2017; 22: 338–344. doi:10.1111/resp.12916 27743430

[16] Levine S, Bashir MH, Clanton TL, et al. COPD elicits remodeling of the diaphragm and vastus lateralis muscles in humans. J Appl Physiol (1985) 2013; 114: 1235–1245. doi:10.1152/japplphysiol.01121.2012 23264538 PMC3656432

[17] Vilaro J, Ramirez-Sarmiento A, Martinez-Llorens JM, et al. Global muscle dysfunction as a risk factor of readmission to hospital due to COPD exacerbations. Respir Med 2010; 104: 1896–1902. doi:10.1016/j.rmed.2010.05.001 20541383

[18] Gayan-Ramirez G, Decramer M. Mechanisms of striated muscle dysfunction during acute exacerbations of COPD. J Appl Physiol (1985) 2013; 114: 1291–1299. doi:10.1152/japplphysiol.00847.2012 23372146

[19] Ambrosino N, Carpene N, Gherardi M. Chronic respiratory care for neuromuscular diseases in adults. Eur Respir J 2009; 34: 444–451. doi:10.1183/09031936.00182208 19648521

[20] Poddighe D, Van Hollebeke M, Rodrigues A, et al. Respiratory muscle dysfunction in acute and chronic respiratory failure: how to diagnose and how to treat? Eur Respir Rev 2024; 33: 240150. doi:10.1183/16000617.0150-2024 39631928 PMC11615664

[21] Bourke SC. Respiratory involvement in neuromuscular disease. Clin Med (Lond) 2014; 14: 72–75. doi:10.7861/clinmedicine.14-1-72 24532751 PMC5873628

[22] Cabrera Serrano M, Rabinstein AA. Causes and outcomes of acute neuromuscular respiratory failure. Arch Neurol 2010; 67: 1089–1094. doi:10.1001/archneurol.2010.207 20837853

[23] Laveneziana P, Albuquerque A, Aliverti A, et al. ERS statement on respiratory muscle testing at rest and during exercise. Eur Respir J 2019; 53: 1801214. doi: 10.1183/13993003.01214-2018 30956204

[24] Hermans G, Demoule A, Heunks L. How I perform diaphragmatic ultrasound in the intensive care unit. Intensive Care Med 2024; 50: 2175–2178. doi:10.1007/s00134-024-07688-x 39470799

[25] Demi L, Wolfram F, Klersy C, et al. New international guidelines and consensus on the use of lung ultrasound. J Ultrasound Med 2023; 42: 309–344. doi:10.1002/jum.16088 35993596 PMC10086956

[26] Haaksma ME, Smit JM, Boussuges A, et al. EXpert consensus on diaphragm ultrasonography in the critically ill (EXODUS): a Delphi consensus statement on the measurement of diaphragm ultrasound-derived parameters in a critical care setting. Crit Care 2022; 26: 99. doi:10.1186/s13054-022-03975-5 35395861 PMC8991486

[27] Laursen CB, Clive A, Hallifax R, et al. European Respiratory Society statement on thoracic ultrasound. Eur Respir J 2021; 57: 2001519. doi:10.1183/13993003.01519-2020 33033148

[28] Tuinman PR, Jonkman AH, Dres M, et al. Respiratory muscle ultrasonography: methodology, basic and advanced principles and clinical applications in ICU and ED patients – a narrative review. Intensive Care Med 2020; 46: 594–605. doi:10.1007/s00134-019-05892-8 31938825 PMC7103016

[29] Ferrari G, Skaarup SH, Panero F, et al. The diaphragm. *In:* Laursen CB, Rahman NM, Volpicelli G, eds. Thoracic Ultrasound (ERS Monograph). Sheffield, European Respiratory Society, 2018; pp. 129–147.

[30] Volpicelli G, Elbarbary M, Blaivas M, et al. International evidence-based recommendations for point-of-care lung ultrasound. Intensive Care Med 2012; 38: 577–591. doi:10.1007/s00134-012-2513-4 22392031

[31] Goligher EC, Brochard LJ, Reid WD, et al. Diaphragmatic myotrauma: a mediator of prolonged ventilation and poor patient outcomes in acute respiratory failure. Lancet Respir Med 2019; 7: 90–98. doi:10.1016/S2213-2600(18)30366-7 30455078

[32] Wagner MW, Namdar K, Biswas A, et al. Radiomics, machine learning, and artificial intelligence-what the neuroradiologist needs to know. Neuroradiology 2021; 63: 1957–1967. doi:10.1007/s00234-021-02813-9 34537858 PMC8449698

[33] Haddaway NR, Grainger MJ, Gray CT. citationchaser: an R package for forward and backward citations chasing in academic searching. Res Synth Methods 2022; 13: 533–545. doi:10.1002/jrsm.1563 35472127

[34] Ouzzani M, Hammady H, Fedorowicz Z, et al. Rayyan: a web and mobile app for systematic reviews. Syst Rev 2016; 5: 210. doi:10.1186/s13643-016-0384-4 27919275 PMC5139140

[35] Skaarup SH, Lokke A, Laursen CB. The Area method: a new method for ultrasound assessment of diaphragmatic movement. Crit Ultrasound J 2018; 10: 15. doi:10.1186/s13089-018-0092-5 29946769 PMC6019663

[36] Petersen JK, Fjaellegaard K, Rasmussen DB, et al. Ultrasound in the diagnosis of non-expandable lung: a prospective observational study of M-mode, B-mode, and 2D-shear wave elastography. Diagnostics (Basel) 2024; 14: 204. doi:10.3390/diagnostics14020204 38248080 PMC10813923

[37] Skaarup SH, Juhl-Olsen P, Grundahl AS, et al. Replacement of fluoroscopy by ultrasonography in the evaluation of hemidiaphragm function, an exploratory prospective study. Ultrasound J 2024; 16: 1. doi:10.1186/s13089-023-00355-0 38189895 PMC10774234

[38] Bird JD, Lance ML, Banser TRW, et al. Quantifying diaphragm blood flow with contrast-enhanced ultrasound in humans. Chest 2024; 166: 821–834. doi:10.1016/j.chest.2024.04.026 38821183 PMC11492223

[39] Sarwal A, Parry SM, Berry MJ, et al. Interobserver reliability of quantitative muscle sonographic analysis in the critically ill population. J Ultrasound Med 2015; 34: 1191–1200. doi:10.7863/ultra.34.7.1191 26112621

[40] Coiffard B, Riegler S, Sklar MC, et al. Diaphragm echodensity in mechanically ventilated patients: a description of technique and outcomes. Crit Care 2021; 25: 64. doi:10.1186/s13054-021-03494-9 33593412 PMC7884870

[41] Umbrello M, Guglielmetti L, Formenti P, et al. Qualitative and quantitative muscle ultrasound changes in patients with COVID-19-related ARDS. Nutrition 2021; 91–92: 111449. doi:10.1016/j.nut.2021.111449 PMC8364677 34583135

[42] Formenti P, Umbrello M, Castagna V, et al. Respiratory and peripheral muscular ultrasound characteristics in ICU COVID-19 ARDS patients. J Crit Care 2022; 67: 14–20. doi:10.1016/j.jcrc.2021.09.007 34600218 PMC8480969

[43] Fu X, Wang Z, Wang L, et al. Increased diaphragm echodensity correlates with postoperative pulmonary complications in patients after major abdominal surgery: a prospective observational study. BMC Pulm Med 2022; 22: 400. doi:10.1186/s12890-022-02194-6 36333695 PMC9636692

[44] van Doorn JLM, Wijntjes J, Saris CGJ, et al. Association of diaphragm thickness and echogenicity with age, sex, and body mass index in healthy subjects. Muscle Nerve 2022; 66: 197–202. doi:10.1002/mus.27639 35583147 PMC9543748

[45] Da Conceição D, Perlas A, Giron Arango L, et al. Validation of a novel point-of-care ultrasound method to assess diaphragmatic excursion. Reg Anesth Pain Med 2024; 49: 800–804. doi:10.1136/rapm-2023-104983 37940349

[46] Chino K, Ohya T, Katayama K, et al. Diaphragmatic shear modulus at various submaximal inspiratory mouth pressure levels. Respir Physiol Neurobiol 2018; 252–253: 52–57. doi:10.1016/j.resp.2018.03.009 29567109

[47] Bachasson D, Dres M, Nierat MC, et al. Diaphragm shear modulus reflects transdiaphragmatic pressure during isovolumetric inspiratory efforts and ventilation against inspiratory loading. J Appl Physiol (1985) 2019; 126: 699–707. doi:10.1152/japplphysiol.01060.2018 30730816

[48] Ciloglu O, Karaali E, Gorgulu FF, et al. Diaphragm thickness and stiffness in patients with hyperkyphosis due to osteoporotic vertebral fracture: an ultrasonographic and elastographic study. Pol J Radiol 2020; 85: e575–e580. doi:10.5114/pjr.2020.99751 33204371 PMC7654313

[49] Flatres A, Aarab Y, Nougaret S, et al. Real-time shear wave ultrasound elastography: a new tool for the evaluation of diaphragm and limb muscle stiffness in critically ill patients. Crit Care 2020; 24: 34. doi:10.1186/s13054-020-2745-6 32014005 PMC6998330

[50] Fossé Q, Poulard T, Nierat MC, et al. Ultrasound shear wave elastography for assessing diaphragm function in mechanically ventilated patients: a breath-by-breath analysis. Crit Care 2020; 24: 669. doi:10.1186/s13054-020-03338-y 33246478 PMC7695240

[51] Aarab Y, Flatres A, Garnier F, et al. Shear wave elastography, a new tool for diaphragmatic qualitative assessment: a translational study. Am J Respir Crit Care Med 2021; 204: 797–806. doi:10.1164/rccm.202011-4086OC 34255974

[52] Xu JH, Wu ZZ, Tao FY, et al. Ultrasound shear wave elastography for evaluation of diaphragm stiffness in patients with stable COPD: a pilot trial. J Ultrasound Med 2021; 40: 2655–2663. doi:10.1002/jum.15655 33615538

[53] Chen Y, Li J, Dong B, et al. Two-dimensional shear wave elastography: a new tool for evaluating respiratory muscle stiffness in chronic obstructive pulmonary disease patients. BMC Pulm Med 2022; 22: 441. doi:10.1186/s12890-022-02231-4 36424581 PMC9686016

[54] Şendur HN, Cerit MN, Şendur AB, et al. Evaluation of diaphragm thickness and stiffness using ultrasound and shear-wave elastography. Ultrasound Q 2022; 38: 89–93. doi:10.1097/RUQ.0000000000000593 35001026

[55] Zhang J, Zhang C, Yan L, et al. Shear wave elastography of the diaphragm in acute exacerbation of chronic obstructive pulmonary disease: a prospective observational study. Medicine (Baltimore) 2023; 102: e33329. doi:10.1097/MD.0000000000033329 36930088 PMC10019183

[56] Zhang T, Liu Y, Xu D, et al. Diaphragm assessment by multimodal ultrasound imaging in healthy subjects. Int J Gen Med 2024; 17: 4015–4024. doi:10.2147/IJGM.S478136 39290234 PMC11406537

[57] Ye X, Xiao H, Bai W, et al. Two-dimensional strain ultrasound speckle tracking as a novel approach for the evaluation of right hemidiaphragmatic longitudinal deformation. Exp Ther Med 2013; 6: 368–372. doi:10.3892/etm.2013.1133 24137190 PMC3786841

[58] Hatam N, Goetzenich A, Rossaint R, et al. A novel application for assessing diaphragmatic function by ultrasonic deformation analysis in noninvasively ventilated healthy young adults. Ultraschall Med 2014; 35: 540–546. doi:10.1055/s-0034-1366090 24647765

[59] Orde SR, Boon AJ, Firth DG, et al. Diaphragm assessment by two-dimensional speckle tracking imaging in normal subjects. BMC Anesthesiol 2016; 16: 43. doi:10.1186/s12871-016-0201-6 27456490 PMC4960718

[60] Goutman SA, Hamilton JD, Swihart B, et al. Speckle tracking as a method to measure hemidiaphragm excursion. Muscle Nerve 2017; 55: 125–127. doi:10.1002/mus.25380 27533320

[61] Oppersma E, Hatam N, Doorduin J, et al. Functional assessment of the diaphragm by speckle tracking ultrasound during inspiratory loading. J Appl Physiol (1985) 2017; 123: 1063–1070. doi:10.1152/japplphysiol.00095.2017 28522757

[62] Fritsch SJ, Hatam N, Goetzenich A, et al. Speckle tracking ultrasonography as a new tool to assess diaphragmatic function: a feasibility study. Ultrasonography 2022; 41: 403–415. doi:10.14366/usg.21044 34749444 PMC8942740

[63] Xu Q, Yang X, Qian Y, et al. Comparison of assessment of diaphragm function using speckle tracking between patients with successful and failed weaning: a multicentre, observational, pilot study. BMC Pulm Med 2022; 22: 459. doi:10.1186/s12890-022-02260-z 36456940 PMC9716762

[64] Li R, Zhou Y, Chen W, et al. Speckle tracking ultrasound as a new tool to predict the weaning outcome of mechanical ventilation patients: a prospective observational study. Front Med (Lausanne) 2024; 11: 1449938. doi:10.3389/fmed.2024.1449938 39712177 PMC11658974

[65] Watanabe S, Sekiguchi K, Suehiro H, et al. Decreased diaphragm moving distance measured by ultrasound speckle tracking reflects poor prognosis in amyotrophic lateral sclerosis. Clin Neurophysiol Pract 2024; 9: 252–260. doi:10.1016/j.cnp.2024.10.002 39534515 PMC11554585

[66] Fayssoil A, Nguyen LS, Ogna A, et al. Diaphragm sniff ultrasound: normal values, relationship with sniff nasal pressure and accuracy for predicting respiratory involvement in patients with neuromuscular disorders. PLoS One 2019; 14: e0214288. doi:10.1371/journal.pone.0214288 31017911 PMC6481788

[67] Soilemezi E, Savvidou S, Sotiriou P, et al. Tissue Doppler imaging of the diaphragm in healthy subjects and critically ill patients. Am J Respir Crit Care Med 2020; 202: 1005–1012. doi:10.1164/rccm.201912-2341OC 32614246 PMC7528801

[68] Cammarota G, Boniolo E, Santangelo E, et al. Diaphragmatic kinetics assessment by tissue Doppler imaging and extubation outcome. Respir Care 2021; 66: 983–993. doi:10.4187/respcare.08702 33906957

[69] Benli RK, Yurdalan U, Yilmaz B, et al. Effect of post-extubation inspiratory muscle training on diaphragmatic function in mechanically ventilated patients: a randomized controlled trial. Adv Clin Exp Med 2024; 33: 1077–1085. doi:10.17219/acem/174815 38230846

[70] Xin S, Li Y, Liu R, et al. Tissue Doppler imaging of the diaphragm and outcome of weaning from mechanical ventilation. Australas J Ultrasound Med 2024; 27: 159–166. doi:10.1002/ajum.12389 39328254 PMC11423432

[71] Skaarup SH, Lonni S, Quadri F, et al. Ultrasound evaluation of hemidiaphragm function following thoracentesis: a study on mechanisms of dyspnea related to pleural effusion. J Bronchology Interv Pulmonol 2020; 27: 172–178. doi:10.1097/LBR.0000000000000627 31651544

[72] Fjaellegaard K, Koefod Petersen J, Alstrup G, et al. Ultrasound in predicting improvement in dyspnoea after therapeutic thoracentesis in patients with recurrent unilateral pleural effusion. Eur Clin Respir J 2024; 11: 2337446. doi:10.1080/20018525.2024.2337446 38711600 PMC11073413

[73] Nørskov J, Skaarup SH, Bendixen M, et al. Diaphragmatic dysfunction is associated with postoperative pulmonary complications and phrenic nerve paresis in patients undergoing thoracic surgery. J Anesth 2024; 38: 386–397. doi:10.1007/s00540-024-03325-5 38546897 PMC11096220

[74] Ando R, Ohya T, Kusanagi K, et al. Effect of inspiratory resistive training on diaphragm shear modulus and accessory inspiratory muscle activation. Appl Physiol Nutr Metab 2020; 45: 851–856. doi:10.1139/apnm-2019-0906 32049562

[75] Li C, Liu Y, Dong R, et al. Deep learning radiomics on shear wave elastography and B-mode ultrasound videos of diaphragm for weaning outcome prediction. Med Eng Phys 2024; 123: 104090. doi:10.1016/j.medengphy.2023.104090 38365343

[76] Brignol A, Cheriet F, Aubin-Fournier JF, et al. Robust unsupervised texture segmentation for motion analysis in ultrasound images. Int J Comput Assist Radiol Surg 2025; 20: 97–106. doi:10.1007/s11548-024-03249-1 39289317

[77] Zhang Q, Yang D, Zhu Y, et al. An optimized optical-flow-based method for quantitative tracking of ultrasound-guided right diaphragm deformation. BMC Med Imaging 2023; 23: 108. doi:10.1186/s12880-023-01066-7 37592200 PMC10436632

[78] Huang D, Song F, Luo B, et al. Using automatic speckle tracking imaging to measure diaphragm excursion and predict the outcome of mechanical ventilation weaning. Crit Care 2023; 27: 18. doi:10.1186/s13054-022-04288-3 36639710 PMC9840291

[79] Ye X, Liu Z, Ma Y, et al. A novel normalized cross-correlation speckle-tracking ultrasound algorithm for the evaluation of diaphragm deformation. Front Med (Lausanne) 2021; 8: 612933. doi:10.3389/fmed.2021.612933 33777969 PMC7994279

[80] Loizou CP, Chrysostomou C, Minas G, et al. Ultrasound diaphragmatic manual and semi-automated motion measurements: application in simulated and *in vivo* data of critically ill subjects. Comput Methods Programs Biomed 2020; 194: 105517. doi:10.1016/j.cmpb.2020.105517 32446038

[81] Loizou CP, Matamis D, Minas G, et al. A new method for diaphragmatic maximum relaxation rate ultrasonographic measurement in the assessment of patients with diaphragmatic dysfunction. IEEE J Transl Eng Health Med 2018; 6: 2700710. doi:10.1109/JTEHM.2018.2868671 30405977 PMC6204329

[82] Wachinger C, Yigitsoy M, Rijkhorst EJ, et al. Manifold learning for image-based breathing gating in ultrasound and MRI. Med Image Anal 2012; 16: 806–818. doi:10.1016/j.media.2011.11.008 22226466

[83] Xu Q, Hamilton RJ. A novel respiratory detection method based on automated analysis of ultrasound diaphragm video. Med Phys 2006; 33: 916–921. doi:10.1118/1.2178451 16696466

[84] Dietrich CF, Averkiou M, Nielsen MB, et al. How to perform contrast-enhanced ultrasound (CEUS). Ultrasound Int Open 2018; 4: E2–E15. doi:10.1055/s-0043-123931 29423461 PMC5802984

[85] Fischer C, Krix M, Weber MA, et al. Contrast-enhanced ultrasound for musculoskeletal applications: a World Federation for Ultrasound in Medicine and Biology position paper. Ultrasound Med Biol 2020; 46: 1279–1295. doi:10.1016/j.ultrasmedbio.2020.01.028 32139152

[86] Maurits NM, Bollen AE, Windhausen A, et al. Muscle ultrasound analysis: normal values and differentiation between myopathies and neuropathies. Ultrasound Med Biol 2003; 29: 215–225. doi:10.1016/S0301-5629(02)00758-5 12659909

[87] Heckmatt JZ, Pier N, Dubowitz V. Real-time ultrasound imaging of muscles. Muscle Nerve 1988; 11: 56–65. doi:10.1002/mus.880110110 3277050

[88] Pillen S, Tak RO, Zwarts MJ, et al. Skeletal muscle ultrasound: correlation between fibrous tissue and echo intensity. Ultrasound Med Biol 2009; 35: 443–446. doi:10.1016/j.ultrasmedbio.2008.09.016 19081667

[89] Prado-Costa R, Rebelo J, Monteiro-Barroso J, et al. Ultrasound elastography: compression elastography and shear-wave elastography in the assessment of tendon injury. Insights Imaging 2018; 9: 791–814. doi:10.1007/s13244-018-0642-1 30120723 PMC6206379

[90] Gennisson JL, Deffieux T, Fink M, et al. Ultrasound elastography: principles and techniques. Diagn Interv Imaging 2013; 94: 487–495. doi:10.1016/j.diii.2013.01.022 23619292

[91] Creze M, Nordez A, Soubeyrand M, et al. Shear wave sonoelastography of skeletal muscle: basic principles, biomechanical concepts, clinical applications, and future perspectives. Skeletal Radiol 2018; 47: 457–471. doi:10.1007/s00256-017-2843-y 29224123

[92] Leitman M, Lysyansky P, Sidenko S, et al. Two-dimensional strain-a novel software for real-time quantitative echocardiographic assessment of myocardial function. J Am Soc Echocardiogr 2004; 17: 1021–1029. doi:10.1016/j.echo.2004.06.019 15452466

[93] Singh A, Voss WB, Lentz RW, et al. The diagnostic and prognostic value of echocardiographic strain. JAMA Cardiol 2019; 4: 580–588. doi:10.1001/jamacardio.2019.1152 31042262

[94] Collier P, Phelan D, Klein A. A test in context: myocardial strain measured by speckle-tracking echocardiography. J Am Coll Cardiol 2017; 69: 1043–1056. doi:10.1016/j.jacc.2016.12.012 28231932

[95] Isaaz K, Thompson A, Ethevenot G, et al. Doppler echocardiographic measurement of low velocity motion of the left ventricular posterior wall. Am J Cardiol 1989; 64: 66–75. doi:10.1016/0002-9149(89)90655-3 2741815

[96] Kadappu KK, Thomas L. Tissue Doppler imaging in echocardiography: value and limitations. Heart Lung Circ 2015; 24: 224–233. doi:10.1016/j.hlc.2014.10.003 25465516

[97] Cameli M, Mondillo S, Solari M, et al. Echocardiographic assessment of left ventricular systolic function: from ejection fraction to torsion. Heart Fail Rev 2016; 21: 77–94. doi:10.1007/s10741-015-9521-8 26712329

[98] Boussuges A, Gole Y, Blanc P. Diaphragmatic motion studied by m-mode ultrasonography: methods, reproducibility, and normal values. Chest 2009; 135: 391–400. doi:10.1378/chest.08-1541 19017880

[99] Jonkman AH, Wennen M, Sklar MC, et al. Tissue doppler imaging of the diaphragm: a novel approach but too early for clinical implementation? Am J Respir Crit Care Med 2020; 202: 1741–1742. doi:10.1164/rccm.202007-2958LE 32961066 PMC7737574

[100] Spiesshoefer J, Herkenrath S, Henke C, et al. Evaluation of respiratory muscle strength and diaphragm ultrasound: normative values, theoretical considerations, and practical recommendations. Respiration 2020; 99: 369–381. doi:10.1159/000506016 32396905

[101] Jansen M, van Alfen N, Nijhuis van der Sanden MW, et al. Quantitative muscle ultrasound is a promising longitudinal follow-up tool in Duchenne muscular dystrophy. Neuromuscul Disord 2012; 22: 306–317. doi:10.1016/j.nmd.2011.10.020 22133654

[102] Pillen S, Verrips A, van Alfen N, et al. Quantitative skeletal muscle ultrasound: diagnostic value in childhood neuromuscular disease. Neuromuscul Disord 2007; 17: 509–516. doi:10.1016/j.nmd.2007.03.008 17537635

[103] Puthucheary ZA, Phadke R, Rawal J, et al. Qualitative ultrasound in acute critical illness muscle wasting. Crit Care Med 2015; 43: 1603–1611. doi:10.1097/CCM.0000000000001016 25882765

[104] Dominelli PB, Archiza B, Ramsook AH, et al. Effects of respiratory muscle work on respiratory and locomotor blood flow during exercise. Exp Physiol 2017; 102: 1535–1547. doi:10.1113/EP086566 28841267

[105] Neviere R, Mathieu D, Chagnon JL, et al. Skeletal muscle microvascular blood flow and oxygen transport in patients with severe sepsis. Am J Respir Crit Care Med 1996; 153: 191–195. doi:10.1164/ajrccm.153.1.8542115 8542115

[106] Ait-Oufella H, Joffre J, Boelle PY, et al. Knee area tissue oxygen saturation is predictive of 14-day mortality in septic shock. Intensive Care Med 2012; 38: 976–983. doi:10.1007/s00134-012-2555-7 22527071

[107] Kidane B, Chadi SA, Di Labio A, et al. Soft tissue oxygenation and risk of mortality (STORM): an early marker of critical illness? J Crit Care 2015; 30: 315–320. doi:10.1016/j.jcrc.2014.11.004 25434719

[108] Vorwerk C, Coats TJ. The prognostic value of tissue oxygen saturation in emergency department patients with severe sepsis or septic shock. Emerg Med J 2012; 29: 699–703. doi:10.1136/emermed-2011-200160 21946179

[109] Aubier M, Trippenbach T, Roussos C. Respiratory muscle fatigue during cardiogenic shock. J Appl Physiol Respir Environ Exerc Physiol 1981; 51: 499–508. 6790504 10.1152/jappl.1981.51.2.499

[110] Aubier M, Viires N, Syllie G, et al. Respiratory muscle contribution to lactic acidosis in low cardiac output. Am Rev Respir Dis 1982; 126: 648–652. 6214978 10.1164/arrd.1982.126.4.648

[111] Horn AG, Baumfalk DR, Schulze KM, et al. Effects of elevated positive end-expiratory pressure on diaphragmatic blood flow and vascular resistance during mechanical ventilation. J Appl Physiol (1985) 2020; 129: 626–635. doi:10.1152/japplphysiol.00320.2020 32730173 PMC7517429

[112] Davis RT, 3rd, Bruells CS, Stabley JN, et al. Mechanical ventilation reduces rat diaphragm blood flow and impairs oxygen delivery and uptake. Crit Care Med 2012; 40: 2858–2866. doi:10.1097/CCM.0b013e31825b933a 22846782 PMC3455118

