# Supplementary material for: Ultrasound innovations in diaphragm assessment: an integrative review of expanding clinical applications
Source: Eur Respir Rev. 2025 Oct 8;34(178):250089. doi: 10.1183/16000617.0089-2025 (PMC12505151; doi:10.1183/16000617.0089-2025)
Supplement: Supplementary file 1 [file ERR-0089-2025.SUPPLEMENT1.pdf]

Supplementary information 1
# - Search strategy

Groups of key words

1. **Diaphragm muscle/structure**
   - *"diaphragm*" [Title/Abstract]*
2. **General Ultrasound Terms**
   - *"ultrasound" [Title/Abstract]*
   - *"sonograph*" [Title/Abstract]*
   - *"echo imaging" [Title/Abstract]*
   - *"ultrason*" [Title/Abstract]*
   - *"acoustic imaging "[Title/Abstract]*
   - *“doppler imaging"[Title/Abstract]*
   - *"Point of Care Ultrasound" [Title/Abstract]*
   - *"POCUS" [Title/Abstract]*
   - *“elastograph*” [Title/Abstract]*
3. **Advanced Techniques**
   - Related to: **Novelty.**
     - *“advanc*” [Title/Abstract]*
     - *“concept*” [Title/Abstract]*
     - *“emerging” [Title/Abstract]*
     - *“innovat*” [Title/Abstract]*
     - *“original” [Title/Abstract]*
     - *“proof-of-concept*” [Title/Abstract]*
     - *“state-of-the-art” [Title/Abstract]*
     - *“new” [Title/Abstract]*
     - *“newly” [Title/Abstract]*
     - *“novel” [Title/Abstract]*
   - Related to: **Ultrasound technical features.**
     - *"3D" [Title/Abstract]*
     - *"color" [Title/Abstract]*
     - *“doppler” [Title/Abstract]*
     - *“echogenicity” [Title/Abstract]*
     - *“echo intensity” [Title/Abstract]*
     - *“echodensity” [Title/Abstract]*
     - *“echo texture” [Title/Abstract]*
     - *“kinetics” [Title/Abstract]*
     - *“kinematics” [Title/Abstract]*

    - *“shear-wave” [Title/Abstract]*
    - *“slope” [Title/Abstract]*
    - *“speed” [Title/Abstract]*
    - *“speckle-track*” [Title/Abstract]*
    - *“speckl*” [Title/Abstract]*
    - *“tissue doppler” [Title/Abstract]*
    - *“velocit*” [Title/Abstract]*
- Related to: **Blood flow.**
    - *“arter*” [Title/Abstract]*
    - *“blood flow” [Title/Abstract]*
    - *“blood velocity” [Title/Abstract]*
    - *“circulat*” [Title/Abstract]*
    - *“flow dynamic” [Title/Abstract]*
    - *“flow rate” [Title/Abstract]*
    - *“microcirculat*” [Title/Abstract]*
    - *"perfusion*" [Title/Abstract]*
    - *“vascular*” [Title/Abstract]*
    - *“venous” [Title/Abstract]*
- Related to: **Biomechanical proprieties.**
    - *“biomechamic*” [Title/Abstract]*
    - *“deformation” [Title/Abstract]*
    - *“elastic*” [Title/Abstract]*
    - *“fatigue” [Title/Abstract]*
    - *“mechanical propert*” [Title/Abstract]*
    - *“shear” [Title/Abstract]*
    - *“strain” [Title/Abstract]*
    - *“stiffness” [Title/Abstract]*
    - *“stress*” [Title/Abstract]*
    - *“viscoelastic*” [Title/Abstract]*

<u>Filters</u>

Language: English, French

<u>Search equation</u>

("diaphragm*"[Title/Abstract] AND ("ultrasound"[Title/Abstract] OR "sonograph*"[Title/Abstract] OR "echo imaging"[Title/Abstract] OR "ultrason*"[Title/Abstract] OR "sonograph* exam*"[Title/Abstract] OR "acoustic imaging"[Title/Abstract] OR "doppler imaging"[Title/Abstract] OR "point of care ultrasound"[Title/Abstract] OR "pocus"[Title/Abstract] OR "elastograph*"[Title/Abstract]) AND ("advanc*"[Title/Abstract] OR "concept*"[Title/Abstract] OR "emerging"[Title/Abstract] OR "innovat*"[Title/Abstract] OR "original*"[Title/Abstract] OR "proof of concept*"[Title/Abstract] OR "state of the art"[Title/Abstract] OR

"new"[Title/Abstract] OR "newly"[Title/Abstract] OR "novel"[Title/Abstract] OR "3D"[Title/Abstract] OR "color*"[Title/Abstract] OR "doppler*"[Title/Abstract] OR "echogenicity*"[Title/Abstract] OR "echo intensity*"[Title/Abstract] OR "echodensity*"[Title/Abstract] OR "echo texture*"[Title/Abstract] OR "shear wave"[Title/Abstract] OR "slope"[Title/Abstract] OR "speed"[Title/Abstract] OR "speckle track*"[Title/Abstract] OR "speckl*"[Title/Abstract] OR "tissue doppler"[Title/Abstract] OR "velocit*"[Title/Abstract] OR "kinetics"[Title/Abstract] OR "kinematics"[Title/Abstract] OR "arter*"[Title/Abstract] OR "blood flow"[Title/Abstract] OR "blood velocity"[Title/Abstract] OR "circulat*"[Title/Abstract] OR "flow dynamic*"[Title/Abstract] OR "flow rate"[Title/Abstract] OR "microcirculat*"[Title/Abstract] OR "perfusion*"[Title/Abstract] OR "vascular*"[Title/Abstract] OR "venous*"[Title/Abstract] OR "biomechanic**"[Title/Abstract] OR "deformation*"[Title/Abstract] OR "elastic*"[Title/Abstract] OR "fatigue*"[Title/Abstract] OR "mechanical propert*"[Title/Abstract] OR "shear"[Title/Abstract] OR "strain"[Title/Abstract] OR "stiffness"[Title/Abstract] OR "stress*"[Title/Abstract] OR "viscoelastic*"[Title/Abstract])) AND (english[Filter] OR french[Filter])

PUBMED

January 14th, 2025 – 1,411 results
