# Supplementary material for: Ultrasound innovations in diaphragm assessment: an integrative review of expanding clinical applications
Source: Eur Respir Rev. 2025 Oct 8;34(178):250089. doi: 10.1183/16000617.0089-2025 (PMC12505151; doi:10.1183/16000617.0089-2025)
Supplement: Supplementary file 2 [file ERR-0089-2025.SUPPLEMENT2.pdf]

Supplementary information 2
# - Characteristics of included studies

**Author**	**Aarab Y, 2021**
**Country**	France
**Study design**	Prospective observational study, single-centre study
**Population**	- Critically ill patients<br>- Number of participants: 102 completed the study evaluation. 94 patients were available. For analysis with at least three diaphragm evaluations. 92 patients available for shear modulus data analysis<br>- Age: 60 ± 17 years.<br>- Male/female: 63 (70%) males<br>- The primary reasons for ICU admission varied, with respiratory distress being the most common, accounting for 56% of admissions. Other causes included cardiac arrest in 10%, coma or encephalopathy in 11%, sepsis in 13%, and acute heart failure in just 3%. A small proportion, 7%, were admitted for other reasons. During their ICU stay, 60% of the patients received neuromuscular blocking agents (NMBA), with a median infusion duration of 3 days (IQR: 2–5.5). Corticosteroids were used in 55% of cases, with a median infusion duration of 8 days (IQR: 4–12.3). Sedatives were administered to 86% of patients, with deep sedation lasting a median of 5 days (IQR: 3–9). Invasive mechanical ventilation was required for 86% of the population, with a median duration of 7 days (IQR: 4–15), while continuous mandatory ventilation had a median duration of 2.8 days (IQR: 1.1–5.7). Notably, 14% of the patients did not require any form of ventilation. Vasopressors were used in 85% of the cases, with a median duration of 4 days (IQR: 3–7), and dialysis was needed for 26% of patients, lasting a median of 6 days (IQR: 3–8.5).<br>Regarding fluid management, the cumulative fluid balance at Day 3 was 4.7 ± 4.4 liters, increasing to 5.5 ± 5.4 liters by Day 7.
**New/advanced ultrasound approach and technical features**	**Ultrasound shear wave elastography (SWE) to assess diaphragm stiffness.**<br>• Timing of the assessment: ICU admission and repeatedly every other day up to Day 28, discharge from the ICU, or death.<br>• Position of participant: Supine, head of bed elevated to 30 degrees.<br>• Type of breathing effort: Assessments were performed during quiet breathing. All measurements were taken at the end of expiration when the diaphragm was at rest. Conscious subjects were instructed to stay relaxed, breathe as calmly as possible throughout the procedure, and hold their breath at functional residual capacity (FRC) for SWE acquisition for as long as they

	could. In the case of mechanically ventilated patients, an end-expiratory pause was implemented during SWE acquisition.<br>• Hemidiaphragm assessed: Right.<br>• Anatomical placement of the transducer/array: The transducer was placed via an intercostal approach at the zone of apposition, in the sagittal plane perpendicular to the diaphragm muscle and between the right anterior and midaxillary lines.<br>• Image acquisition procedure: Images were acquired using An Aixplorer ultrasonic scanner (SuperSonic Imagine) with a 4- to 15-MHz linear transducer (SL15-4; SuperSonic Imagine) in both B and SWE modes with musculoskeletal preset. At least 5mm of coupling gel was applied to minimize tissue distortion. The diaphragm was identified as a three-layered structure with two hyperechoic lines (pleural and peritoneal membranes) and a middle hypoechoic layer (diaphragm muscle fibers). Adjustment of the probe to obtain a longitudinal view with the acoustic beam parallel to the diaphragm muscle fascicles. Upon activating the SWE mode, a color-coded box indicating the region of interest was overlaid on the image. If a stable image with good quality criteria (homogeneous SWE color map) could not be obtained due to factors such as a high respiratory rate, the measured SM was excluded from data analysis. To ensure consistent image location in subsequent ultrasound assessments, a mark was carefully placed on the subject's chest during the initial measurement, and a standardised protocol was followed.<br>• Image analysis procedure: The largest possible area was outlined within the most homogeneous zone of the region of interest using the ultrasound system's built-in software (Q-Box trace) to minimize measurement bias, and shear modulus (SM) was recorded. Image analysis was also performed offline using Osirix DICOM Viewer software (Pixmeo, Geneva,Switzerland). The analysis involved reviewing acquired images and elastograms to ensure accurate measurements of shear modulus.<br>• Number of images analysed to retrieve results: Multiple images were acquired, with the exact number not specified, but the analysis was conducted in triplicate for each measurement.<br>• New Ultrasound markers: Shear modulus (SM) measured by shear wave elastography (SWE) in kilopascals (kPa) as an innovative ultrasound marker of diaphragm stiffness and mechanical properties. The calculation for SM was based on the formula $SM=r \cdot v^2$, where r is the tissue density (1,000 kg/m^3) and v is the shear wave velocity.
**Comparator(s)**	Diaphragmatic thickness, Transdiaphragmatic pressure.<br>Clinically, participants were divided into three groups based on the change in shear modulus (SM) from the baseline measurement to the last measurement

	obtained during the first week: >10% increase, <10% change, and >10% decrease.
**Reliability/Feasibility**	N/a
**Physiological and/or technical outcomes**	N/a
**Clinical outcomes**	Regarding diaphragm shear modulus (SM), it remained unchanged in 8% of patients, decreased by more than 10% in 41%, and increased by more than 10% in 51%. Adjusted multivariable analysis indicated that diaphragm SM decreased over time in older patients (β -0.05 ± 0.02, $p < 0.05$), those treated for sepsis (β -1.79 ± 0.82, $p = 0.03$), and those receiving steroids (β -0.11 ± 0.04, $p = 0.01$). Additionally, patients who showed increased diaphragmatic thickness during their ICU stay also experienced a decrease in SM (β -8.92 ± 4.46, $p = 0.03$). Time spent under controlled mechanical ventilation was associated with a decrease in SM compared to time spent under pressure support ventilation or without ventilatory assistance (β 1.45 ± 0.40, $p < 0.05$).<br>There was no significant association between changes in diaphragm shear modulus (SM) and ventilator-free days, length of stay in the ICU, difficulty in weaning, or mortality at Day 28.

**Author**	**Ando R, 2020**
**Country**	Japan
**Study design**	Randomised Controlled Trial, single-centre study
**Population**	- Type of population: Healthy subjects (Elite collegiate swimmers)<br>- Number of participants: 19, divided into a control group (n=9) and an inspiratory resistive training program group (n=10).<br>- Age: 19.3 ± 0.1 years<br>- Male/female: All male<br>- Control group: age: 19.3±0.1 years, height: 174.4±0.5 cm, body mass: 69.0±0.9 kg.<br>- Training group: age: 19.3±0.1 years, height: 173.5±0.6 cm, body mass: 68.9±0.8 kg.<br>- Detailed information about patients' disease: Not applicable (participants are healthy elite athletes)
**New/advanced ultrasound approach and technical features**	**Ultrasound shear wave elastography (SWE) to assess diaphragm stiffness.**<br><br>• Timing of the assessment: The assessment was performed at baseline and again after a 6-week inspiratory resistive training program.<br>• Position of participant: Sitting on a chair with backrest reclined at 75°.

	• Type of breathing effort: The breathing effort during assessments included submaximal efforts at various intensities (15%, 45%, and 75% of PImax).<br>• Hemidiaphragm assessed: Right.<br>• Anatomical placement of the transducer/array: The transducer was placed on the eighth intercostal space on the right side, between the anteroaxillary and midaxillary lines. The transducer was aligned along the direction of the diaphragm in the zone of apposition to obtain clear echoes from the pleural and peritoneal membranes.<br>• Image acquisition procedure: Ultrasound images were acquired using the Aixplorer system (SuperSonic Imagine, Aix-en-Provence, France) with a 4–15 MHz linear array probe (SL15-4). A region of interest (ROI, 1.5 cm × 4 cm) for shear wave image acquisition was placed. Images were acquired continuously at 2 Hz during resting breathing, selecting those at end expiration.<br>• Image analysis procedure: The average shear wave velocity was measured using built-in software (Q-Box trace) and converted into shear modulus.<br>• Number of images analysed to retrieve results: 5 images were acquired, and the average of the 3 with the lowest CV was used.<br>• New Ultrasound markers retrieved: The primary ultrasound marker introduced was the diaphragm's shear modulus (μ) – mean value, measured in kilopascals (kPa) and derived from the shear wave velocity (V). The average shear modulus at end-expiration and end-inspiration (for each target inspiratory level) was used to estimate the shear modulus during the PImax manoeuvre using a linear regression equation made from shear modulus values at submaximal inspirations.
**Comparator(s)**	SWE pre- and post-inspiratory resistive training program.
**Reliability/Feasibility**	N/a
**Physiological and/or technical outcomes**	N/a
**Clinical outcomes**	After the 6-week training program, inspiratory mouth pressures at 15%, 45%, and 75% of PImax were 23.3 ± 3.3 cmH2O, 69.8 ± 10.0 cmH2O, and 116.3 ± 16.7 cmH2O in the control group, and 24.4 ± 3.4 cmH2O, 73.2 ± 10.1 cmH2O, and 122.0 ± 16.8 cmH2O in the training group.<br>Diaphragm's shear modulus At inspiratory mouth pressures of 15%, 45%, and 75% of PImax, the control group had pre-training values of 58.7 ± 16.8, 96.9 ± 33.2, and 146.4 ± 55.7 kPa, increasing post-training to 70.6 ± 21.8, 138.2 ± 39.1, and 194.8 ± 47.1 kPa, respectively. The training group had pre-training values of 59.2 ± 17.9, 108.9 ± 24.0, and 149.9 ± 31.1 kPa, which increased post-training to 71.0 ± 24.3, 137.9 ± 39.3, and 186.7 ± 49.4 kPa, respectively.

	The estimated shear modulus of the diaphragm during the PImax manoeuvre (100%) significantly increased from baseline to 6 weeks in both the control (188.1 ± 21.9 kPa to 257.0 ± 22.1 kPa) and training groups (197.5 ± 12.1 kPa to 248.7 ± 20.1 kPa) ($p < 0.05$).

**Author**	Bachasson D, 2019
**Country**	France
**Study design**	Observational study (experimental) with randomised stepwise inspiratory loading protocol, single-centre study
**Population**	- Healthy adults<br>- Number of participants: 15<br>- Age: Male: mean 32 years (range 18-43), Female: mean 28 years (range 20-44)<br>- Male/Female: 11/4<br>- No specific disease characteristics mentioned as participants are healthy
**New/advanced ultrasound approach and technical features**	**Ultrasound shear wave elastography (SWE) to assess diaphragm stiffness.**<br><br>• Timing of the assessment: Not applicable, this was an experimental study on healthy participants.<br>• Position of participant: Participants were studied in a semirecumbent position (40°) with an uncast abdomen.<br>• Type of breathing effort: Controlled breathing maneuvers through a mouthpiece, including isovolumetric inspiratory efforts and ventilation against inspiratory threshold loading.<br>○ The isovolumetric inspiratory efforts were performed against closed airways at 10, 20, 30, 40, 50, and 60% PImax. Both apnea and inspiratory efforts were performed at FRC. Participants were asked to reach progressively the target Pmo and to maintain their effort for ~10. Each task was repeated twice. Tasks were alternated with 1–2 min of unloaded breathing.<br>○ The inspiratory threshold loading was applied stepwise at increments of 10% up to 50% of PImax. Each loading task was repeated twice, alternating 1-2 minutes of periods of unloaded breathing to allow for recovery.<br>Visual feedback was provided to participants to guide their efforts, and data were collected for analysis of diaphragm function under these loading conditions.<br>• Hemidiaphragm assessed: Right hemidiaphragm.

	• Anatomical placement of the transducer/array: The right hemidiaphragm was scanned at the zone of apposition, between the 8th and 10th intercostal spaces, with the transducer position carefully marked on the skin.<br>• Image acquisition procedure: Ultrasound measurements were performed using an Aixplorer Ultrasound scanner (V11.2; SuperSonic Imagine) with a 10- to 2-MHz linear transducer array (SL10-2). Settings were defined as follows: brightness mode (B-mode) enabled; supersonic shear wave imaging mode enabled (SWE); penetration mode enabled; tissue tuner at 1,540 m/s; and dynamic range at 80 dB. Gain and time gain compensation were tailored for each patient. Sampling rates for B-mode imaging and SWE were 12 and 2 Hz, respectively. Ultrasound measurements were performed by a trained operator. Ultrasound acquisition was synchronised with the PowerLab system to record ultrasound, flow, and pressure data together.<br>• Image analysis procedure: Offline image analysis was done in MATLAB (MathWorks, Natick, MA, USA), involving exporting the B-mode and SWE recordings, drawing an ROI within the shear modulus map, and calculating the diaphragm shear modulus (SMdi) based on the shear wave speed and muscle density.<br>• Number of images analysed to retrieve results: The document does not specify the exact number of images analysed. However, it suggests that several images were used, as the isovolumetric inspiratory efforts were performed against closed airways for a duration of 10 seconds, and the shear modulus results were averaged. Additionally, the authors indicate that each step of the protocol was performed twice, implying that at least two images were utilised for each assessment.<br>• New Ultrasound markers: Ultrasound shear wave elastography (SWE) measuring diaphragm shear modulus (SMdi) in kilopascals (kPa). The mean SMdi at functional residual capacity (FRC) during apnea was subtracted from the mean SMdi and the maximal SMdi (measured during inspiratory time) during isovolumetric efforts and ventilation, respectively.
**Comparator(s)**	Transdiaphragmatic pressure (Pdi)
**Reliability/Feasibility**	During isovolumetric inspiratory effort against closed airways, among the selected data, the mean of the coefficient of variation for SMdi was 16.2%.
**Physiological and/or technical outcomes**	- The shear modulus of the diaphragm (SMdi) during apnea at functional residual capacity (FRC) was recorded at 9.13 kPa (SD 2.17 kPa).<br>- <u>Isovolumetric Inspiratory Effort Against Closed Airways</u>: Repeated-measures ANOVA indicated a significant effect of inspiratory effort levels on both SMdi and transdiaphragmatic pressure (Pdi). The mean Pdi showed a significant correlation with the mean SMdi across all participants, with correlation coefficients ranging from 0.77 to 0.96 (all $p < 0.01$; $R = 0.82$, 95% CIs [0.76, 0.86]).

	- Ventilation Against Inspiratory Threshold Loading: Repeated-measures ANOVA revealed a significant effect of inspiratory threshold loading levels on SMdi and Pdi. The maximal SMdi was correlated with the Pdi swing in all participants, with correlation coefficients ranging from 0.40 to 0.90 (all $p < 0.01$; R = 0.70, 95% CIs [0.66, 0.73], $p < 0.001$).<br>- Other Observations:<br>In some participants, SMdi did not consistently increase with Pdi during both tasks, which may be attributed to factors such as transducer misalignment or variations in diaphragm recruitment strategies.<br>The relationship between SMdi and Pdi was less pronounced during isovolumetric efforts compared to ventilation against threshold loading, potentially due to differences in lung volume and diaphragm length between the two conditions.
**Clinical outcomes**	N/a

**Author**	**Benli RK, 2024**
**Country**	Türkiye
**Study design**	Randomised controlled trial, single-centre study
**Population**	- Type of population: Critically ill patients who were mechanically ventilated for more than 2 days and healthy individuals.<br>- Number of participants: 30 (20 patients and 10 healthy controls). Participants were then divided into 3 groups: inspiratory muscle training (IMT) group, conventional physiotherapy (CP) group and healthy controls (HCs).<br>- Age: The mean age was 62.8 ± 16.4 years for the CP group, 64.1 ± 8.2 years for the IMT group, and 51.8 ± 11.4 years for the healthy controls.<br>- Male/female: both the CP and IMT groups had 8 males and 2 females, while the healthy control group had an even distribution of 5 males and 5 females.<br>- The mean APACHE II scores were comparable, with 26.8 ± 4.5 for the CP group and 27.0 ± 4.4 for the IMT group ($p = 0.922$). The length of ICU stays averaged 7.6 ± 2.8 days for the CP group and 7.3 ± 2.0 days for the IMT group, while the overall hospital stays were 11.7 ± 1.8 days and 12.5 ± 5.0 days, respectively. Intubation time was slightly longer in the CP group at 4.5 ± 1.6 days compared to 3.9 ± 1.2 days in the IMT group.
**New/advanced ultrasound approach and technical features**	**The innovative ultrasound technique used in the study is Tissue Doppler Imaging (TDI).**<br><br>• Timing of the assessment: The assessments were performed after extubation and 5 days later.

	• Position of participant: Participants were positioned lying on their bed at an angle of 20–30°.<br>• Type of breathing effort: Deep breathing.<br>• Hemidiaphragm assessed: Right side.<br>• Anatomical placement of the transducer/array: The probe was positioned in the subcostal region between the midclavicular and anterior axillary lines.<br>• Image acquisition procedure: Images were acquired using a Mindray ultrasound device (Mindray, Shenzhen, China) with a 2.7 MHz convex probe for Tissue Doppler Imaging (TDI). The probe was angled to ensure that the ultrasound waves reached the diaphragm as perpendicularly as possible. A sample volume of 5.0 mm and a velocity scale of 20.6 cm/s were selected. Participants were instructed to engage in deep breathing during the TDI measurements following 8–10 tidal breaths.<br>• Image analysis procedure: Two parameters were measured from each TDI waveform: peak contraction velocity (PCV), which is the maximum diaphragmatic velocity during contraction, measured in cm/s, and peak relaxation velocity (PRV), which is the maximum diaphragmatic velocity during relaxation, also measured in cm/s.<br>• Number of images analysed to retrieve results: The maximum value from the three deep breaths was recorded.<br>• New Ultrasound markers: Peak contraction velocity (PCV) and peak relaxation velocity (PRV), measured in cm/s.
**Comparator(s)**	No comparator. TDI was applied to the three groups of participants.
**Reliability/Feasibility**	N/a
**Physiological and/or technical outcomes**	N/a
**Clinical outcomes**	The increase in PCV measurements was significantly higher in the Inspiratory Muscle Training (IMT) and Healthy Controls (HC) groups compared to the Conventional Physiotherapy (CP) group ($p = 0.028$ and $p = 0.015$, respectively). There was a statistically significant difference in the change in PRV pre- and post-intervention between the IMT and HC groups ($p = 0.029$ and $p = 0.020$, respectively).<br>In the CP group, diaphragm excursion measurements did not significantly change pre- and post-intervention ($p = 0.285$). In contrast, diaphragm excursion significantly increased in the HC and IMT groups post-intervention compared to pre-intervention ($p = 0.005$ for both groups).

**Author**	**Bird JD, 2024**

**Country**	Canada
**Study design**	Observational study (experimental design), single-centre study
**Population**	- Type of population: Healthy individuals<br>- Number of participants: 16<br>- Age: Mean age 28 ± 5 years<br>- Male/female: 10 males, 6 females<br>- Detailed information about patients' disease: Not applicable (participants were healthy individuals)
**New/advanced ultrasound approach and technical features**	**The innovative ultrasound technique is contrast-enhanced ultrasound (CEUS) used to quantify diaphragm blood flow.**<br><br>• Timing of the assessment: Not applicable. However, the assessments were performed on two separate days (were scheduled to align with the time of day and took place at least 48 hours apart and no more than seven days apart to reduce any potential impact of lifestyle on respiratory function).<br>• Position of participant: Seated position.<br>• Type of breathing effort: The breathing efforts included unloaded breathing and pseudorandomised graded inspiratory pressure threshold loading (unloaded, 10%, 18%, and 25% of MIP). Each trial included a 2-minute baseline period, followed by the participant visually targeting a square-wave mouth pressure waveform to maintain a breathing frequency of 15 breaths per minute and a duty cycle of 0.5 for 5 minutes.<br>• Hemidiaphragm assessed: Right side.<br>• Anatomical placement of the transducer/array: The transducer was placed over the costal diaphragm region (*"…to delineate regions of costal diaphragm, external obliques, intercostal muscles, and liver*).<br>• Image acquisition procedure: Image acquisition procedure: GE 9L-D transducer and Vivid E9 ultrasound machine in amplitude modulation mode, mechanical index of 0.09 to 0.11, depth of 3 to 3.5 cm, fundamental frequency of 4 MHz, dynamic range of 57 dB, images captured at 3 frames per second during constant-rate IV infusion of lipid-stabilised microbubbles (Definity, Lantheus Medical Inc.; 2.5 mL/min). Participants were guided during the final three breaths of each trial to achieve an end-expiratory apnea near functional residual capacity, and CEUS images were obtained following a destruction-replenishment sequence with minimal movement artifacts. Ultrasound cine loops were recorded for 15 seconds following breathing cessation and bubble destruction.<br>• Image analysis procedure: CEUS images were converted to .mov files and analysed using publicly available software (narnar, narnar.co). Acoustic

	intensity over time data determined for regions of interest, modeled using nonlinear least squares regression (Levenberg-Marquardt algorithm) and the following equation:<br>$y = A(1 - e^{-\beta t})$:<br>"*where y is the video intensity at pulsing interval, A is the plateau video intensity reflecting microvascular blood volume (MBV), and b is the rate of microbubble replenishment reflecting microvascular blood flux rate (MFR). The product of MBV and MFR is proportionate to microvascular blood flow for a given region* (ie, $\dot{Q}_{DIA}$)."<br>• Number of images analysed to retrieve results: The specific number of images analysed was not mentioned in the provided text. However, the authors indicate that ultrasound cine loops were recorded for 15 seconds after breathing ceased and the bubbles were destroyed.<br>• New Ultrasound markers: Obtained from CEUS assessment - Microvascular blood volume of the diaphragm (MBV_{DIA}) in acoustic units (AU), microvascular blood flux rate of the diaphragm (MFR_{DIA}) measured per second (/s), diaphragm blood flow ($\dot{Q}_{DIA}$) measured in acoustic units per second (AU/s), and Vascular conductance of the diaphragm (VC_{DIA}) measured in AU/s/mmHg.
**Comparator(s)**	No comparator.
**Reliability/Feasibility**	The study found that the contrast-enhanced ultrasound (CEUS) technique used to measure relative diaphragm blood flow ($\dot{Q}_{DIA}$) had good to excellent test-retest reliability, with an intraclass correlation coefficient (ICC) of 0.86 (0.77, 0.92), and excellent interanalyzer reproducibility, with an ICC of 0.93 (0.90, 0.95).
**Physiological and/or technical outcomes**	Transdiaphragmatic pressure for unloaded and each loading stage (unloaded, 10%, 18%, and 25% of MIP) were 15.2 ± 0.8, 26.1 ± 0.8, 34.6 ± 0.8, and 40.0 ± 0.8 percentage of the maximum, respectively. $\dot{Q}_{DIA}$ increased with each stage of loading (3.1 ± 3.1, 6.9 ± 3.6, 11.0 ± 4.9, and 13.5 ± 5.4 acoustic units/s; $p < 0.0001$).<br>Muscle blood volume (MBV) of the diaphragm remained consistent across loads and days, while diaphragm muscle flow rate (MFR) and $\dot{Q}_{DIA}$ increased with load but were similar across days.<br>Increased transdiaphragmatic pressure generation correlated with higher $\dot{Q}_{DIA}$ and VC_{DIA} ($p < 0.0001$; partial eta squared > 0.94), also showing reproducibility across days ($p > 0.34$).
**Clinical outcomes**	N/a

**Author**	**Cammarota G, 2021**
**Country**	Italy

**Study design**	Prospective observational study, single-centre study
**Population**	- Critically ill patients, who had undergone invasive mechanical ventilation for more than 24 hours and were extubated after passing a spontaneous breathing trial (SBT) conducted in CPAP of 5 cm H_2O.<br>- Number of participants: 100<br>- Age: median 66.0 years (52.0-77.0) divided in the extubation success group, and median 66.0 years (62.5-73.0) in the extubation failure group.<br>- Male/Female: 42 (53.2%) males in the extubation success group, 12 (57.1%) in the extubation failure group.<br>- SAPS II at admission was median 30.0 (24.0–42.0) for extubation success group and median 39.0 (33.5–46.5) for extubation failure group; Ventilation duration before extubation was median 2.0 (1.0–5.0) days for extubation success group and median 3.0 (1.0–7.0) days for extubation failure group.
**New/advanced ultrasound approach and technical features**	**The innovative ultrasound technique used in this study is diaphragmatic Tissue Doppler imaging (TDI).**<br><br>• Timing of the assessment: The assessment was performed at the end of a spontaneous breathing trial (SBT).<br>• Position of participant: Participants were positioned in a semi-recumbent position (30°) during the assessment.<br>• Type of breathing effort: The breathing effort was assessed during quiet breathing while on continuous positive airway pressure (CPAP, 5 cmH2O).<br>• Hemidiaphragm assessed: Right side.<br>• Anatomical placement of the transducer/array: between midclavicular and anterior axillary line in the subcostal region.<br>• Image acquisition procedure: Portable ultrasound machine (Xario 200, Canon Medical Systems) with a sectorial probe (1.8-4.2 MHz) and cardiac tissue Doppler application. With the ultrasound beam oriented perpendicularly to the middle or posterior third of the diaphragm, the tissue Doppler application was activated, allowing for a clear diaphragm signal by adjusting the gain and filtering out high-frequency signals. After aligning the Doppler cursor with the direction of diaphragmatic displacement, a maximum amplitude sample box (20 mm) was placed along the diaphragmatic line to measure its excursion. The velocity scale was set to the lowest possible value to capture the slower velocity of diaphragmatic movement in relation to the beating heart.<br>• Image analysis procedure: While not explicitly detailed, it appears to be conducted using the built-in software of the ultrasound device immediately after image acquisition. The analysis was averaged over three consecutive

	breaths, stored electronically, and performed at the end of the SBT by a single operator.<br>• Number of images analysed to retrieve results: A total of 3 images per hemidiaphragm were analysed.<br>• New Ultrasound markers: Inspiratory peak velocity (cm/s), Inspiratory mean velocity (cm/s), Inspiratory acceleration (cm/s^2), Peak relaxation velocity (cm/s), Expiratory mean velocity (cm/s), Expiratory acceleration (cm/s^2).
**Comparator(s)**	The study did not directly compare TDI against another technique. Instead, TDI was incorporated into a modified version of the RSBI. Additionally, the population was analysed by categorizing patients into weaning success and weaning failure groups.
**Reliability/Feasibility**	**Intra-observer Reliability:**<br>The intra-observer reliability for various respiratory parameters was assessed using Pearson correlation coefficients and intraclass correlation coefficients (ICC). Assessor 1 showed high reliability for Inspiratory Peak Velocity (0.98, 95% CI: 0.97–0.99), Inspiratory Velocity-Time Integral (0.97, 95% CI: 0.95–0.99), and Expiratory Peak Velocity (0.96, 95% CI: 0.94–0.98), with ICC values ranging from 0.86 to 0.98. Assessor 2 also demonstrated strong reliability, particularly for Inspiratory Peak Velocity (0.97, 95% CI: 0.96–0.98) and Expiratory Peak Velocity (0.98, 95% CI: 0.98–0.99), with ICC values between 0.87 and 0.98.<br>**Inter-observer Reliability:**<br>Inspiratory Peak Velocity (0.97, 95% CI: 0.95–0.98) and Inspiratory Velocity-Time Integral (0.96, 95% CI: 0.94–0.97) showed the strongest correlations. ICC values ranged from 0.85 (95% CI: 0.76–0.91) to 0.97 (95% CI: 0.95–0.98), reflecting good agreement between the two assessors.
**Physiological and/or technical outcomes**	N/a
**Clinical outcomes**	In this study, diaphragmatic excursion kinetics were evaluated using dTDI for a total of 300 breaths, with 237 breaths from the extubation success group and 63 from the extubation failure group. At the end of the spontaneous breathing trial (SBT), the results showed no differences in inspiratory excursion (inspiratory velocity-time integral); however, extubation success subjects exhibited significantly lower inspiratory peak velocity (1.8 cm/s vs. 3.1 cm/s, $p < .001$), mean velocity (1.1 cm/s vs. 1.6 cm/s, $p < .001$), and acceleration (4.2 cm/s^2 vs. 8.8 cm/s^2, $p = .002$) compared to extubation failure subjects. During expiration, extubation failure subjects demonstrated higher peak relaxation velocity (1.8 cm/s vs. 2.6 cm/s, $p < .001$), mean velocity (0.9 cm/s vs. 1.1 cm/s, $P = .002$), and acceleration (7.1 cm/s^2 vs. 11.2 cm/s^2, $p = .004$).<br>The study also assessed the predictive accuracy of dTDI variables and conventional and diaphragmatic RSBIs for extubation failure. The area under the

	curve (AUC) for inspiratory peak velocity was 0.80 (P < .001), with a cutoff of >2.2 cm/s, sensitivity of 76.2%, and specificity of 62.0%. Inspiratory mean velocity also had an AUC of 0.80 (P < .001), with a cutoff of >1.4 cm/s, sensitivity of 71.4%, and specificity of 77.2%.<br>The AUC values for the inspiratory peak and mean velocities were wider than those obtained for the conventional (vs inspiratory peak velocity, p = 0.036; vs inspiratory mean velocity, p = 0.042) and diaphragmatic RSBIs (vs inspiratory peak velocity, p = 0.01; vs inspiratory mean velocity, p = 0.005).

**Author**	Chen Y, 2022
**Country**	China
**Study design**	Prospective observational study, single-centre study
**Population**	- Type of population: COPD patients and healthy adults.<br>- Number of participants: 239 (219 COPD patients, 20 healthy adults). COPD patients divided into mild (n = 82), moderate (n = 52), severe (n = 46), and very severe (n = 39) groups<br>- Age: Control group - mean 66.30 years (9.65); Mild COPD group - 68.94 years (8.21); Moderate COPD group - 64.44 years (9.69); Severe COPD group- 67.76 years (8.00); Very Severe COPD group - 65.38 years (9.52).<br>- Male/female: Control group - 18 males (90%), 2 females (10%); Mild COPD group - 72 males (87%), 10 females (13%); Moderate COPD group- 48 males (92%), 4 females (8%); Severe COPD group - 40 males (87%), 6 females (13%); Very Severe COPD group - 39 males (100%), 0 females (0%).<br>- In Mild COPD, the GOLD classification shows 20% in level A, 26% in level B, 10% in level C, and 44% in level D, with an mMRC score of 1 [IQR 0–1] and a CAT score of 13 [IQR 9-18]. The FEV1% predicted value is 87.72 (5.20), and the FEV1/FVC ratio is 59.17 (5.52).<br>- In Moderate COPD, there are no patients in GOLD level A, while 50% are in level B and 50% in level D. The mMRC score is 1.5 [IQR 1–2], and the CAT score is 22 [IQR 18–27.75]. The FEV1% predicted value is 64.56 (8.51), with an FEV1/FVC ratio of 55.29 (5.04).<br>- Severe COPD has no patients in levels A or C, with 35% in level B and 65% in level D. The mMRC score is 2 [IQR 2–3], and the CAT score is 32 [IQR 29-34]. The FEV1% predicted value is 39.89 (5.24), and the FEV1/FVC ratio is 46.65 (6.74).<br>- In Very Severe COPD, there are no patients in levels A or C, with 26% in level B and 74% in level D. The mMRC score is 3 [IQR 2–4], and the CAT score is 34

	[IQR 32–37]. The FEV1% predicted value is 20.85 (5.66), and the FEV1/FVC ratio is 36.15 (7.03).
**New/advanced ultrasound approach and technical features**	**Ultrasound shear wave elastography (SWE) to assess diaphragm stiffness.**<br><br>• Timing of the assessment: Not applicable.<br>• Position of participant: Participants were instructed to remain in a left lying position during the assessment.<br>• Type of breathing effort: Calmly holding breath at the end of inhalation.<br>• Hemidiaphragm assessed: Right side.<br>• Anatomical placement of the transducer/array: The transducer was placed under the rib between the anterior to mid-axillary line, approximately at the 6th to 12th intercostal space.<br>• Image acquisition procedure: High-resolution ultrasonic diagnostic apparatus (Aixplorer, Supersonic Imagine, Provence, France) with a 4-15 MHz frequency linear array probe. The image is stabilised, SWE mode is switched on, and elastic range adjusted to 0-160 kPa. Measurements are taken while the subject holds their breath at end-inhalation.<br>• Image analysis procedure: Circular ROI selected (1 mm for diaphragm). Analysis performed immediately during acquisition.<br>• New Ultrasound markers: Elastic modulus of the diaphragm during inspiratory hold, measured in kilopascals (kPa).
**Comparator(s)**	Lung function
**Reliability/Feasibility**	The reliability of diaphragm elastic modulus measurements performed by the same physician using 2D-SWE technology showed good repeatability. In this assessment, 5% (1/20) of the differences between the two diaphragm elastic modulus measurements were outside of the consistency limit. The mean difference between the two measurements was -0.42 ± 1.65, with a 95% agreement limit ranging from -3.65 to 2.81. The intraclass correlation coefficient (ICC) was 0.756, with a 95% confidence interval (CI) of 0.482 to 0.896.<br>When different physicians measured the diaphragm elastic modulus using 2D-SWE technology, high consistency was also observed. In this case, 5% (1/20) of the differences between the diaphragm elastic modulus measurements made by different physicians were outside of the consistency limit. The mean difference between measurements made by different physicians was 0.10 ± 1.61, with a 95% agreement limit ranging from -3.05 to 3.25. The intraclass correlation coefficient (ICC) was 0.775, with a 95% confidence interval (CI) of 0.511 to 0.905.
**Physiological and/or technical outcomes**	In patients with COPD, the correlation with the forced expiratory volume in one second (FEV1)/forced vital capacity (FVC), predicted FEV1% value, residual volume (RV), total lung capacity (TLC), RV/TLC, functional residual capacity

	(FRC) and inspiratory capacity (IC) of DS (R1=-0.81, R2=-0.63, R3= 0.65, R4= 0.54, R5= 0.60, R6= 0.72 and R7=-0.41, respectively; p < 0.001) was stronger than that of IMS (R1=-0.76, R2=-0.57, R3= 0.54, R4= 0.47, R5= 0.48, R6= 0.60 and R7=-0.33, respectively; p < 0.001).<br>The diaphragm elastic modulus was positively correlated with the intercostal muscle elastic modulus (r = 0.56, P < 0.001).
**Clinical outcomes**	In patients with COPD, diaphragm stiffness increased with disease severity (F = 224.50, p < 0.001). Significant differences in diaphragm elastic modulus measurements were not observed between the severe COPD group and the very severe COPD group. However, in the remaining groups, the elastic modulus of the diaphragm increased as the severity of COPD progressed.

**Author**	**Chino Y, 2018**
**Country**	Japan
**Study design**	Observational study, single-centre study
**Population**	- Healthy individuals<br>- Number of participants: 14<br>- Age: Mean 24.8 years (SD 5.0)<br>- Male/Female: All male
**New/advanced ultrasound approach and technical features**	**The innovative ultrasound technique used in this study is ultrasound shear wave elastography (SWE).**<br><br>• Timing of the assessment: Not applicable. Healthy individuals.<br>• Position of participant: Participants were seated on a reclined chair (at 75°).<br>• Type of breathing effort: Participants performed submaximal inspiratory tasks at various sustained target levels of inspiratory mouth pressure (15, 30, 45, 60, and 75% of MIP).<br>• Hemidiaphragm assessed: Right.<br>• Anatomical placement of the transducer/array: The transducer was placed on the eighth intercostal space on the right side, between the anteroaxillary and midaxillary lines (zone of apposition).<br>• Image acquisition procedure: Ultrasound shear wave elastography was performed using an Aixplorer ultrasound system (SuperSonic Imagine, Aix-en-Provence, France) with a 4-15 MHz linear array transducer (SL 15-4). The subject was seated in a reclined chair, with their right arm on a stand and their nose occluded with a nasal clamp. The transducer was adjusted to obtain clear echoes from the pleural and peritoneal membranes of the diaphragm and to place the region of interest (ROI) 0.5-2.0 cm below the costophrenic

	sinus. Shear wave elastography images were continuously acquired at 1 Hz during resting breathing, and the image at end expiration was selected for analysis.<br>• Image analysis procedure: The analysis was performed offline, where the average shear wave velocity was measured within a region of interest (ROI) and converted into shear modulus using the equation $\mu = \rho V^2$, where ρ is the density of soft tissues (1000 kg/m^3).<br>• Number of images analysed to retrieve results: A minimum of five and a maximum of eight images were analysed at each target inspiratory level, with the average value obtained from the three measurements with the lowest coefficient of variation (CV) used as the reference value.<br>• New Ultrasound markers: The primary ultrasound marker introduced was the diaphragm's shear modulus (μ) – mean value, measured in kilopascals (kPa) and derived from the shear wave velocity (V) as previously described. The average shear modulus at each target inspiratory level was expressed as a ratio relative to the shear modulus at resting end expiration – ratio value.
**Comparator(s)**	No comparator
**Reliability/Feasibility**	The intra-subject coefficient of variation (CV) for the diaphragmatic shear modulus measurements was less than 12% (at 0% MIP: 5.5%, at 15% MIP: 4.5%, at 30% MIP: 5.9%, at 45% MIP: 5.0%, at 60% MIP: 3.7%, at 75% MIP: 4.7%), which is considered acceptably low for biological measurements.
**Physiological and/or technical outcomes**	The analysis revealed significant differences in the relative ratio of shear modulus across the five inspiratory target levels ($p < 0.001$; $_p\eta^2 = 0.89$), with post-hoc comparisons showing all pairs differing significantly ($p \leq 0.007$; Cohen's d ranging from 0.54 to 3.39). When examining the relationship between inspiratory mouth pressure and shear modulus, both simple linear and second-order polynomial equations were fitted, yielding coefficients of determination (R^2) of 0.94 ± 0.05 for 8 out of 14 subjects ($R^2 \geq 0.95$) and 0.99 ± 0.01 for all subjects ($R^2 \geq 0.95$).
**Clinical outcomes**	N/a

**Author**	**Ciloglu O, 2020**
**Country**	Türkiye
**Study design**	Prospective case-control study, single-centre study
**Population**	- Type of population: Patients with hyperkyphosis due to osteoporotic vertebral fracture<br>- Number of participants: 78 (42 in the kyphosis group, 36 in the control group).

	- Age: Mean age of 81.10 ± 6.3 years (range: 67 to 93 years) for the kyphosis group and 81.00 ± 5.5 years (range: 69 to 97 years) for the control group.<br>- Male/Female: Kyphosis group - 14 males, 28 females; Control group - 11 males, 25 females.<br>- The kyphosis group consisted of patients diagnosed at a tertiary centre with osteoporotic vertebral fractures (defined as a >20% reduction in vertebral height) and hyperkyphosis, characterised by a Cobb angle of ≥50°.
**New/advanced ultrasound approach and technical features**	**The innovative ultrasound technique used in the study is strain elastography (SE).**<br><br>• Timing of the assessment: Not specified.<br>• Position of participant: Participants were positioned in a supine position during the ultrasound examinations.<br>• Type of breathing effort: Not specified but measured at end-expiration and end-inspiration.<br>• Hemidiaphragm assessed: Right.<br>• Anatomical placement of the transducer/array: The transducer was placed over the right chest wall between two costae in the anterior axillary line and moved caudally, with the patient in a supine position.<br>• Image acquisition procedure: A high-resolution ultrasound Doppler system (Philips® EPIQ 7) with a high-resolution linear probe (Philips® L5-18) was used for the ultrasound examinations. The diaphragm appeared as a hypoechoic structure located between the peritoneum and pleura. Elastographic images were captured using strain elastography (SE).<br>• Image analysis procedure: The analysis of the images was performed immediately by the same physician who was blinded to the patient groups. The ultrasound device automatically classified SE into three grades: Grade 1 for the hardest or hard tissue (red to yellow), Grade 2 for intermediate tissue (green), and Grade 3 for soft tissue (blue). The average of three consecutive measurements within a circular region of interest, taken at intervals of 1-3 mm, was considered. The ultrasound device also automatically calculated the strain ratio.<br>• Number of images analysed to retrieve results: The mean value of three consecutive measurements in a circular region of interest area at 1-3 mm intervals was taken into consideration.<br>• New Ultrasound markers: Strain (color code), strain ratio absolute values and percentage change at inspiration.
**Comparator(s)**	- Conventional ultrasound assessments of diaphragm thickness

	- Pulmonary function test (PFT) parameters such as Forced Expiratory Volume in the first second (FEV1) and Forced Vital Capacity (FVC)
**Reliability/Feasibility**	The intraclass correlation coefficient (ICC) for end inspirium and expirium strain ratio were calculated as 0.897 and 0.926, indicating good and excellent intra-observer reliability. The agreement for the color grades used in the strain elastography assessment was also very high, with Kendall's tau values of 0.984 (expirium) and 0.952 (inspirium) ($p < 0.001$).
**Physiological and/or technical outcomes**	Strain ratio values: The strain ratio values correlated inversely with the FEV1 (%) ($R = –0.929$, $p < 0.001$) and FVC (%) values ($R = –0.791$, $p < 0.001$).<br>Strain ratio values: Positively correlated with Cobb values ($R = 0.905$, $p < 0.001$) and number of vertebra fractures ($R = 0.782$, $p < 0.001$).
**Clinical outcomes**	Strain ratio values were significantly higher in the kyphosis group at end-inspiratory time (3.56 ± 0.1 vs. 2.88 ± 0.1, $p < 0.001$) and for percentage change (0.58 ± 0.1 vs. 0.27 ± 0.1, < 0.001). Rate of hardest colour code: Significantly higher in the control group ($p < 0.001$)..

**Author**	**Coiffard B, 2021**
**Country**	Canada
**Study design**	Observational study, multicentric study (2 centres)
**Population**	- Critically ill mechanically ventilated patients and healthy controls<br>- Number of participants: 34 patients (mechanically ventilated), 10 healthy controls<br>- Age: Mean 59 years (SD 16) for in the mechanically ventilated patient group, median age of 27 years [25–34 years] for the control group.<br>- Male/female: 10 (30%) in the mechanically ventilated patient group, 5 (50%) in the control group.<br>- Patients were enrolled within 36 hours of intubation. However, they were excluded if liberation from mechanical ventilation was anticipated within 24 hours or if they had undergone more than 48 hours of mechanical ventilation in the previous 6 months.<br>- Among comorbidities, COPD was present in 24%, asthma in 9%, interstitial lung disease (ILD) in 30%, obstructive sleep apnea (OSA) in 9%, chronic heart failure (CHF) in 6%, cirrhosis in 15%, chronic kidney disease (CKD) in 18%, and diabetes in 30%. Immunocompromised status was observed in 30% of the patients.<br>The Sequential Organ Failure Assessment (SOFA) score averaged 11 (SD: 3) over the first 72 hours, while the Simplified Acute Physiology Score (SAPS II) had a mean of 52 (SD: 15). The baseline PaO_2/FiO_2 ratio was a median of 159 mmHg, with an interquartile range (IQR) of 116–241 mmHg.

	Regarding cumulative fluid balance, patients had a median of 0.8 liters (IQR: -0.3–4.5) on Day 1 of ventilation, while the mean fluid balance on Day 3 was 4.1 liters (SD: 5.2).
**New/advanced ultrasound approach and technical features**	**The innovative ultrasound technique involves quantifying diaphragm echodensity using a grayscale histogram analysis.**<br><br>• Timing of the assessment: Daily, from ICU admission until day 5.<br>• Position of participant: not mentioned.<br>• Type of breathing effort: Quiet (end-expiration)<br>• Hemidiaphragm assessed: Right side (as noted in the article referenced by the authors, since this study is a secondary analysis).<br>• Anatomical placement of the transducer/array: in the zone of apposition between the anterior and midaxillary lines at the level of the 9th or 10th intercostal space (as noted in the article referenced by the authors, since this study is a secondary analysis).<br>• Image acquisition procedure: 13-MHz linear array transducer. B-mode ultrasound images with standardised gain and frequency settings; three different machines used (Phillips Sparq, Mindray, Fujifilm Sonosite), same machine per patient.<br>• Image analysis procedure: Image analysis was performed offline using ImageJ software, where grayscale histogram analysis was conducted to quantify echodensity. The analysis involved selecting a free-form area (trace method) devoid of artifacts between the pleural and peritoneal membranes.<br>• Image analysis procedure: Offline analysis using ImageJ software; grayscale histogram analysis with ED50 and ED85 percentile, as well as the percentage of pixels above a grayscale value of 65 (HEA65), which was determined to be the upper limit of normal based on the healthy control group; trace method to select area between pleural and peritoneal membranes.<br>• Number of images analysed for results: Not explicitly stated. A total of 104 images from 34 patients were analysed.<br>• New Ultrasound markers: the ED50 (50th percentile of grayscale values), ED85 (85th percentile of grayscale values) and HEA65 (percentage of pixels above grayscale value of 65). which were used to quantify diaphragm echodensity. These markers were expressed in grayscale units ranging from 0 (black) to 255 (white) or percentage of pixel (for HEA65).
**Comparator(s)**	1) Diaphragm echodensity (ED50, ED85 and HEA65) in mechanically ventilated patients compared to healthy control subjects.<br>2) Changes in diaphragm echodensity over time in mechanically ventilated patients and how they relate to clinical outcomes such as ventilator-free days.

**Reliability/Feasibility**	The reproducibility of echogenicity between analyzers (measured on one image by two different analyzers) showed an average difference of -1.5, with limits of -8.6 to 5.7, based on 30 images. The reproducibility of echogenicity between images (measured on two separate images taken from the same patient on the same day by a single analyzer) demonstrated an average difference of -2.8, with limits of -15.8 to 10.2, also based on 30 images. The reproducibility of echodensity at end-expiration and end-inspiration (two measurements taken during the same respiratory cycle by a single analyzer) indicated an average difference of -1.3, with limits of -9.8 to 7.2, based on 15 images.
**Physiological and/or technical outcomes**	Both increases and decreases in diaphragm thickness from baseline were linked to increases in ED50 over time (p = 0.03, within-subjects R^2 = 0.78). This was maintained even after adjusting for cumulative fluid balance on day 3 (adjusted p = 0.03, within-subjects R^2 = 0.80).<br>The relationship between baseline ED50 and cumulative fluid balance was evaluated. On Day 1 of ventilation, the cumulative fluid balance had a correlation coefficient (CC) of -0.2 (95% CI: −1.4 to 1.0, p = 0.72). By Day 3 of ventilation, the cumulative fluid balance showed a CC of 1.2 (95% CI: −1 to 3, p = 0.24).<br>No significant differences were observed in cumulative fluid balance on Day 1 between patients with a ≤ 10-point change in ED50 and those with a > 10-point increase in ED50 (2.1 L [IQR: 0.1–5.1] vs. 0.3 L [IQR: 0.7–0.9], respectively, p = 0.36). Similarly, no differences were found on Day 3 cumulative fluid balance (3.7 ± 6.2 L vs. 4.6 ± 3.5 L, respectively, p = 0.60).
**Clinical outcomes**	At baseline, mechanically ventilated patients exhibited higher diaphragm echodensity compared to healthy subjects, with median ED50 values of 27 (IQR 23–34) versus 40 (IQR 23–55, p = 0.07) and median ED85 values of 39 (IQR 36–52) versus 56 (IQR 42–84, p = 0.04). Additionally, 53% of patients had abnormally increased echodensity (HEA65 > 5%). Baseline ED50 was not associated with patient characteristics or clinical outcomes, including ventilator-free days to day 60, duration of ICU admission, or mortality.<br>The evolution of diaphragm echodensity varied widely among patients during mechanical ventilation. Increased echodensity (defined as a >10-point increase in ED50) developed in 13 patients (38%) at a median of day 3 after intubation (IQR [2–4]), with a median maximal increase of +18 points (IQR +16 to +26 points) over the first 5 days. There was no association between increased echodensity and patient characteristics, including severity of illness score or cumulative fluid balance.<br>Patients with increased echodensity had fewer ventilator-free days to day 60 (median 46, IQR 0–52, vs. median 53, IQR 49–56, p = 0.03) and were more likely to require ventilation for ≥ 7 days (85% vs. 33%, p = 0.01). There was a trend towards higher mortality in patients with increased ED50 (38% vs. 10%, p = 0.11).

	Patients requiring mechanical ventilation for more than 7 days showed significant increases in ED50 on day 2 (+40% from baseline, IQR +4% to +54%) compared to those needing ventilation for less than 7 days (−9% change, IQR −45% to +4%, p = 0.007). This association persisted after adjusting for changes in diaphragm thickness (adjusted p = 0.01).<br>In a sensitivity analysis of patients ventilated for at least 3 days (n = 33), increased ED50 within 3 days (n = 8, 24%) was associated with fewer ventilator-free days compared to those with unchanged echodensity (median 48 days, IQR [0–50] vs. median 52 days, IQR [44–56], p = 0.09). Among these patients, 88% with increased ED50 required prolonged ventilation (≥ 7 days), compared to 44% of those without an increase (p = 0.08).

**Author**	**Da Conceicao D, 2024**
**Country**	Canada
**Study design**	Prospective observational study, single-centre study
**Population**	- Elective surgical patients with normal diaphragmatic function<br>- Number of participants: 75<br>- Age: mean 53.1 years (SD 16.5)<br>- Male/Female ratio: 1.9:1<br>- Exclusion criteria were abnormal pulmonary function tests, known pre-existing diaphragmatic dysfunction, or body mass index>35 kg/m^2
**New/advanced ultrasound approach and technical features**	**The innovative ultrasound technique is measuring the excursion of the zone of apposition (ZOA).**<br><br>• Timing: Not specified, likely before surgery.<br>• Position of participant: Patients were positioned in a semi-recumbent or upright position to facilitate optimal imaging of the diaphragm.<br>• Type of breathing effort: The assessment was conducted during deep breathing efforts to capture the full range of diaphragmatic motion.<br>• Hemidiaphragm assessed: Both right and left sides.<br>• Anatomical placement of the transducer/array: The transducer was placed in the mid-axillary line to target the zone of apposition effectively.<br>• Image acquisition procedure: Assessment of the excursion of the ZOA using a linear 10-12 MHz probe, with skin marks made at the most cephalad point of the ZOA in end-inspiration and end-expiration, and the distance between the marks measured.<br>• Image analysis procedure: Image analysis was performed immediately after acquisition, with the assessment focusing on the excursion of the diaphragm

	during deep breathing. The analysis included measuring the distance of diaphragm movement from full expiration to full inspiration with a ruler.<br>• Number of images analysed to retrieve results: For both excursion methods, the measurement was performed twice, and the average of the two values will be used. No information available for thickening fraction assessment.<br>• Ultrasound markers: Excursion of the zone of apposition (ZOA), measured in millimeters (mm). Calculations included the difference in diaphragm position from expiration to inspiration to quantify movement.
**Comparator(s)**	- Assessment of the excursion of the dome of the diaphragm (DOD).<br>- Assessment of the thickening fraction at the zone of apposition (ZOA).
**Reliability/Feasibility**	The evaluation of the excursion of the ZOA was found to be consistently successful (100% bilaterally) compared to the evaluation of the excursion of the DOD (98.7% on the right side and 34.7% on the left side).
**Physiological and/or technical outcomes**	- The excursion of the diaphragm's dome of the diaphragm (DOD) between the right and left sides showed a significant correlation (Spearman correlation coefficient (CC) of 0.57, $p<0.01$), as did the measurements of excursion of the zone of apposition (ZOA) on both sides (Spearman CC 0.62, $p<0.001$). Additionally, there was a significant correlation between the ZOA and DOD measurements on both sides (Spearman CC 0.28 on the right side and 0.42 on the left side, $p<0.05$).<br>- The data for the thickening fraction exhibited much greater variability, and no significant correlation was found between the right and left sides, nor between the thickening fraction and any method used to assess diaphragmatic excursion.
**Clinical outcomes**	N/a

**Author**	**Fayssoil A, 2019**
**Country**	France
**Study design**	Retrospective observational study, single-centre study
**Population**	- Patients with genetically confirmed neuromuscular diseases and healthy adult individuals (control group)<br>- Number of participants: total of 116 (89 in patient group and 27 in control group). For patient group:<br>• 35% Duchenne muscular dystrophy (DMD),<br>• 29% myotonic dystrophy type 1,<br>• 12% sarcoglycanopathies,<br>• Other diseases include mitochondrial disease, congenital myopathy, spinal muscular atrophy, Pompe disease, desminopathy.<br>• Overall, 63% of patients were on home mechanical ventilation.

	- Age: Median 32 years [IQR 25; 50] in patient groups, Median 31 years [IQR 26; 39] in control group.<br>- Male/Female: 11 (12%) in patient group, 14 (50%) in control group.
**New/advanced ultrasound approach and technical features**	**The innovative ultrasound technique used in this study is the diaphragm ultrasound using tissue Doppler imaging (TDI).**<br><br>• Timing of the assessment: The assessment was performed during routine follow-up visits for patients with neuromuscular disorders.<br>• Position of participant: Participants were positioned in a semi-recumbent position (45°) during the ultrasound procedure.<br>• Type of breathing effort: The assessment utilised a sniff manoeuvre to evaluate diaphragm motion and function.<br>• Hemidiaphragm assessed: Both the right and left hemidiaphragms were assessed.<br>• Anatomical placement of the transducer/array: The transducer was placed in the anterior subcostal region between the midclavicular and anterior axillary lines to visualize the diaphragm.<br>• Image acquisition procedure: A cardiac probe was used, with the tissue Doppler imaging modality activated and the beam positioned perpendicularly to the diaphragm motion, using the liver window for the right hemidiaphragm and the spleen window for the left hemidiaphragm. The peak sniff inspiratory velocity was recorded in centimeters per second (cm/s).<br>• Image analysis procedure: this information is not specified in the document.<br>• Number of images analysed to retrieve results: Multiple images were analysed, although the exact number is not specified in the document.<br>• New Ultrasound markers: The assessment retrieved new marker such as peak TDI velocity (measured in cm/s) during sniff manoeuvre.
**Comparator(s)**	The comparators in this study were sniff nasal pressure, forced vital capacity (FVC), Sniff diaphragm motion using time movement (TM) ultrasound.
**Reliability/Feasibility**	N/a
**Physiological and/or technical outcomes**	The right sniff peak TDI velocity was significantly correlated with forced vital capacity (FVC) ($r = 0.72$, $p < 0.0001$) and with sniff nasal pressure ($r = 0.66$, $p < 0.0001$). Similarly, supine FVC was significantly correlated with right diaphragm sniff TDI velocity ($r = 0.59$, $p = 0.0007$, $n = 29$). This relationship remained consistent across different neuromuscular disorders, including Duchenne muscular dystrophy, myotonic dystrophy type 1, and other myopathies.
**Clinical outcomes**	Among patients with preserved respiratory function (FVC > 80%), three out of five (60%) showed right sniff TDI peak velocities below the control median.

	In predicting respiratory muscle impairment in muscular dystrophy, sniff diaphragm ultrasound demonstrated high diagnostic accuracy. To predict FVC < 60%, a right peak TDI velocity cutoff of 7.5 cm/s achieved a sensitivity of 84% and a specificity of 89%, while a right TM motion cutoff of 25 mm reached a sensitivity of 100% and a specificity of 64%. The area under the curve (AUC) for these parameters was 0.93 (p < 0.0001) for sniff right TM mode and 0.86 (p < 0.001) for right peak diaphragm TDI velocity.<br>In patients with more severe respiratory impairment (FVC < 30%), the right peak TDI velocity cutoff decreased to 6.5 cm/s, with a sensitivity of 90% and a specificity of 56%, while the right TM motion cutoff of 10.5 mm provided both sensitivity and specificity of 84%. In this population, the AUC for right diaphragm ultrasound TDI remained high but slightly decreased to 0.76 (p < 0.017).

**Author**	**Fjaellegaard K, 2024**
**Country**	Denmark
**Study design**	Prospective observation study, single centre
**Population**	- Type of Population: Patients with unilateral pleural effusion, primarily malignant in nature.<br>- Number of Participants: 104 patients were included in the study. “Responders” were identified by an improvement of minimal importance defined as a decrease of ≥ 1 on Modified Borg Scale, 10 minutes after drainage.<br>- Age: The mean age of participants was 73 years (SD 10).<br>- Male/Female: Male: 60 (58%), Female: 44 (42%)<br>- Detailed Information about Patients' Disease:<br>o Aetiology of Pleural Effusion<br>o Malignant: 75 (72%): Lung: 37 (36%), Breast: 12 (12%), Mesothelioma: 12 (10%), Ovarian: 3 (3%), Lymphoma: 4 (4%), Uterus: 1 (1%), Sarcoma: 1 (1%), Kidney: 2 (2%), GIST: 1 (1%), Mb. Waldenström: 1 (1%), Unknown primary tumour: 1 (1%).<br>o Non-malignant: 29 (28%) Cardiac failure: 10 (10%), Pleuritis: 7 (7%), Hepatic insufficiency: 2 (2%), Pulmonary embolism: 1 (1%), Pulmonary hypertension: 1 (1%), Kidney failure and pulmonary hypertension: 1 (1%), Complicated parapneumonic effusion: 2 (2%), Post CABG: 1 (1%), Meig’s syndrome: 1 (1%), Unknown: 3 (3%).<br>o Comorbidities: 50 (48%) of the participants had comorbidities associated with dyspnoea.<br>o Prior Thoracenteses:

	o Median number of thoracenteses within one year: 3 (IQR 2-5).<br>o Median days since most recent thoracentesis: 20 (IQR 11-42).<br>o Baseline Dyspnoea: The median Modified Medical Research Council (MRC) dyspnoea score was 4 (IQR 2-5).
**New/advanced ultrasound approach and technical features**	**The innovative ultrasound technique is the "Area method," which assesses diaphragm movement in two dimensions (cranio-caudal and posterior-anterior).**<br><br>• Timing of the assessment: The ultrasound assessments were performed before and immediately after drainage during therapeutic thoracentesis.<br>• Position of patient: Patients were placed in an erect position during the ultrasound evaluations.<br>• Type of breathing effort: calm and unforced breathing.<br>• Hemidiaphragm assessed: Both hemidiaphragms were evaluated during the ultrasound assessments. It is implied that the analysis focused on the hemidiaphragm corresponding to the side of the thoracentesis.<br>• Anatomical placement of the transducer/array: Lateral view in the mid-axillary line to visualize the diaphragm dome.<br>• Image acquisition procedure:<br>o Equipment used: LOGIQ S8 (GE Healthcare, Wauwatosa, USA) and ALOKA ARIETTA V60 (Hitachi, Tokyo, Japan).<br>o Type of transducer: A C1–5 curved abdominal transducer (2–5 MHz) or a C42 micro-convex transducer (4–8 MHz) were utilised, with abdominal and liver presets, respectively. All US film clips were recorded for further analysis.<br>• Image analysis procedure:<br>o Offline analysis by identifying frames of maximal inspiration and expiration, measuring the area above the diaphragm dome by tracing the visual part of the diaphragm.<br>• Number of images analysed to retrieve results: Multiple film clips were recorded, with at least two images per respiration cycle (maximal inspiration and expiration) analysed.<br>• New Ultrasound markers: Area Change: Measured in square centimetres (cm^2), calculated by tracking the curve of the hemidiaphragm.
**Comparator(s)**	Conventional ultrasound assessments of diaphragm movement (e.g., M-mode). Clinical values related to dyspnoea improvement (e.g., modified Borg scale scores).
**Reliability/Feasibility**	N/a

**Physiological and/or technical outcomes**	When comparing diaphragm movement measured by the Area method with excursion measured by M-mode, both before and after thoracentesis at the site of effusion and on the opposing side, very low but statistically significant correlation coefficients were observed between the two techniques, with the highest coefficient of 0.06
**Clinical outcomes**	No association was found between baseline diaphragm movement, measured by the Area method, and being a responder (Odds ratio, 1.04, 95% CI 0.97–1.11, $p = 0.25$).<br>Changes in Diaphragm Movement Following Thoracentesis:<br>At the site of effusion, diaphragm movement increased significantly after thoracentesis when measured by the Area method: Before Drainage: Mean (SD) = 8.6 cm^2 (6.2) After Drainage: Mean (SD) = 14.6 cm^2 (9.7), Mean Delta: 6.5 cm^2 (SD 9.4), $p < 0.001$<br>Opposing Side Measurements: No significant change in diaphragm movement was observed on the opposing side using the Area method: Mean Delta: -0.3 cm^2 (SD 8.7), $p = 0.79$

**Author**	**Flatres A, 2020**
**Country**	France
**Study design**	Prospective observational study, single-centre study
**Population**	- Healthy subjects and critically ill patients.<br>- Number of participants: total of 43 (31 adult healthy subjects and 12 consecutive adult critically ill patients).<br>- Healthy subjects:<br>• Age: 26.7 ± 4.6 years<br>• Male: 54.8%<br>- Critically ill patients:<br>• Age: 66.6 ± 20.9 years<br>• Male: 58%<br>• Admitted for various reasons such as respiratory distress (57%), coma (17%), sepsis (17%), and acute kidney injury (8%).<br>• Severity, SOFA score: 8.1 ± 6.2Included if expected length of ICU stay of at least 3 days.<br>• Clinical management included the use of continuous neuromuscular blockade infusion (42%), systemic steroids (42%), duration of sedation (median 4 days), and duration of invasive mechanical ventilation (median 5.8 days).

**New/advanced ultrasound approach and technical features**	**Ultrasound shear wave elastography (SWE) to assess diaphragm stiffness.**<br>• Timing of the assessment: Not defined. During ICU stay.<br>• Position of participant: Healthy individuals were positioned in a supine and critically ill patients in semi-recumbent position.<br>• Type of breathing effort: Healthy controls - Quiet breathing with end-expiratory breath hold; Mechanically ventilated patients - End-expiratory pause<br>• Hemidiaphragm assessed: Right.<br>• Anatomical placement of the transducer/array: The transducer was placed at the zone of apposition at the 8th–10th intercostal space between the right anterior and midaxillary lines.<br>• Image acquisition procedure: SWE images acquired continuously at 2 Hz using a high-frequency ultrasound machine (Aixplorer ultrasonic scanner,SuperSonic Imagine, Aix-en-Provence, France) with a linear array transducer (SL15-4; SuperSonic Imagine) in SWE mode with musculoskeletal pre-set. Conscious subjects were instructed to stay relaxed, breathe quietly throughout the procedure, and hold their breath at functional residual capacity during SWE acquisition. For mechanically ventilated patients, an end-expiratory pause was used, and diaphragm inactivity was confirmed through ventilator curves and real-time ultrasound imaging. To ensure consistent ultrasound assessments, marks were placed on the subject's chest during the initial measurement and used by both operators for subsequent evaluations. During image acquisition, transducers were applied with minimal pressure over a generous amount of coupling gel to prevent tissue distortion.<br>• Image analysis procedure: Offline manual drawing of the widest region of interest (Q-box trace) on a frozen image to average the shear modulus measurement.<br>• Number of images analysed to obtain results: Images were collected in triplicate. A total of 190 images were evaluated for the diaphragm shear modulus. Specifically, for critically ill patients (n = 12), 72 images were obtained by two assessors.<br>• New Ultrasound markers: The new ultrasound marker retrieved was the diaphragm shear modulus, measured in kilopascals (kPa). The shear modulus was calculated based on the shear wave velocity obtained during the assessment.
**Comparator(s)**	No comparator
**Reliability/Feasibility**	**Training Set (Healthy Subjects, n=16)**<br>Longitudinal view (transducer being positioned parallel to the fibres):<br>Mean (SD) Operator 1: 19.4 (6.2) kPa; Mean (SD) Operator 2: 20.1 (7) kPa

	Inter-operator reproducibility (ICC): 0.83 [0.50 to 0.94]<br>Transverse view (transducer being positioned perpendicular to the fibres):<br>Mean (SD) Operator 1: 25.4 (7.3) kPa; Mean (SD) Operator 2: 22.4 (6.3) kPa<br>Inter-operator reproducibility (ICC): 0.3 [−0.86 to 0.75]<br><br>**Validation Set (Healthy Subjects, n=15)**<br>Mean (SD) Operator 1: 20 (7.3) kPa; Mean (SD) Operator 2: 20.6 (6.1) kPa<br>Inter-operator reproducibility (ICC): 0.96 [0.85–0.99]<br>Intra-operator 1 reliability (ICC): 0.95 [0.82–0.99]<br>Intra-operator 2 reliability (ICC): 0.90 [0.70–0.98]<br><br>**Critically Ill Patients (n=12)**<br>Mean (SD) Operator 1: 13.1 (4.2) kPa; Mean (SD) Operator 2: 14.2 (4.6) kPa<br>Inter-operator reproducibility (ICC): 0.92 [0.71–0.98]<br>Intra-operator 1 reliability (ICC): 0.93 [0.82–0.98]<br>Intra-operator 2 reliability (ICC): 0.92 [0.81–0.98]<br><br>The ICC values for the validation set and critically ill patients are all above 0.9, which is classified as "excellent agreement," demonstrating the reliability of SWE.
**Physiological and/or technical outcomes**	N/a
**Clinical outcomes**	N/a

**Author**	**Formenti P, 2022**
**Country**	Italy
**Study design**	Prospective observational study, single-centre study
**Population**	- Intubated ICU patients with confirmed SARS COV 2 infection and ARDS<br>- Number of participants: 32<br>- Age: 63.9 ± 7.4 years<br>- Male/female: 25 males (78%), 7 females (22%).<br>- Severity based on ICU scores: SAPS II – 32 ± 10 points and SOFA – 6.3 ± 2.4 points.<br>- Symptoms began 10.3 ± 7 days before hospital admission, with intubation occurring 12.5 ± 6 days after symptom onset. During this interval, the median duration of the NIV trial was 2.2 ± 2.5 days. The ICU mortality rate was 53%.

**New/advanced ultrasound approach and technical features**	**The innovative ultrasound technique used in this study is the assessment of diaphragm echogenicity using greyscale analysis.**<br><br>• Timing of the assessment: The ultrasound assessment was performed at the time of ICU admission (within 24h).<br>• Position of participant: The participant was positioned at a 45° angle during the assessment.<br>• Type of breathing effort: The assessment was conducted during quiet breathing, as all deeply sedated and mechanically ventilated at ICU admission.<br>• Hemidiaphragm assessed: Right.<br>• Anatomical placement of the transducer/array: The transducer was placed above the right 10th rib in the mid-axillary line to assess diaphragm in the zone of apposition.<br>• Image acquisition procedure: B-mode images were obtained using a 6–14 MHz linear array on a Mindray TE-7 machine (Shenzhen Mindray Bio-Medical Electronics Co. Ltd. Shenzen, China). Depth and gain were standardised using the same image presets between patients.<br>• Image analysis procedure: Images were stored in JPEG format and were analysed offline using ImageJ software, where greyscale histogram analysis was performed to quantify echogenicity (https://imagej.nih.gov/ij/index.html; NIH, Bethesda, MD, USA). The analysis involved selecting a region of interest devoid of artifacts (square method).<br>• Number of images analysed to obtain results: Not specified. However, the authors indicate that they utilised an average of three consecutive measurements within a 10% range for a different assessment (cross-sectional area of the rectus femoris).<br>• New Ultrasound markers: Diaphragm echogenicity, quantified using a grayscale score (arbitrary units). The analysis involved calculating the mean pixel intensity within the defined region of interest.
**Comparator(s)**	In this study, diaphragm echogenicity was compared between ICU survivors and non-survivors. Additionally, its association with right intercostal echogenicity, fluid balance and urinary protein output was evaluated.
**Reliability/Feasibility**	N/a
**Physiological and/or technical outcomes**	Significant correlation between diaphragm echogenicity and right parasternal intercostal muscle echogenicity ($R^2 = 0.3225$, $p = 0.001$).<br>A significant correlation was found between diaphragm echogenicity and cumulative fluid balance ($R^2 = 0.315$, $p = 0.001$).

	A significant correlation was found between diaphragm echogenicity and urinary protein output ($R^2 = 0.436$, $p < 0.001$).
**Clinical outcomes**	Echogenicity of the diaphragm was significantly different between survivors and non-survivors, measuring median 65 [IQR, 62.6-68] AU for survivors and median 77 [IQRm 74.2-94] AU for non-survivors ($p = 0.0002$).

**Author**	Fossé Q, 2020
**Country**	France
**Study design**	Prospective observational study, single-centre study
**Population**	- Critically ill patients in ICU, mechanically ventilated, readiness-to-wean criteria met.<br>- Number of participants: 30, 25 were considered for the analysis.<br>- Age: Median 65 years (range 58–75).<br>- Male/Female: 19 males (76%), 6 females (24%).<br>• COPD: 9 patients (38%)<br>• Chronic cardiac disease: 12 patients (50%)<br>• Chronic kidney disease: 5 patients (21%)<br>• ICU scores: SAPS 2 score median 51 (range 39–62), SOFA score median 5 (range 4–8).<br>• Duration of intubation: Median 4 days (range 3–7).
**New/advanced ultrasound approach and technical features**	**Ultrasound shear wave elastography (SWE) to assess diaphragm stiffness.**<br><br>• Timing of the assessment: Conducted during the ICU stay, taken under various conditions of pressure support ventilation and during a spontaneous breathing trial (SBT).<br>• Position of participant: Participants were positioned in semi-recumbent position.<br>• Type of breathing effort: Quiet breathing under various ventilator settings, as well as during an SBT. Each patient experienced four consecutive mechanical ventilation conditions in a randomised order: Baseline Pressure Support (PS), which involved initial settings determined by the physician; Increased Pressure Support (PS +25%), where pressure support was increased by 25% while maintaining baseline PEEP; Decreased Pressure Support (PS -25%), which involved a 25% decrease in pressure support while keeping baseline PEEP constant; and Zero End-Expiratory Pressure (PS ZEEP), where baseline pressure support was applied with zero end-expiratory pressure. Each condition lasted for 10 minutes, with data acquisitions performed at 3 and 9 minutes. Following these conditions, every patient underwent a planned 30-

	minute spontaneous breathing trial (SBT) during which no assistance was provided from the ventilator, with pressure support and PEEP set at 0 cmH_2O. This modality of SBT, part of the usual care in the research ICU, reflected the work of breathing after extubation. Thirty-second ultrasound and pressure recordings were taken at the onset of the SBT and every five minutes for a maximum of 30 minutes.<br>• Hemidiaphragm assessed: Right.<br>• Anatomical placement of the transducer/array: The probe was placed on the mid-axillary line, vertical to the chest wall, at the 8th-11th intercostal space to image the right hemidiaphragm.<br>• Image acquisition procedure: An ultrafast ultrasound scanner (Aixplorer, SuperSonic Imagine) with a linear transducer array (SL 10-2) was used to capture images. The built-in SWE mode of the ultrasound scanner was used, with a sampling rate of 1.6-2 Hz and simultaneous acquisition of B-mode images at 12 Hz.<br>• Image analysis procedure: images were analysed offline using standardised MATLAB (Mathworks, Natick, MA, USA) scripts. A rectangular region of interest (ROI) was manually drawn in the centre of the diaphragm on the first frame of each recording and then replicated in subsequent frames. The diaphragm shear modulus was calculated using the formula $SMdi = \rho \cdot Vs^2$, where Vs represents the velocity of the shear wave and ρ is the muscle density (1000 kg/m^3). The SMdi was reported as the median shear modulus within each ROI.<br>• Number of images analysed to retrieve results: For each ventilatory condition, the three cycles with the least variation in ΔPdi were deemed representative of that condition and chosen for further analysis.<br>• New Ultrasound markers: ΔSMdi, inspiratory change in diaphragm shear modulus assessed (SMdi) using ultrasound shear wave elastography, in kPa.
**Comparator(s)**	Changes in transdiaphragmatic pressure (ΔPdi) measured using a double-balloon feeding catheter, changes in diaphragm function in response to varying ventilator settings, changes in diaphragm function during a spontaneous breathing trial.
**Reliability/Feasibility**	N/a
**Physiological and/or technical outcomes**	A significant correlation was observed between ΔPdi and ΔSMdi ($R = 0.45$, 95% CIs [0.35, 0.54], $p < 0.001$). Individual correlations showed a significant relationship in 8 out of the 25 patients ($r = 0.55 – 0.86$, all $p < 0.05$), compared to a non-significant range of $r = −0.43 – 0.52$ (all $p > 0.06$). Altering the ventilation conditions similarly influenced both ΔPdi and ΔSMdi. Patients with a non-significant ΔPdi–ΔSMdi correlation exhibited a faster respiratory rate compared to those with a significant correlation (median (Q1–Q3), 25 (18–33) vs. 21 (15–26)

	breaths.min−1, respectively). No difference in BMI was found between patients with and without a significant ΔPdi–ΔSMdi correlation
**Clinical outcomes**	Likewise, both PTPdi and ΔPdi showed a significant increase as the level of assistance decreased. Under pressure support (PS) ventilatory conditions, ΔPdi varied from 0.1 to 38.1 cmH2O, and from 0.6 to 50.7 cmH2O during the spontaneous breathing trial (SBT). Similarly, ΔSMdi exhibited a gradual increase corresponding to each reduction in the level of ventilatory assistance. The ΔSMdi values (in kPa) at various ventilatory conditions were as follows: PS+25% (median 5.5, IQR 3.8–9.0), PS (median 5.4, IQR 3.5–8.8), PS-25% (median 7.0, IQR 5.8–8.6), PSZEEP (median 7.7, IQR 4.0–11.8), SBT Start (median 12.2, IQR 7.7–14.3), and SBT End (median 7.5, IQR 4.8–13.1). Significant differences were noted between several conditions: SBT Start was significantly different from PS+25%, PS, PS-25%, and PSZEEP, while SBT End was significantly different from PS+25%, PS, and SBT Start. All comparisons had p-values less than 0.05.<br><br>Thirteen patients (52%) failed in the spontaneous breathing trial (SBT). The reasons for failure included acute respiratory distress (5 out of 13), neurological impairment (4 out of 13), and weaning-induced pulmonary edema (4 out of 13). ΔPdi and PTPdi were generally higher at the beginning of the SBT in patients who failed compared to those who succeeded.

**Author**	**Fritsch SJ, 2022**
**Country**	Germany
**Study design**	Observational study, single-centre study
**Population**	- Patients undergoing elective coronary artery bypass graft (CABG) surgery<br>- Number of participants: 20<br>- Age: Mean age of 63.9 ± 7.3 years (range 52 to 81 years)<br>- Male/female: 18 (90%) males and 2 females<br>- Detailed information about patients' diseases: All had coronary heart disease, Atrial fibrillation in 5 patients (25%), Arterial hypertension in 16 patients (80%), Chronic obstructive pulmonary disease in 3 patients (15%), Obstructive sleep apnoea syndrome in 2 patients (10%)<br>- Duration of mechanical ventilation (OR+ICU) (hh:mm): Mean duration of 16:40±6:23
**New/advanced ultrasound approach and technical features**	**Speckle tracking ultrasound to assess diaphragm contractility by measuring strain and strain rate.**

	• Timing of the assessment: Preoperatively, within 24 hours, and within 48 hours after extubation.<br>• Position of participant: Participants were placed in a 30° upright position.<br>• Type of breathing effort: Quiet/tidal breathing and voluntary maximal inspiratory maneuver.<br>• Hemidiaphragm assessed: Right.<br>• Anatomical placement of the transducer/array: The transducer was positioned in the zone of apposition (8th to 11th intercostal spaces) of the right hemithorax between the anterior axillary and mid-axillary line, longitudinal to the body axis, with the diaphragm identified as the layer between the pleural and peritoneal echogenic lines.<br>• Image acquisition procedure: Ultrasound was performed using a 9-MHz linear transducer on a Vivid E 9 ultrasound device (General Electric Healthcare, Horten, Norway). The penetration depth and focus were optimally adjusted, and respiratory curves obtained from electrocardiography were combined with the ultrasound images. The cineloops were saved and analysed offline.<br>• Image analysis procedure: The Q-analysis tool in EchoPac software (version 113, revision 1.0; General Electric Healthcare, Boston, MA, USA) was used to analyze ultrasound data with the speckle tracking technique in 2D strain mode. At least three loops were included for basal respiration, while all analyzable loops were used for forced inspiration. Each loop covered a full breathing cycle, and a region of interest (ROI) was manually defined on a representative part of the diaphragm. The software calculated the inspiratory peak strain and peak strain rate for longitudinal deformation.<br>• Number of images analysed to retrieve results: Specific number not provided in the document.<br>• New Ultrasound markers: The innovative ultrasound markers identified in this study are "strain" and "strain rate". Strain indicates the relative change in length compared to an initial reference state (%), whereas strain rate reflects the speed of deformation (1/s) and is independent of the reference state. In both instances, a greater degree of deformation and velocity is associated with a more negative parameter value.
**Comparator(s)**	Diaphragm speckle tracking was compared with thickening fraction.
**Reliability/Feasibility**	N/a
**Physiological and/or technical outcomes**	N/a<br>There were no clear correlations between strain, strain rate, and FT (no data available).

**Clinical outcomes**	The ultrasonographic assessment of the diaphragm revealed significant findings. Within 24 hours after extubation, all patients experienced a decrease in strain during basal respiration, with median strain dropping to 73% of preoperative values. By 48 hours post-extubation, strain values returned close to preoperative levels, with both the decrease from the first to the second assessment and the increase from the second to the third being statistically significant (P < 0.001). In terms of strain rate under basal respiration, the median strain rate remained largely unchanged postoperatively, although some patients exhibited significant fluctuations. Notably, the median strain rate increased significantly 48 hours after extubation, with all but one patient recovering to preoperative levels or exceeding them, including two patients with more than a threefold increase. This increase was statistically significant compared to preoperative values (-0.41, IQR 0.27, vs. -0.58, IQR 0.25, p = 0.010) and the first postoperative assessment (-0.39, IQR 0.25, vs. -0.58, IQR 0.25, p < 0.001).<br>For strain under forced inspiration, only 14 patients were analysed due to poor image quality. Most showed a similar pattern of early postoperative decrease followed by recovery, akin to basal respiration. However, some patients did not recover and continued to exhibit decreased strain 48 hours after extubation. Additionally, half of the examined patients had a decreased strain rate within 24 hours after extubation, while the other half showed no changes or increased values. From the second to the third examination, the strain rate did not exhibit significant changes in the majority of patients. Notably, those with an increased strain rate postoperatively continued to show this deformation in velocity over time. No correlation was found between strain or strain rate and various factors such as age, body mass index, length of mechanical ventilation, cardiac function, or pre-existing diseases. However, a negative correlation was observed between diaphragm tissue deformation, strain and strain rate, and the volume of fluid administered within the first 24 hours in the ICU. Specifically, at 48 hours post-extubation, the Spearman correlation coefficient for strain and fluid volume was -0.531, while for strain rate, it was -0.495, both statistically significant (P = 0.023 for strain and P = 0.037 for strain rate). A similar but non-significant effect was noted 24 hours earlier.

**Author**	**Fu X, 2022**
**Country**	China
**Study design**	Prospective observational study, Single-centre study

**Population**	- Type of population: Patients undergoing major abdominal surgery. Patients were divided into two groups based on the occurrence of postoperative pulmonary complications (PPCs).<br>- Number of participants: 117<br>- Age: mean 57.57 (± 15.46) years, PCs group average age 60.64 (± 16.53) years; non-PPCs group average age 54.41 (± 13.93) years<br>- Male/female: 78 (66.67%) males<br>- The cohort's severity classification, based on the ASA scale, was predominantly ASA grade 2 (47.01%) and 3 (47.86%), with 5.13% classified as grade 4. The APACHE II score, used to assess severity of illness, had an average of 13.29 ± 7.39.<br>In terms of comorbidities, cardiovascular disease was present in 17.09% of the cohort, diabetes mellitus in 7.69%, COPD in 3.42%, hyperlipidemia in 4.27%, and hypertension in 14.53%. Chronic renal disease affected 32.48% of patients, and chronic liver disease was noted in 41.03%. Additionally, 41.88% of the cohort had active cancer.<br>ICU and hospital stay lengths were 6.48 ± 6.21 days and 17.37 ± 15.49 days, respectively. Ventilator-free days at 28 (VFD-28) averaged 25.35 ± 3.78 days. Mortality rate was 5.13%, and 5.98% of patients required reintubation. After extubation, the use of ventilatory support varied: 58.12% of patients required conventional oxygen therapy (COT), 11.86% used high-flow nasal cannula (HFNC), and 29.66% were supported with non-invasive positive pressure ventilation (NIPPV).
**New/advanced ultrasound approach and technical features**	**The innovative ultrasound technique used in the study is diaphragm echodensity measurement.**<br><br>• Timing of the assessment: The assessment was performed during spontaneous breathing trials (SBT).<br>• Position of participant: Not mentioned.<br>• Type of breathing effort: The breathing effort was spontaneous and quiet.<br>• Hemidiaphragm assessed: right side.<br>• Anatomical placement of the transducer/array: Zone of apposition event is not specifically detailed in this paper but referenced to previous studies.<br>• Image acquisition procedure: B-Mode images were acquired using the same ultrasound machines (Philips Medical Capital, Netherlands), with a specific transducer model set to preset default values (frame rate 34 Hz, depth 5 cm, DB gain 50, Focus: 2.8 cm, time-gain compensation: 50%).<br>• Image analysis procedure: Offline analysis using ImageJ software for grayscale histogram analysis; two blinded observers analysed single frames to

	quantify echodensity. The analysis involved selecting the largest free-form region (trace method) devoid of artifacts between the pleural and peritoneal membranes.<br>• Number of images analysed to retrieve results: Not mentioned.<br>• New Ultrasound markers: ED50 (50th percentile of grayscale values), ED85 (85th percentile of grayscale values), EDmean (mean grayscale value)
**Comparator(s)**	1) Diaphragm echodensity metrics (ED50, ED85, EDmean) between patients who developed PPCs and those who did not.<br>2) The predictive ability of diaphragm echodensity compared to other scoring systems for predicting PPCs, such as the ARISCAT score
**Reliability/Feasibility**	The average difference in ED50 between analyzers was 1.63 (limits of agreement −17 to 20, p = 0.158), while for ED85 it was 3.24 (limits of agreement −32 to 38), and for EDmean it was 1.24 (limits of agreement −19 to 22). The average difference in ED50 between images from the same analyzer was 0.30 (limits of agreement −8 to 8, p = 0.700), with ED85 showing −0.14 (limits of agreement −13 to 13) and EDmean showing −0.07 (limits of agreement −7 to 7). Within a single respiratory cycle, the ED50 measured by the same analyzer differed between end-expiration and end-inspiration by 0.50 (limits of agreement −7 to 8, p = 0.476), while ED85 was 2.14 (limits of agreement −12 to 17) and EDmean was 1.94 (limits of agreement −4 to 8).
**Physiological and/or technical outcomes**	N/a
**Clinical outcomes**	Patients who developed PPCs exhibited significantly higher echodensity values: ED50 was 35.00 (22.00–44.00) in the PPC group versus 26.00 (21.00–35.00) in the non-PPC group (p < 0.001), ED85 was 64.00 (49.00–76.00) in the PPC group versus 55.00 (44.00–63.00) in the non-PPC group (p < 0.001), and EDmean was 39.32 (29.88–48.37) in the PPC group versus 33.98 (26.14–41.37) in the non-PPC group (p < 0.001).<br>Receiver operating characteristic (ROC) curve analysis indicated that the area under the curve for ED50 predicting PPCs was 0.611 (0.516-0.699), for EDmean it was 0.603 (0.508-0.692), and for ED85 it was 0.612 (0.517-0.700). The optimal ED50 cutoff value for predicting PPCs was determined to be 36, categorizing patients into high-risk (ED50 > 36, n = 35) and low-risk (ED50 ≤ 36, n = 82) groups.<br>Univariate logistic regression revealed several factors linked to postoperative pulmonary complications (PPCs), including diaphragm echodensity: EDmean (OR, 1.032 [95% CI, 1.028–1.036]), ED50 (OR, 1.037 [95% CI, 1.033–1.041]), and ED85 (OR, 1.018 [95% CI, 1.016–1.020]). Furthermore, multivariable logistic regression analysis confirmed that higher diaphragm echodensity was independently associated with PPCs, with the following odds ratios: EDmean (OR,

	1.026 [95% CI, 1.022–1.029], p < 0.001), ED50 (OR, 1.032 [95% CI, 1.027–1.036], p < 0.001), and ED85 (OR, 1.014 [95% CI, 1.012–1.017], p < 0.001).<br>In the high-risk group (ED50 > 36), 24 (68.57%) patients developed postoperative pulmonary complications (PPCs), which was significantly different from the low-risk group (p = 0.003). After multivariate-adjusted analyses, the significant difference in PPC incidence between the high-risk and low-risk groups persisted (p = 0.003). The high-risk group had higher incidences of PPCs in both unadjusted (p = 0.003) and multivariate-adjusted analyses (p = 0.003).<br>There were no significant differences observed in ventilator-free days at 28 (VFD-28), with values of 24.61 ± 3.99 in the low-risk group compared to 25.65 ± 3.71 in the high-risk group (p = 0.173). Similarly, the length of ICU stay was 8.03 ± 7.29 days in the low-risk group versus 5.79 ± 5.63 days in the high-risk group (p = 0.107), and the length of hospital stay was 20.25 ± 21.33 days in the low-risk group compared to 16.09 ± 12.10 days in the high-risk group (p = 0.279). Additionally, there were no significant differences in the mortality rate (p = 0.294).

**Author**	**Goutman SA, 2017**
**Country**	United States
**Study design**	Observational study, single-centre study
**Population**	- Healthy adults<br>- Number of participants: 6<br>- Age: Mean age 57.7 years (standard deviation 9.1 years)<br>- Male/Female: 3 women, 3 men<br>- No history of pulmonary or neuromuscular disease
**New/advanced ultrasound approach and technical features**	**The innovative ultrasound technique is speckle tracking which measures diaphragm movement in two dimensions (cephalocaudad and mediolateral).**<br><br>• Timing of the assessment: Not specified.<br>• Position of participant: Semirecumbent<br>• Type of breathing effort: Normal and deep breathing<br>• Hemidiaphragm assessed: Both right and left hemidiaphragms<br>• Anatomical placement of the transducer/array: For the speckle tracking loops, the probe was positioned coronally along the mid-axillary line, capturing images of each hemidiaphragm between the ribs. Ultrasound images were taken of the right and left hemidiaphragms during both normal and deep breathing.

	• Image acquisition procedure: Ultrasound images were acquired using an Epic ultrasound machine (Philips Ultrasound, Bothell, Washington) with a standard abdominal imaging curved array transducer (C5-1).<br>• Image analysis procedure: Image analysis was performed offline using EchoInsight software (Epsilon Imaging, Ann Arbor, Michigan). Speckle Tracking was conducted using EchoInsight software (Epsilon Imaging, Ann Arbor, Michigan), which tracks 2D speckle motion in cardiac echograms. After importing DICOM files, a 0.5 cm region-of-interest (ROI) curve was placed over the diaphragm using the define wall tool. EchoInsight generated six measurement segments from the ROI curve. Each ROI curve measured cephalocaudad displacement, calculated as the square root of the sum of the squared X and Y displacements, averaged across the six segments for each loop.<br>• Number of images analysed to retrieve results: At least three B-mode and M-mode samples for each hemidiaphragm.<br>• New Ultrasound markers: Speckle tracking using EchoInsight software to measure diaphragm movement in two dimensions (cephalocaudad and mediolateral) with units typically expressed in centimeters (cm).
**Comparator(s)**	M-mode ultrasonography for measuring diaphragm excursion.
**Reliability/Feasibility**	Speckle tracking was able to visualize the left hemidiaphragm in all 6 subjects during normal breathing and 5 out of 6 subjects during deep breathing, whereas M-mode could only visualize the left hemidiaphragm in 2 subjects during normal breathing and none during deep breathing.
**Physiological and/or technical outcomes**	The average difference between right speckle tracking and M-mode for normal and deep inspiration was 0.30 cm and -0.65 cm, respectively, and on the left diaphragm, it was 1.00 cm for normal inspiration.
**Clinical outcomes**	N/a

**Author**	**Hatam N, 2014**
**Country**	Germany
**Study design**	Observational study, single-centre study
**Population**	- Type of Population: Healthy subjects.<br>- Number of Participants: 13.<br>- Age: 27 years ± 7 years.<br>- Male/Female: All participants were male<br>Detailed Information about Patients' Disease: Since the participants were healthy individuals, there were no specific disease characteristics.

**New/advanced ultrasound technique**	**Speckle Tracking. This method allows for the quantification of diaphragmatic tissue deformation by tracking characteristic grayscale patterns.**<br><br>• Timing of the Assessment: Not applicable.<br>• Position of participant: Upright seated position<br>• Type of Breathing Effort: Regular breathing, CPAP, and CPAP with varying levels of pressure support ventilation (PSV) - not supported spontaneous breathing (NoPAP), support with 5mbar CPAP (CPAP), 5mbar CPAP + 5 mbar PSV (PSV5), 5mbar CPAP + 10 mbar PSV (PSV10) and 5mbar CPAP + 15mbar PSV (PSV15)<br>• Hemidiaphragm Assessed: Right hemidiaphragm.<br>• Anatomical Placement of the Transducer/Array: The transducer was placed on the anterior axillary line at the right-lateral thoracic wall, longitudinal to the body axis.<br>• Image acquisition procedure: Used a 2-4 MHz phased array transducer (M5S) with a Vivid E 9 ultrasound machine (General Electric, Horton, Norway), sonography at 3 MHz, frame rate of 60 fps, penetration depth of 6 cm.<br>• Image Analysis Procedure: The image analysis was performed offline after the acquisition of cineloops of the entire breathing cycle. Respiratory curves were obtained from ECG electrodes that had been previously positioned and were recorded simultaneously with the ultrasound image. A cineloop capturing the entire breathing cycle was recorded for subsequent offline analysis.<br>• Image analysis procedure: Performed offline using EchoPac's Q-analysis tool, with ROI drawn along the diaphragm, calculating longitudinal and transverse deformation parameters.<br>• The recorded data was analysed offline using the 2D strain modality of EchoPac®'s Q-analysis tool with a ROI drawn along the diaphragm's length, above the peritoneal border, parallel to the muscle fibers at the start of each breathing cycle. The software calculated longitudinal and transverse deformation parameters, with longitudinal parameters reflecting diaphragmatic movement along the body axis and transverse parameters indicating muscle thickening. Measurements included inspiratory peak longitudinal strain (–%) and peak transverse strain (+%), which indicated maximum inspiratory muscle workload, as well as their respective strain rates. The cranio-caudal movement of the diaphragm was quantified as longitudinal displacement in millimeters using speckle tracking echocardiography (STE).

	• Number of Images Analysed to Retrieve Results: The study did not specify the exact number of images analysed, but it involved capturing a cineloop of the entire breathing cycle for detailed analysis.<br>◦ New Ultrasound Markers: inspiratory peak longitudinal strain (–%) and peak transverse strain (+%) to assess maximum inspiratory muscle workload; inspiratory peak longitudinal strain rate (–1/s) and peak transverse strain rate (+1/s) to evaluate maximum muscle deformation velocities; and expiratory peak longitudinal strain rate (+1/s) to indicate maximum expiratory muscle relaxation. Cranio-caudal movement of the diaphragm was derived as the longitudinal displacement in millimetres.
**Comparator(s)**	M-mode-based fractional thickening (FT)
**Reliability/Feasibility**	N/a
**Physiological and/or technical outcomes**	Transverse strain values showed a strong correlation with fractional thickening (FT), with both (transverse inspiratory deformation) TID peak strain and FT significantly increasing during CPAP and PSV, resulting in a Pearson's correlation coefficient of 0.753 ($p < 0.001$). The peak transverse strain rate also significantly increased in the treatment groups. However, longitudinal measurements did not show significant differences across the ventilator settings. Isolated correlation analyses for PEEP and PSV indicated weaker correlations for longitudinal displacement. The respiratory cycle length remained consistent at 3 to 4 seconds during PSV-ventilation, with no significant changes in the time to peak inspiratory longitudinal and transverse strain. Additionally, there were no significant changes in the peak expiratory longitudinal strain rate or the time to peak expiratory strain rate based on inspiratory duration.
**Clinical outcomes**	N/a

**Author**	**Li R, 2024**
**Country**	China
**Study design**	Prospective observational study, single-centre study
**Population**	- Type of Population: Critically ill patients requiring mechanical ventilation, divided into group weaning success (n = 51) and weaning failure (n = 35).<br>- Number of Participants: A total of 86 patients were enrolled in the study.<br>- Age: The average age of participants was 59.55 ± 13.61 years.<br>- Gender: Among the participants, there were 32 females (30.72%), and the remaining were males.<br>- The main causes for mechanical ventilation included: Acute cerebrovascular disease: 20 patients (23.3%), Acute heart failure: 23 patients (26.7%),

	Postoperative complications: 10 patients (11.6%), Severe pneumonia: 24 patients (27%)<br>- Duration of Mechanical Ventilation: The duration of mechanical ventilation was significantly different between the two groups, with the weaning success group having a median of 6 days (4, 8) and the weaning failure group having a median of 15 days (12, 17).<br>- Severity Assessment: The severity of illness was assessed using the APACHE II score, with the mean scores being 21.63 ± 10 for the weaning success group and 24.54 ± 10 for the weaning failure group. For the SOFA score - weaning Success Group: mean was 7.73 ± 4, while weaning Failure Group: mean was 7.91 ± 3.
**New/advanced ultrasound technique**	**The innovative ultrasound technique utilised in this study is speckle tracking ultrasound.**<br><br>• Timing of the Assessment: The assessment was performed after the spontaneous breathing trial (SBT).<br>• Position of Participant: Participants were positioned in a supine posture with the head of the bed elevated at a 30° angle.<br>• Type of Breathing Effort: The assessment was conducted during quiet breathing.<br>• Hemidiaphragm Assessed: Right hemidiaphragms.<br>• Anatomical Placement of the Transducer/Array: The ultrasound probe was placed near the junction of the right midclavicular line and the costal margin, focusing on the liver to visualize the diaphragm.<br>• Image acquisition procedure: M9 color Doppler ultrasound system (Mindray Biomedical Co., Ltd., Shenzhen, China) with SP5-1s array probe; gain optimised for visualization, depth set to 10-15 cm, frame rate at 50 frames per second. Clips covering three respiratory cycles, at least 12 s, were recorded.<br>• Image Analysis Procedure: The built-in speckle tracking software of the ultrasound machine was used to identify a stable inspiratory phase and manually outline three regions of interest (ROIs): Costal Diaphragm, diaphragmatic dome, and Crural Diaphragm. At least seven points were marked in each ROI, and tracking points were automatically generated. Adjustments were made to align the ROIs with the pleura, diaphragm, and peritoneum. The longitudinal strain analysis model along the parasternal long axis was selected, with tracking initiated once at least 80% of the diaphragmatic speckles were trackable. The study permitted re-drawing or re-tracking, discarding any images with incomplete tracking.<br>• Number of Images Analysed to Retrieve Results: Not specified. The authors noted that at least three respiratory cycles were recorded.

	• New Ultrasound markers: Whole Strain (%), Costal Diaphragm Strain (DIcos) (%), Diaphragmatic Dome Strain (DIdome) (%), Crural Diaphragm Strain (DIcru) (%).
**Comparator(s)**	Diaphragm excursion (DE) measured by M-mode ultrasound; Diaphragmatic thickening fraction (DTF) measured by M-mode ultrasound.
**Reliability/Feasibility**	N/a
**Physiological and/or technical outcomes**	N/a
**Clinical outcomes**	There were significant differences between the successful weaning group and the weaning failure group. The weaning failure group exhibited notable decreases in Whole Strain ($p < 0.001$), DIcos Strain ($p < 0.001$), DIcru Strain ($p = 0.001$), DE ($p < 0.001$), and DTF ($p < 0.001$). Although DIdome Strain showed a decreasing trend, it was not statistically significant ($p = 0.334$), and no differences were found in DTei and DTee between the groups.<br>ROC analysis indicated that a DIcos strain value greater than −9.836 had a diagnostic value with an area under the curve (AUC) of 0.760, achieving a sensitivity of 80% and specificity of 72.5% for predicting successful weaning. A DE value exceeding 1.015 cm had an AUC of 0.785, demonstrating high specificity (90.2%) but lower sensitivity (60%). When DE was combined with Whole Strain, the AUC increased to 0.856, with balanced sensitivity (80%) and specificity (80.4%).<br>Univariate regression analyses showed strong associations between Whole Strain, DIcos Strain, DIcru Strain, DE, and DTF with weaning outcomes. Multivariate logistic regression identified Whole Strain (odds ratio [OR]: 1.962, 95% confidence interval [CI]: 1.042–3.655, $p = 0.037$) and DE (OR: 0.107, 95% CI: 0.024–0.486, $p = 0.004$) as independent predictors of weaning outcomes in ICU patients.

**Author**	**Nørskov J, 2024**
**Country**	Denmark
**Study design**	Prospective observation study, single centre
**Population**	- Type of Population: Patients undergoing esophageal resection or pulmonary lobectomy.<br>- Number of Participants: A total of 40 patients were enrolled in the study.<br>- Age: The median age of participants was 67 years (interquartile range: 61 to 74 years).

	- Male/Female: Out of the 40 participants, 29 were male (73%) and 11 were female (27%).<br>- Detailed Information about Patients' Disease:<br>o Comorbidities: Cardiovascular disease: 8 patients (20%), Diabetes mellitus: 3 patients (8%), COPD: 5 patients (13%), Asthma: 3 patients (8%), Hypertension: 19 patients (48%) and Chronic kidney disease: 3 patients (8%).<br>o Charlson Comorbidity Score:<br>o 0–3: 1 patient (3%)<br>o 4–6: 19 patients (48%)<br>o 7–9: 13 patients (33%)<br>o 10–12: 7 patients (18%)<br>o ASA Score (American Society of Anesthesiologists):<br>o I: 0 patients (0%)<br>o II: 6 patients (15%)<br>o III: 34 patients (85%)<br>o IV: 0 patients (0%)
**New/advanced ultrasound approach and technical features**	**The innovative ultrasound technique is the "Area method," which assesses diaphragm movement in two dimensions (cranio-caudal and posterior-anterior).**<br><br>• Timing of the assessment: Assessments were performed at baseline (the day prior to surgery), 3 days after surgery, and 10–14 days after surgery.<br>• Position of patient: Patients were positioned in a semi-recumbent position during the ultrasound examinations.<br>• Type of breathing effort: The breathing effort was standardised, with patients instructed to perform deep breathing through a turbohaler training whistle attached to a plastic bag to maintain a fixed inspiratory pressure threshold.<br>• Hemidiaphragm assessed: Both sides.<br>• Anatomical placement of the transducer/array: transducer was placed in the midaxillary line to identify the right or left hemidiaphragm. The transducer was positioned vertically to allow for the observation of diaphragmatic movement as a cranio-caudal motion.<br>• Image acquisition procedure: The ultrasound images were acquired using a GE Vivid S70 ultrasound machine (GE Healthcare, Horten, Norway) with an M5Sc-D cardiac sector transducer for the Area Method. Cine loops were recorded for later analysis.<br>• Image analysis procedure: Image analysis involved tracing the hemidiaphragmatic domes from inspiration to expiration to calculate the

	change in area. This analysis was performed by an expert sonographer who was blinded to all other study data, although it was not specified whether the analysis was performed immediately or offline.<br>• Number of images analysed to retrieve results: The exact number of images analysed was not detailed in the provided text.<br>• New Ultrasound markers: Area Change - Measured in square centimetres (cm^2), calculated by tracking the curve of the hemidiaphragm.
**Comparator(s)**	No direct comparator. However, changes in the intrathoracic area (area change) were analysed for the two different group of participants.
**Reliability/Feasibility**	N/a
**Physiological and/or technical outcomes**	N/a
**Clinical outcomes**	Change in intrathoracic area: diaphragmatic excursion measured as change in intrathoracic area using the Area method changed significantly on the surgical side over time ($p < 0.001$). There was a significant reduction in intrathoracic area from the day before surgery to 3 days after surgery: mean Difference: -18.9 cm^2; 95% CI [−12.3 cm^2, −25.4 cm^2], $p < 0.001$. The reduction remained significant 10–14 days after surgery: mean difference: -13.3 cm^2; 95% CI [−5.2 cm^2, −21.3 cm^2], $p = 0.001$. No statistically significant change was observed on the non-surgical side between assessments ($p = 0.88$).<br>Sub-group analysis: No significant difference was found between the esophagectomy group and the lobectomy group for change in intrathoracic area over time on both the surgical side ($p = 0.77$) and non-surgical side ($p = 0.19$). There was also no significant change in intrathoracic area over time between patients undergoing VATS compared to thoracotomy on either the surgical side ($p = 0.33$) or the non-surgical side ($p = 0.79$).

**Author**	**Oppersma E, 2017**
**Country**	The Netherlands
**Study design**	Observational study (experimental) with a randomised stepwise threshold loading protocol, single-centre study
**Population**	- Healthy adult volunteers<br>- Number of participants: 15<br>- Age: 21.3 ± 2.3 years<br>- Male/female: 7 males / 8 females<br>- The study specifically excluded individuals with preexisting neuromuscular disorders or lung diseases, confirming that the participants were healthy

**New/advanced ultrasound approach and technical features**	**Speckle tracking ultrasound to assess diaphragm contractility by measuring strain and strain rate.**<br><br>• Timing of the assessment: Not applicable.<br>• Position of participant: Seated upright.<br>• Type of breathing effort: The breathing effort assessed was during inspiratory threshold loading protocol of 0–50% of the maximal inspiratory pressure.<br>• Hemidiaphragm assessed: Right.<br>• Anatomical placement of the transducer/array: The 9-MHz linear transducer was positioned in the right anterior axillary line longitudinal to the body axis, between the 9th-11th intercostal space, to image the hemidiaphragm above the liver. The hemidiaphragm appears as a central, less echogenic layer above the liver, situated between the echogenic peritoneal and pleural layers. The region of interest (ROI) was marked on the skin for standardization during offline data analysis.<br>• Image acquisition procedure: A Vivid E 9TM ultrasound machine (General Electric Healthcare) was used to acquire the diaphragm ultrasound images. Ultrasound recordings of the diaphragm were captured in the final minute of each inspiratory loading task, with a 10-second recording at the highest frame rate used for analysis.<br>• Image analysis procedure: Image analysis was performed offline using the two-dimensional (2D) strain modality of EchoPac's Q-analysis tool (software version BT 12; General Electric Healthcare). The cine loops were modified to correspond to a complete breathing cycle, using respiratory curves that were recorded simultaneously. The region of interest was placed from the peritoneal to the pleural line of the diaphragm, spanning approximately 5-7 points.<br>• Number of images analysed to retrieve results: The study does not provide the precise number of images analysed. However, the authors indicate that at least three measurements were conducted to assess the thickening fraction.<br>• New Ultrasound markers: The new ultrasound markers retrieved included strain (%) and strain rate (s^{-1}). Strain represents the relative change in length from an initial state (L_0) to a compressed state (L), defined as $\varepsilon = (L - L_0)/L_0$. Positive strain indicates stretching, while negative strain indicates shortening. An increase in strain corresponds to a more negative value, reflecting greater shortening. Strain rate measures the rate of deformation as ε" = dε/dt and is an instantaneous measurement that does not require a reference state.
**Comparator(s)**	Fractional thickening (FT) assessed by conventional ultrasound, Transdiaphragmatic pressure (Pdi), Diaphragm electric activity (EAdi).
**Reliability/Feasibility**	N/a

**Physiological and/or technical outcomes**	As the load increased, both strain and strain rate also rose significantly ($p < 0.001$). Strain increased from an average of -22 ± 7.6% at zero loading to -41.5 ± 10.1% at 50% loading. Similarly, the strain rate increased from approximately -0.48 ± 0.2 s at zero loading to -1.5 ± 0.7 s at 50% loading.<br>Speckle tracking ultrasound was found to be superior in estimating diaphragm contractility, with strain and strain rate showing high correlations with transdiaphragmatic pressure (strain $r^2 = 0.72$; strain rate $r^2 = 0.80$) and diaphragm electric activity (strain $r^2 = 0.60$; strain rate $r^2 = 0.66$).<br>No significant correlations were found between the thickening fraction (FT) and strain ($p = 0.654$) or strain rate ($p = 0.364$).
**Clinical outcomes**	N/a

**Author**	**Orde SR, 2016**
**Country**	United States
**Study design**	Observational study, single-centre study
**Population**	- Healthy adults<br>- Number of participants: 50<br>- Age: Median age 37 years (IQR: 30.2 to 39.8)<br>- Male/Female: 22 males, 28 females
**New/advanced ultrasound approach and technical features**	**Two-dimensional speckle-tracking imaging to assess diaphragm strain.**<br><br>• Timing of the assessment: not applicable<br>• Position of participant: Subjects were positioned semi-recumbent at a 45° head-up angle.<br>• Type of breathing effort: Participants were instructed to inhale up to 60% of their peak inspiratory capacity within one second.<br>• Hemidiaphragm assessed: Right.<br>• Anatomical placement of the transducer/array: The linear array transducer was placed in the right mid-axillary line above the costal margin, specifically at the 9th intercostal space.<br>• Image acquisition procedure: A commercially available ultrasound machine (Vivid E9) with a linear array transducer (2.5-8 MHz) was used. Imaging started in the right mid-axillary line above the costal margin, visualizing the diaphragm's zone of apposition near the 9th intercostal space. If lung expansion caused the "lung curtain" sign during inspiration, the transducer was shifted anteriorly along the rib space toward the anterior axillary line until the sign disappeared.

	• Image analysis procedure: Image analysis was performed offline using speckle tracking software, which followed unique groups of grey-scale pixels (kernels) to measure diaphragm strain. Three-second clips were recorded for offline speckle-tracking analysis using EchoPacs (GE Healthcare, Milwaukee, MI). Apical four-chamber longitudinal analysis focused on tracing the diaphragm muscle's inner hyperechogenic borders (pleura and peritoneal surfaces) during inspiration, analyzing only the central region of interest.<br>• Number of images analysed to retrieve results: Multiple images were recorded from the end of expiration through the end of inspiration, although the exact number analysed is not specified.<br>• Ultrasound markers retrieved: Strain (in %), calculated as Strain = (D2-D1)/D1 x 100, where D1 is expiratory time and D2 is inspiratory time.
**Comparator(s)**	Diaphragm thickening fraction (TF) and caudal displacement, which are conventional ultrasound methods used to assess diaphragm function.
**Reliability/Feasibility**	Inter-rater reliability for longitudinal strain: ICC 0.90 (95% CI: 0.61–0.98), coefficient of repeatability 24.3%<br>Intra-rater reliability for longitudinal strain: ICC 0.96 (95% CI: 0.88–0.99), coefficient of repeatability 19.4%
**Physiological and/or technical outcomes**	The study provides results on the comparison of two-dimensional speckle-tracking imaging against conventional ultrasound techniques. The mean right diaphragm longitudinal strain value was -40.3% (±9), which showed a moderate correlation with thickening fraction (TF) ($R^2 = 0.44$, $p < 0.0001$) but a weak correlation with caudal displacement ($R^2 = 0.14$, $p < 0.01$).
**Clinical outcomes**	N/a

**Author**	Petersen JK, 2024
**Country**	Denmark
**Study design**	Prospective observation study, single centre
**Population**	- Type of Population: Patients with pleural effusion needing thoracentesis due to suspected or known malignancy. For analysis, participants were then divided into two groups: Expandable lung and Non-Expandable Lung (NEL).<br>- Number of Participants: A total of 49 patients were included in the study after screening 132 patients.<br>- Age: The mean age of participants was 71 ± 9 years. For the NEL group specifically, the mean age was 76 years.<br>- Male/Female: 25 were male (51%) and 24 were female (49%). In the NEL group, 6 out of 14 (43%) were female.<br>Detailed Information about Patients' Disease:

	- Known Malignancy: A significant portion of the participants had a history of malignancy, with 31 out of 35 (89%) in the expandable lung group and 13 out of 14 (93%) in the NEL group.<br>- Pleural Fluid Volume Drained: The average volume of pleural fluid drained was 1178 mL (SD: 629 mL) for the entire cohort, with the NEL group averaging 888 mL (SD: 620 mL)<br>- Smoking Status: In the NEL group, smoking status was reported as follows: 3 current/ 10 former smokers (21% current, 71% former) and 1 never smoker (7%).<br>- Follow-Up Diagnoses: At follow-up, most patients (90%) were diagnosed with malignancy, including lung cancer (23), breast cancer (11), and mesothelioma (4).
**New/advanced ultrasound approach and technical features**	**The innovative ultrasound technique is the "Area method," which assesses diaphragm movement in two dimensions (cranio-caudal and posterior-anterior).**<br><br>• Timing of the Assessment: Pre-thoracentesis.<br>• Position of Patient: Seated upright, leaning forward, and resting upon a support stand.<br>• Type of Breathing Effort: Not explicitly mentioned; however, it is implied that the breathing effort was during quiet breathing, as patients were given at least 5 minutes of rest before the examination was conducted.<br>• Hemidiaphragm Assessed: Not explicitly mentioned; however, it is implied that the side was chosen based on the location of the thoracentesis: both sides<br>• Anatomical Placement of the Transducer/Array: The transducer was placed in a lateral, mid-axillary view targeting optimal visualization of the diaphragm.<br>• Image Acquisition Procedure: Used LOGIQ S8 (GE Healthcare) with a C1-6-D curved (2-5 MHz) transducer. The settings were optimised for diaphragm imaging. Four-second movie clips were recorded.<br>• Image Analysis Procedure: Offline. The area of diaphragm movement was calculated by tracing the region above the diaphragm during inspiration and expiration, using the area-function of the ultrasound machine.<br>• Number of images analysed to retrieve results: Not explicitly mentioned.<br>• New Ultrasound markers: Area Change: Measured in square centimetres (cm^2), calculated by tracking the curve of the hemidiaphragm.
**Comparator(s)**	No direct comparator. However, it was compared with other methods in terms of diagnostic propriety for cases of Non-Expandable Lung (NEL) following thoracentesis: M-Mode ultrasound (lung and diaphragm movement), B-Mode ultrasound (diaphragm movement), 2D Shear Wave Elastography (SWE) (parietal pleura, pleural effusion, visceral pleura).
**Reliability/Feasibility**	N/a

**Physiological and/or technical outcomes**	N/a
**Clinical outcomes**	Area method diagnostic propriety for cases of Non-Expandable Lung (NEL) following thoracentesis:<br>• Delta in cm^2, median (IQR): 11.50 (6.69–15.95)<br>• Area Under the Curve (AUC): 0.60, 95% CI: 0.40–0.79.<br>Comparison with Other Ultrasound Tests:<br>The Area method had an AUC of 0.60, which was lower than M-mode lung movement (AUC 0.81), M-mode diaphragm movement (AUC 0.77) and B-mode diaphragm movement (AUC 0.65), being only higher than Shear-Wave Elastography for parietal pleura, pleural effusion, and visceral pleura, 0.57, 0.53, and 0.59 respectively.

**Author**	Sarwal A, 2015
**Country**	Australia and United States
**Study design**	Cross-sectional observational study, multicentric study (2 centres)
**Population**	- Type of population: Critically ill patients<br>- Number of participants: Not explicitly mentioned (20 diaphragm and 20 quadriceps images analysed)<br>- Age: Not mentioned<br>- Male/female: Not mentioned
**New/advanced ultrasound approach and technical features**	**Quantitative echogenicity analysis of the diaphragm.**<br><br>• Timing of the assessment: Not explicitly mentioned ("during different stages of their ICU stay").<br>• Position of participant: Supine position and the head of the bed elevated 30°.<br>• Type of breathing effort: The assessments were conducted during quiet breathing.<br>• Hemidiaphragm assessed: Right side.<br>• Anatomical placement of the transducer/array: Transducer placed in the right midaxillary line at the zone of apposition at end expiration.<br>• Image acquisition procedure: Images were acquired using an ultrasound machine with a linear 6–15-MHz transducer (M-Turbo; SonoSite, Inc, Bothell, WA), always perpendicular to the diaphragm muscle.<br>• Image analysis procedure: The image analysis for diaphragm echogenicity was conducted offline using ImageJ, a public domain image-processing program. Two measurement techniques were employed: the square technique, which utilised a predefined square area (e.g., 20 × 20 pixels) to

	assess echogenicity, and the trace technique, where the anatomical boundary of the diaphragm was traced to obtain measurements. To minimize bias, assessors were blinded to previous results, and images were randomly assigned for analysis. The analysis aimed to determine the mean echogenicity values of the diaphragm.<br>• Number of images analysed to retrieve results: echogenicity measurements were repeated twice, and the mean value taken to analysis.<br>• New Ultrasound markers: Mean echogenicity (ranging from 0 to 255) obtained from trace and square techniques (using grayscale analysis), which were calculated using the histogram feature of ImageJ.
**Comparator(s)**	No comparator
**Reliability/Feasibility**	- Echogenicity Measurements: No significant differences were found among the four raters in diaphragm muscle echogenicity as determined by either the trace (p = 0.86) or square method (p = 0.57).<br>- Interobserver Reliability: There was very good interobserver reliability for diaphragm echogenicity measurements, with ICC values ranging from 0.851 (95% CI 0.667 – 0.938) to 0.984 (95% CI 0.959 – 0.993). However, novice raters showed lower ICCs for diaphragm echogenicity compared to quadriceps.<br>- Comparison of Methods: A significant difference in mean echogenicity values was observed between the square and trace methods for both muscles. For the diaphragm, the mean difference between the square and trace methods was 1.14 (95% CI: 0.23–2.05; p = 0.02).<br>- Bland-Altman Analysis: This analysis indicated wider limits of agreement in novice raters for all quantitative measurements (thickness and echogenicity) of the diaphragm compared to experienced assessors.
**Physiological and/or technical outcomes**	N/a
**Clinical outcomes**	N/a

**Author**	**Şendur HN, 2022**
**Country**	Türkiye
**Study design**	Prospective observational study, single-centre study
**Population**	Healthy volunteers and COPD patients<br>- Number of participants: 40 healthy participants and 8 COPD patients<br>- Age: Healthy participants mean age 40.9 ± 15.1 years (range 19-69), COPD patients mean age 60.3 ± 11.1 years (range 48-77)<br>- Male/female: Healthy participants 20 males and 20 females, COPD patients 6 males and 2 females

	- COPD patients were clinically stable with a mean disease duration of 5.75 years.
**New/advanced ultrasound approach and technical features**	**Ultrasound shear wave elastography (SWE) to assess diaphragm stiffness.**<br><br>• Timing of the assessment: During outpatient consultation at the sonography imaging unit.<br>• Position of participant: Participants were positioned in a supine position with their right hand placed above their head.<br>• Type of breathing effort: The breathing effort was normal, without any specific instructions for deep or forced breathing.<br>• Hemidiaphragm assessed: Right side.<br>• Anatomical placement of the transducer/array: Between the anterior and middle axillary lines of ninth or 10th intercostal spaces (zone of apposition).<br>• Image acquisition procedure: US device (RS85; Samsung Medison Co, Ltd) with a 9-MHz linear transducer. Participants were in supine position with the right hand above the head. The transducer was held perpendicular to the chest wall. After a minimum of five respiratory cycles, SWE evaluation was conducted. The SWE images were captured during the peak inspiration and end-expiration phases of the respiratory cycle.<br>• Image analysis procedure: Image analysis was performed offline after the acquisition, where the stiffness values were measured in kilopascals (kPa) using four regions of interest (ROIs) at each respiratory phase.<br>• Number of images analysed to retrieve results: A total of three images were analysed for each respiratory phase to obtain the stiffness measurements.<br>• New ultrasound markers: Diaphragm stiffness values measured in kilopascals (kPa) during both inspiratory and expiratory phases.
**Comparator(s)**	No comparator
**Reliability/Feasibility**	- Diaphragm stiffness measurements had good inter-observer reliability, with ICC values of 0.667 (95% CI, 0.452–0.809) at peak inspiration and 0.736 (95% CI, 0.553–0.851) at end expiration.<br>- The mean difference in stiffness measurements between radiologists was smaller at end expiration (0.97 ± 7.33 kPa) compared to peak inspiration (2.23 ± 12.57 kPa).
**Physiological and/or technical outcomes**	The stiffness of the diaphragm significantly increased with inspiration ($P < 0.001$ for the measurements of both radiologists). The mean ± SD stiffness of the diaphragm at the peak inspiration and end expiration phases was 51.84 ± 16.83 kPa and 38.49 ± 9.42 kPa, respectively, for the first radiologist, and 49.61 ± 13.83 kPa and 37.52 ± 10.71 kPa, respectively, for the second radiologist. The difference between mean stiffness values of the diaphragm at the peak inspiration

	and end expiration phases was 13.35 kPa for the first radiologist and 12.09 kPa for the second radiologist.<br>In COPD patients, the mean ± SD stiffness of the diaphragm at the peak inspiration and end expiration phases was 35.83 ± 8.64 kPa and 33.25 ± 10.76 kPa, respectively. Although the thickness of the diaphragm significantly increased at peak inspiration compared to end expiration (2.69 ± 0.46 mm vs. 2.25 ± 0.35 mm, respectively, $p = 0.012$), there was no significant difference in stiffness measurements between respiratory phases ($p > 0.05$).
**Clinical outcomes**	N/a

**Author**	**Skaarup SH, 2018**
**Country**	Denmark
**Study design**	Cohort study, single-centre study
**Population**	- Healthy adult volunteers (no specific diseases)<br>- Number of participants: 19<br>- Age: Mean age 23 years (SD 1.6)<br>- Male/Female: 63.4% male, 36.6% female
**New/advanced ultrasound approach and technical features**	**The innovative ultrasound technique is the "Area method," which assesses diaphragm movement in two dimensions (cranio-caudal and posterior-anterior).**<br><br>• Timing of the assessment: Not applicable<br>• Position of participant: Erect position.<br>• Type of breathing effort: Participants performed various breathing maneuvers, including maximal inspiration and shallow breathing, while avoiding forced exhalation.<br>• Hemidiaphragm assessed: Both the right and left hemidiaphragms were assessed.<br>• Anatomical placement of the transducer/array: The transducer was placed in a lateral mid-axillary view to visualize both hemidiaphragms effectively.<br>• Image acquisition procedure: A curvilinear 3–5 MHz probe was used with a General Electric Vivid S8 ultrasound machine, employing a standard abdominal preset for the ultrasound measurements (B-Mode). On the right side, the liver served as a sonographic landmark to locate the right hemidiaphragm, while on the left side, the spleen was used as the landmark.<br>• Image analysis procedure: The analysis of diaphragm movement was performed offline, with film clips saved for later evaluation. The ultrasound operator was blinded to the spirometry measurement results during analysis.

	An image frame showing maximal diaphragm contraction at end-inspiration was identified by scrolling through the film. Using the ultrasound machine's area-calculation function, the entire visible portion of the diaphragm was traced. If part of the diaphragm was obscured, tracing continued from the visible sections. The ultrasound transducer remained fixed during the respiratory maneuver, allowing the image borders to serve as area limits, which did not change. The only variation in area resulted from the diaphragm's movement during respiration. This method calculated the intra-thoracic area at maximal diaphragm contraction. The frame with minimal diaphragm contraction at pre-inspiration was then located, and the intra-thoracic area was calculated similarly.<br>• Number of images analysed to retrieve results: The exact number of images analysed per participant was not specified, but multiple film clips were recorded for comprehensive assessment. Overall number of images analysed: 72 observations for the Area method.<br>• New Ultrasound markers: The Area method, which quantifies changes in intra-thoracic area during respiration in cm². It assesses diaphragm movement in two dimensions: cranio-caudal and posterior-anterior. Calculation: *Δ intrathoracic area during respiration = intra-thoracic area in maximal diaphragm contraction − intra-thoracic area in minimal diaphragm contraction.*
**Comparator(s)**	The comparators in this study are the M-mode and B-mode ultrasound methods for assessing diaphragm movement.
**Reliability/Feasibility**	Area method and existing M-mode and B-mode methods for assessing diaphragm movement. The inter-rater agreement was high for the Area method (intra-class correlation = 0.9, $p < 0.001$) and the M-mode method (intra-class correlation = 0.9, $p < 0.001$), but lower for the B-mode method (intra-class correlation = 0.8, $p < 0.001$).<br>Regarding feasibility, five novice operators conducted ultrasound examinations of the diaphragm on three healthy volunteers. They were unable to obtain an ultrasound image of the left hemidiaphragm in 80% (95% CI 0.44–0.97) of the examinations performed at the left mid-clavicular line (required for M-Mode). In contrast, only 10% (95% CI 0–0.45) of the examinations at the right mid-clavicular line (required for M-Mode) were unsuccessful. However, in all examinations (95% CI 0.69–1), the diaphragm was visualised on both the right and left sides from a mid-axillary view (for the area method).
**Physiological and/or technical outcomes**	The Area method showed higher correlation with expired lung volume (0.88, 95% CI 0.81–0.95, $p < 0.001$) compared to B-mode (0.71, 95% CI 0.59–0.83, $p < 0.001$) and similar to M-mode (0.84, 95% CI 0.75–0.92, $p < 0.001$).<br>When dividing in low and high volume, in case of low-volume, the correlation coefficient for M-mode was 0.85 (95% CI 0.74–0.96, $p < 0.01$), while the area

	method showed a coefficient of 0.84 (95% CI 0.71–0.96, p < 0.01). The B-mode had a lower correlation of 0.61 (95% CI 0.35–0.86, p < 0.01). In the high-volume category, M-mode had a correlation coefficient of 0.43 (95% CI 0.17–0.68, p < 0.01), the area method was 0.29 (95% CI 0.07–0.64, p = 0.11), and B-mode had a coefficient of 0.14 (95% CI -0.19–0.48, p = 0.4).
**Clinical outcomes**	N/a

**Author**	**Skaarup SH, 2020**
**Country**	Italy
**Study design**	Prospective observational study, single-centre study
**Population**	- Type of population: Patients with pleural effusion (unilateral)<br>- Number of participants: 32<br>- Age: Mean 72 years (SD 16.1)<br>- Male/Female: 17 males, 15 females<br>- BMI: mean 24.2 (SD, 7.2) kg/m^2<br>- Detailed information about patients' disease: In total, 35.7% of the patients had clinically important comorbidity. An overall 9.5% had >1 disease. Comorbidities include arterial hypertension, COPD, cancer, ischemic coronary disease, type 2 diabetes, chronic renal failure, among others. Causes of pleural effusion include parapneumonic pleural effusion, malignant pleural effusion, and others.
**New/advanced ultrasound approach and technical features**	**The innovative ultrasound technique is the "Area method," which assesses diaphragm movement in two dimensions (cranio-caudal and posterior-anterior).**<br><br>• Timing of the assessment: The ultrasound evaluation was performed immediately before and right after thoracentesis.<br>• Position of participant: Participants were seated in an upright position during the assessment.<br>• Type of breathing effort: tidal breathing.<br>• Hemidiaphragm assessed: Both the affected (hemithorax side with pleural effusion) and the unaffected hemidiaphragms were evaluated.<br>• Anatomical placement of the transducer/array: The ultrasound transducer was placed at the midaxillary.<br>• Image acquisition procedure: An Esaote (Genoa, Italy) Mylab 30 CV ultrasound machine with a curvilinear probe and a standard abdominal pre-set was used. A film clip of the respiratory cycle was stored.<br>• Image analysis procedure: Image analysis was conducted using the area calculating function integrated into the ultrasound machine to evaluate

	diaphragm movement. Images were captured during both maximal inspiration and expiration, and the difference in area was calculated. This analysis was performed immediately after the images were taken.<br>• Number of images analysed to retrieve results: Specific details on the number of images analysed were not provided, but multiple images were captured as part of an ultrasound film clip during the respiratory cycle.<br>• New ultrasound markers: Area Change, measured in square centimetres (cm^2), calculated by tracking the curve of the hemidiaphragm.
**Comparator(s)**	Pre- and post-thoracentesis ultrasound evaluation of hemidiaphragmatic function.
**Reliability/Feasibility**	N/a
**Physiological and/or technical outcomes**	N/a
**Clinical outcomes**	Significant improvement in the movement of the affected hemidiaphragm, as measured by the ultrasound-based Area method. Affected hemidiaphragm's movement revealed a respiratory motion with a mean area of 7.4 cm^2 (95% CI, 5.14-9.56) prior to thoracentesis. Following the procedure, this improved significantly to 26.0 cm^2 (95% CI, 20.81-31.13, $p<0.0001$). In contrast, on the side without pleural effusion, the mean diaphragm movement was 26.3 cm^2 (95% CI, 21-31.63) before thoracentesis and increased to 27.9 cm^2 (95% CI, 20.35-35.47) afterward, with a p-value of 0.52.

**Author**	**Skaarup SH, 2024**
**Country**	Denmark
**Study design**	Prospective observational study, single-centre study
**Population**	- Type of population: COPD, interstitial lung disease, post-thoracic surgery (heart transplantation or LVAD implant), post-COVID-19 infection, healthy volunteers.<br>- Number of participants: 42<br>- Age: Mean age is 59.7 ± 13.3 years<br>- Male/female: 63% (32 male) / 37% female (15 women)<br>- Lung function measurements indicated a mean FEV1 of 1.7 ± 1.1 L/min, corresponding to 50% ± 28% of the predicted value. The mean FVC was 3.1 ± 1.2 L, representing 79% ± 23% of the expected value, with an average FEV1/FVC ratio of 0.5 ± 0.2.
**New/advanced ultrasound approach and technical features**	**The innovative ultrasound technique is the "Area method," which assesses diaphragm movement in two dimensions (cranio-caudal and posterior-anterior).**

	• Timing of the assessment: The assessments were performed during a single visit. Participants were recruited from local Department of Respiratory Diseases and Allergy, local Department of Cardiology and from the local Clinic for long-Covid symptoms.<br>• Position of participant: Participants were positioned in an upright sitting position.<br>• Type of breathing effort: Slow inspiration to maximal inspiration (inspiratory capacity), quick forced sniff inspiration<br>• Hemidiaphragm assessed: Both the left and right hemidiaphragms were assessed.<br>• Anatomical placement of the transducer/array: authors used curvilinear transducer (3-5 Hz) placed in the left and right lateral positions using the spleen and liver as acoustic windows, respectively.<br>• Image acquisition procedure: Ultrasound recordings were made using ultrasonography equipment (Logiq S8 ultrasonography system from GE Healthcare), during two breathing manoeuvres - a slow inspiration to total inspiratory capacity, and a quick forced sniff inspiration.<br>• Image analysis procedure: Not defined in the study article. However, it is noted that ultrasound recordings were analysed using ultrasonography equipment.<br>• Number of images analysed to retrieve results: A total of ten recordings were made for each participant, capturing various breathing manoeuvres.<br>• New Ultrasound markers: Area Change: Measured in square centimetres (cm^2), calculated by tracking the curve of the hemidiaphragm.
**Comparator(s)**	- Fluoroscopy measures of diaphragm craniocaudal excursion.<br>- Dynamic lung function values of FVC and FEV1.
**Reliability/Feasibility**	N/a
**Physiological and/or technical outcomes**	• During five repeated inspiratory capacity (IC) maneuvers, the mean diaphragm excursion for the right hemidiaphragm measured by fluoroscopy was 4.0 cm (SD ± 2.2). In the midclavicular line, the M-mode excursion during IC was 4.3 cm (SD ± 1.7). During sniff inspiration, the M-mode excursion for the right hemidiaphragm was 3.3 cm (SD ± 1.1), with a contraction velocity of 7.9 cm/sec (SD ± 4.5). Additionally, the area change for the right hemidiaphragm was 36.4 cm^2 (SD ± 22) during IC inspiration and 26.1 cm^2 (SD ± 13.5) during sniff inspiration.<br>• The comparison of fluoroscopy and the Area method for assessing diaphragm function revealed the following results:<br>Left Hemidiaphragm:

	○ During maximal inspiratory capacity (IC), the linear regression coefficient for area change was 0.1 (94% CI 0.08–0.12, p < 0.001), with a Pearson correlation coefficient of 0.48.<br>○ During the sniff inspiration maneuver, the linear regression coefficient was 0.04 (95% CI 0.03–0.05, p < 0.001), and the Pearson correlation coefficient was 0.20.<br>○ FEV1: Coefficient = 0.04, 95% CI = [0.01, 0.07], p-value = 0.01, Pearson correlation = 0.5285.<br>○ FVC: Coefficient = 0.05, 95% CI = [0.03, 0.08], p-value = 0.001, Pearson correlation = 0.6913.<br>-Right Hemidiaphragm:<br>○ For IC, the linear regression coefficient was 0.1 (95% CI 0.07–0.11, p < 0.001), with a Pearson correlation coefficient of 0.34.<br>○ During the sniff inspiration maneuver, the linear regression coefficient was 0.05 (95% CI 0.03–0.07, p < 0.001), and the Pearson correlation coefficient was 0.02.<br>○ FEV1: Coefficient = 0.02, 95% CI = [0.01, 0.04], p-value = 0.01, Pearson correlation = 0.4598.<br>○ FVC: Coefficient = 0.02, 95% CI = [0.01, 0.04], p-value = 0.01, Pearson correlation = 0.4731.
**Clinical outcomes**	N/a

**Author**	**Soilemezi E, 2020**
**Country**	Greece
**Study design**	Prospective observational study, single-centre study
**Population**	- Healthy volunteers and critically ill ICU patients<br>- Number of participants: total of 136 (20 adult healthy subjects and 116 consecutive adult critically ill patients).<br>- Healthy subjects:<br>• Age: between 25 to 48 years<br>• Male/female: 10 men and 10 women<br>- Critically ill patients:<br>• Age: 66 ± 13 years<br>• Male/female: 87 male (75.2%)<br>• Mechanically ventilated for more than 48 hours.<br>• Admitted for various reasons such as respiratory failure: 22 patients (18.9%), multiple trauma: 16 patients (13.8%), sepsis: 16 patients (13.8%), coma: 6

	patients (5.2%), neurosurgery: 10 patients (8.6%), cardiac surgery: 8 patients (6.9%), other major surgery: 10 patients (8.6%), other: 28 patients (24.1%)
**New/advanced ultrasound approach and technical features**	**Tissue Doppler Imaging (TDI) applied to the diaphragm.**<br><br>• Timing of the assessment: At the end of a 30-minute T-piece weaning trial or immediately before reconnection to the ventilator.<br>• Position of participant: Laying on a bed with the back elevated at 30 degrees.<br>• Type of breathing effort: Spontaneous breathing.<br>• Hemidiaphragm assessed: Right.<br>• Anatomical placement of the transducer/array: Subcostal position between the midclavicular and anterior axillary lines.<br>• Image acquisition procedure: Phased array 2-4 MHz probe (Philips Sparq ultrasound machine).<br>• Image analysis procedure: Image analysis was performed immediately after acquisition, focusing on the TDI-derived parameters such as contraction and relaxation velocities. The analysis involved assessing the waveform pattern of diaphragmatic motion.<br>• Number of images analysed to retrieve results: Eight to ten breaths per subject.<br>• Ultrasound markers: Peak contraction velocity (PCV, cm/s), Peak relaxation velocity (PRV, cm/s), Velocity-time integral (VTI, cm), TDI-derived maximal relaxation rate (TDI-MRR, cm/s^2)
**Comparator(s)**	Transdiaphragmatic pressure (Pdi)
**Reliability/Feasibility**	Excellent intra- and interobserver reproducibility was found for all variables (PCV, PRV, VTI, TDI-MRR), with an ICC above 0.89 for all measurements.<br>Coefficient of variance values were all within 10% (range, 2.49–8.81).
**Physiological and/or technical outcomes**	Significant correlation between peak Pdi and PCV ($R^2 = 0.727$; $p < 0.001$), PTPdi and PCV ($R^2 = 0.650$; $p = 0.007$), and Pdi-MRR and TDI-MRR ($R^2 = 0.634$; $p < 0.001$). A weaker correlation was recorded between VTI and PTPdi ($R^2 = 0.285$).
**Clinical outcomes**	Healthy volunteers and weaning success patients exhibited lower values for all TDI parameters compared with weaning failure patients, except for velocity–time integral (VTI), as follows: peak contraction velocity (PCV), 1.35 ± 0.34 versus 1.50 ± 0.59 versus 2.66 ± 2.14 cm/s ($p < 0.001$); peak relaxation velocity (PRV), 1.19 ± 0.39 versus 1.53 ± 0.73 versus 3.36 ± 2.40 cm/s ($p < 0.001$); and TDI-maximal relaxation rate (MRR), 3.64 ±2.02 versus 10.25 ± 5.88 versus 29.47 ± 23.95 cm/s2 ($p < 0.001$), respectively.

**Author**	**Umbrello M, 2021**

**Country**	Italy
**Study design**	Prospective observational study, single-centre study
**Population**	- Type of Population: Critically ill patients diagnosed with COVID-19, specifically those admitted to the Intensive Care Unit (ICU) with acute respiratory distress syndrome (ARDS).<br>- Number of Participants: A total of 36 consecutive patients were enrolled in the study. 8 patients died before day 7 ; ultrasound images at admission and after 7 days were available for the remaining 28 patients.<br>- Age: 65 ± 10 years.<br>- Male/Female Ratio: Approximately 80% of the participants were male.<br>- Severity Scores: The Simplified Acute Physiology Score (SAPS II) had a median score of 31 (interquartile range [IQR]: 25-37) for survivors and 37 (IQR: 30-41) for non-survivors. The Sequential Organ Failure Assessment (SOFA) score was 3 (IQR: 3-4) for survivors and 4 (IQR: 3-6) for non-survivors.<br>- Comorbidities: Common comorbidities included hypertension (42.8% in survivors, 57.1% in non-survivors) and obesity (50% in survivors, 35.7% in non-survivors).<br>- Duration of Symptoms Before Hospitalization: The median duration of symptoms before hospitalization was 5 days (IQR: 3-7).<br>- Duration of Symptoms Before Intubation: The median duration of symptoms before intubation was 9 days (IQR: 7-14).<br>- Mechanical Ventilation: All patients were mechanically ventilated upon ICU admission and received muscle relaxants during the first week of their stay.<br>- Overall ICU mortality was 58.3%.<br>- Duration of mechanical ventilation (days): Survivors: 14 [9-21], Non-survivors: 16 [7-24] (p=0.8871)<br>- ICU length of stay (days): Survivors: 19 [14-26], Non-survivors: 16 [7-24] (p=0.2415)<br>- Ventilator-free days: Survivors: 2 [1-4], Non-survivors: 0 [0-0] (p<0.0001)<br>- Hospital length of stay (days): Survivors: 47 [30-57], Non-survivors: 21 [15-28] (p=0.0052)
**New/advanced ultrasound technique**	**The innovative ultrasound technique is the assessment of diaphragm echogenicity.**<br><br>• Timing of the Assessment: The assessments were performed within 24 hours of ICU admission and again on day 7 of the ICU stay.<br>• Position of participant: Patients were positioned at 30 degrees for diaphragm measurements.<br>• Type of Breathing Effort: Passive (mechanically ventilated, deeply sedated at ICU admission).

	• Hemidiaphragm Assessed: Right hemidiaphragm.<br>• Anatomical Placement of the Transducer/Array: The transducer was placed above the right 10th rib in the midaxillary line, at the zone of apposition.<br>• Image Acquisition Procedure: B-mode images were obtained using a 6 to 14 MHz linear array transducer on an Esaote MyLab X8 ultrasound device. The operator applied minimal pressure to optimize imaging conditions while ensuring the probe was adequately covered with gel. Skin landmarks were temporarily marked to improve reproducibility. The ultrasound device settings, including depth and gain, were maintained consistently by using the same image presets for all patients.<br>• Image Analysis Procedure: Image analysis was performed offline using ImageJ software. A gray-scale frequency histogram was generated for the selected area devoid of artifacts (apparently trace method - not explicitly mentioned) allowing for quantification of echo density. The analysis was conducted on saved JPEG images.<br>• Number of Images Analysed to Retrieve Results: Three consecutive measurements (within 10% of variability) were taken for each assessment and the average of these measurements was reported.<br>• New Ultrasound Markers: Mean Echogenicity/Echo Density: Quantified using gray-scale values ranging from 0 (black) to 255 (white).
**Comparator(s)**	Changes in muscle size (thickness) and quality (echogenicity) in relation to the amount of nutritional support delivered and the cumulative fluid balance. Participants were also divided in two groups: survivors and non-survivors.
**Reliability/Feasibility**	The ICC for intrarater reproducibility of diaphragm echogenicity was 0.998 (95% CI, 0.996-0.999); the values for interrater reproducibility of diaphragm echogenicity were 0.998 (95% CI, 0.997-0.999).
**Physiological and/or technical outcomes**	The change in diaphragm echo density was positively related to the cumulative fluid balance ($R^2 = 0.417$, $p < 0.001$) and was not related to the cumulative protein deficit ($R^2 = 0.083$, $p = 0.137$).
**Clinical outcomes**	Diaphragm echo density significantly increased during the first 7 days of ICU stay ($p < 0.0001$).<br>Non-survivors had a significantly higher diaphragm echo density compared to survivors; The increase in diaphragm echo density from baseline to day 7 was significantly greater in non-survivors; at admission, the percentage change for survivors were 0.1 [IQR, -12.6 to 17.5], while for non-survivors, the percentage change was 14.6 [IQR, 9.5 to 24.3], p = 0.0169.

**Author**	**van Doorn JLM, 2022**

**Country**	The Netherlands
**Study design**	Retrospective observational study, single-centre study
**Population**	- Type of population: Healthy subjects<br>- Number of participants: 83<br>- Age: mean 39.0 years (22.9)<br>- Male/female: 40/43<br>- The authors decided to target the enrolment of 10 healthy participants, comprising 5 males and 5 females, for each decade of age to ensure a balanced distribution of age and sex.
**New/advanced ultrasound approach and technical features**	**The innovative ultrasound technique is the assessment of diaphragm echogenicity.**<br><br>• Timing of the assessment: Not applicable (study on healthy subjects)<br>• Position of participant: supine position<br>• Type of breathing effort: The assessment was conducted during quiet breathing.<br>• Hemidiaphragm assessed: Right side.<br>• Anatomical placement of the transducer/array: zone of apposition.<br>• Image acquisition procedure: An Esaote MyLab Twice ultrasound machine equipped with a 3–13 MHz LA533 linear transducer was used for image acquisition. The same preset was used without changing any settings, as this was important for measuring echogenicity. Imaging settings such as depth and zoom also impacted echogenicity measurements.<br>• Image analysis procedure: Image analysis was performed offline using custom-developed software in Matlab (R2018a, Mathworks, Natick, MA, USA). The mean pixel gray-value of a manually selected region-of-interest in the diaphragm muscular tissue was calculated, averaged over three measurements.<br>• Number of images analysed to retrieve results: A total of three images were analysed for each participant to ensure accuracy in the echogenicity measurements.<br>• New Ultrasound markers: The primary new ultrasound marker retrieved was diaphragm echogenicity, measured in mean pixel gray-values (ranging from 0 to 255). This marker provides insights into the muscle's structural integrity and function.
**Comparator(s)**	
**Reliability/Feasibility**	The intra-observer reliability for the three echogenicity (at end-expiration) measurements was found to be excellent, with intraclass correlation coefficient (ICC) of 0.93 (0.89–0.96).

**Physiological and/or technical outcomes**	N/a
**Clinical outcomes**	Regression analyses revealed that echogenicity increased with age, as indicated by the formula Echogenicity=71.443+0.390×cAge ($p < 0.001$)<br>*Note: cAge: centreed age, calculated by subtracting 39.0 from age in years.*

**Author**	**Watanabe S, 2024**
**Country**	Japan
**Study design**	Prospective cohort study, single-centre study
**Population**	- Type of population: Patients with amyotrophic lateral sclerosis (ALS) and healthy controls<br>- Number of participants: 40 (19 ALS patients, 21 healthy controls)<br>- Age: ALS patients mean age 62.2 ± 18.1 years; healthy controls mean age 54.6 ± 17.4 years<br>- Male/female: 14 males in both ALS patients and healthy controls<br>- Detailed information about patients' disease: ALS diagnosed by clinical symptoms and electromyography, following revised El-Escorial or Awaji criteria; average disease duration 25.2 ± 29.8 months.
**New/advanced ultrasound approach and technical features**	**The innovative ultrasound technique used is ultrasound speckle tracking.**<br><br>• Timing of the assessment: Not applicable.<br>• Position of participant: Participants were in a supine position during the assessment.<br>• Type of breathing effort: Quiet breathing.<br>• Hemidiaphragm assessed: Right side.<br>• Anatomical placement of the transducer/array: Anatomical placement of the transducer: Perpendicular to the chest wall at the zone of apposition on the 8th and 11th intercostal spaces along the anterior and midaxillary lines.<br>• Image acquisition procedure: The assessments were conducted using a LOGIQ e Premium ultrasound device (GE Healthcare) with an L4-12t-RS probe set at 10 MHz. The ultrasound device was configured for improved tracking accuracy with the following settings: (1) gain was set to 50–60 dB to enhance contrast; (2) probe frequency was 8 MHz; (3) frame rate was 50 frames per second; (4) the speckle reduction filter was disabled; and (5) the frame-averaging system, which averages images between frames and smooths the video, was also turned off. The probe was positioned to allow the diaphragm to move as much as possible in the direction of the tomographic plane. Video imaging recorded and stored in uncompressed AVI format.

	• Image analysis procedure: Performed offline using prototype software developed in Microsoft Visual C++:<br>○ Motion analysis was conducted using specialised prototype software. After loading the video, the operator defined a rectangular region of interest (ROI) at a specific location on the screen, encompassing the diaphragm layer (both surface and deep layers) as well as off-screen areas used as anchors. A square grid with a size of 5 pixels (kernels) was then displayed. The motion vector for each vertex of the grid was estimated frame-by-frame using a differential method for optical flow (Lucas-Kanade method). Three kernels along the same line were selected to track the central, deep, and surface diaphragmatic layers, measuring the movement for one breath at each location.<br>○ Two kernels were positioned on the central layer of the diaphragm, spaced 5 mm apart, around the identifiable midpoint between the two ribs. The strain, defined as the change in distance between the two kernels during expiration and inspiration, was calculated using the following formula: [(distance between the two kernels at inspiration - distance between the two kernels at the end of expiration) / distance between the two kernels at the end of expiration] × 100 (%).<br>• Number of images analysed to retrieve results: three times for each assessment and the mean value was taken to analysis.<br>• New Ultrasound markers: Diaphragm moving distance (DMD) in mm, Strain of the diaphragm in %.
**Comparator(s)**	The comparators in this study are the conventional ultrasound methods used to assess diaphragm function in ALS, including diaphragm thickness, diaphragm thickening fraction (DTF), and diaphragm excursion.
**Reliability/Feasibility**	In healthy controls, the reliability was performed using the analysis of a kernel placed in the central layer of the diaphragm. The intra-rater reliability (ICC 1,1) was 0.985 and the inter-rater reliability (ICC 2,1) was 0.972, indicating very good reliability and reproducibility of the speckle tracking method.
**Physiological and/or technical outcomes**	In healthy controls:<br>Diaphragmatic excursion showed significant correlations with both DMD ($R = 0.76$, $p < 0.01$) and strain ($R = -0.61$, $p < 0.01$). Additionally, DMD and strain were significantly correlated with each other ($R = -0.67$, $p < 0.01$). However, DMD did not show any correlation with age, %FVC, phrenic CMAP amplitude, diaphragmatic thickness, or DTF.<br>In ALS patients:

	In patients with ALS, similar to healthy controls, the diaphragm's moving distance was greatest in the deep layer and least in the surface layer. DMD and strain were significantly correlated (R = -0.64, p < 0.01,). DMD was not associated with age (p = 0.71) or illness duration (p = 0.78). The central DMD during quiet breathing was 0.6 ± 1.4 mm, and diaphragm strain was -11.0 ± 6.2%, both significantly lower than in healthy controls (p < 0.01 and p = 0.04, respectively). In ALS patients, DMD positively correlated with phrenic CMAP amplitude (R = 0.63, p = 0.01) and negatively correlated with respiratory rate (R = -0.55, p = 0.02). Strain, however, did not significantly correlate with phrenic CMAP amplitude or respiratory rate. Paradoxical abdominal movements were noted in six ALS patients, and their respiratory rate was 17.7 ± 3.6 breaths/min, significantly higher than in healthy controls (p < 0.01). Notably, DMD decreased in some patients even when %FVC was within the normal range, and no significant correlation was found between DMD and diaphragm thickness or DTF.
**Clinical outcomes**	- DMD was significantly lower in ALS patients compared to healthy controls (0.6 ± 1.4 mm vs 2.2 ± 2.2 mm, p < 0.01).<br>- DMD was negatively correlated with the change in ALS Functional Rating Scale-Revised scores per month after the exam (*R* = -0.61, p = 0.02), and patients with a larger rate of decline had significantly lower DMD (p = 0.03).

**Author**	**Xin S, 2024**
**Country**	China
**Study design**	Prospective observational study, single-centre study
**Population**	- Type of population: Critically ill patients in ICU undergoing mechanical ventilation (for more than 48 hours)<br>- Number of participants: 89<br>- Age: Average age of 67.0 years (±12.7)<br>- Male/female: 65 males (73.0%), 24 females (26.9%)<br>- Detailed information about patients' disease: Respiratory system diseases: 63 cases (70.8%), Cardiovascular system diseases: 12 cases (13.5%), Nervous system diseases: 4 cases (4.5%), Endocrine system diseases: 4 cases (4.5%), Digestive system diseases: 6 cases (6.7%).
**New/advanced ultrasound approach and technical features**	**Tissue Doppler Imaging (TDI) applied to the diaphragm.**<br>• Timing of the assessment: The assessment was performed after a 30-minute spontaneous breathing trial (SBT).<br>• Position of participant: Participants were placed in a supine position.

	• Type of breathing effort: The breathing effort was spontaneous and quiet during the assessment.<br>• Hemidiaphragm assessed: Right side.<br>• Anatomical placement of the transducer/array: The phased array probe was positioned subcostally between the midclavicular line and anterior axillary line, directed cephalad towards the lower edge of the costal arch.<br>• Image acquisition procedure: The ultrasound instrument used was a PHILIPS HD15 (Amsterdam, the Netherlands), with a phased array probe operating at 2–4 MHz. The probe was positioned to obtain a top view of the diaphragm. To ensure precise TDI measurements, sampling lines were positioned perpendicular to the top of the diaphragm. A sampling frame width of 18.5–20.0 mm was selected to capture the full range of diaphragmatic motion, given its significant movement amplitude. A velocity scale of 5 cm/s was used. By reducing gain and filtering out high-frequency signals, a clear waveform of tissue movement velocity was achieved.<br>• Image analysis procedure: Image analysis was performed immediately after acquisition, focusing on measuring peak contraction velocity, peak relaxation velocity, contraction acceleration, and relaxation acceleration. The measurements were taken from the velocity waveform obtained during the assessment.<br>• Number of images analysed to retrieve results: Three consecutive respiratory cycles were observed, and an average value was calculated based on these three measurements.<br>• New Ultrasound markers: The following markers were retrieved in relation to this assessment: Peak contraction velocity (d-PCV) in cm/s, Peak relaxation velocity (d-PRV) in cm/s, Contraction acceleration (d-AC) in cm/s^2, Relaxation acceleration (d-AR) in cm/s^2, Mean Contraction Velocity (d-MCV) in cm/s
**Comparator(s)**	No comparator was used. Participants were divided into two groups: weaning success and weaning failure.
**Reliability/Feasibility**	N/a
**Physiological and/or technical outcomes**	N/a
**Clinical outcomes**	d-MCV did not show significant differences between the successful and failed weaning groups ($P > 0.05$). In contrast, d-PCV, d-PRV, d-AC, and d-AR acceleration were lower in the successful weaning group compared to the failed group. Additionally, the successful weaning group demonstrated significantly higher levels of diaphragmatic excursion (DE) than the failed weaning group ($P < 0.05$).

	d-PCV (diaphragmatic peak contraction velocity): AUC = 0.812 (95% CI: 0.718–0.906), cut-off value = 2.82 cm/s, sensitivity = 82.1%, specificity = 78.7%, positive predictive value = 89.3%, negative predictive value = 66.7%, positive likelihood ratio = 3.85, negative likelihood ratio = 0.23.<br>d-PRV (diaphragmatic peak relaxation velocity): AUC = 0.85 (95% CI: 0.773–0.933), cut-off value = 3.33 cm/s, sensitivity = 92.9%, specificity = 65.6%, positive predictive value = 84.8%, negative predictive value = 78.3%, positive likelihood ratio = 2.7, negative likelihood ratio = 0.11.<br>d-AC (diaphragmatic acceleration): AUC = 0.74 (95% CI: 0.630–0.859), cut-off value = 4.32 cm/s^2, sensitivity = 60.7%, specificity = 83.6%, positive predictive value = 88.1%, negative predictive value = 48.9%, positive likelihood ratio = 3.70, negative likelihood ratio = 0.47.<br>d-AR (diaphragmatic area change): AUC = 0.856 (95% CI: 0.781–0.936), cut-off value = 9.25 cm/s^2, sensitivity = 89.3%, specificity = 75.4%, positive predictive value = 88.5%, negative predictive value = 75.0%, positive likelihood ratio = 3.63, negative likelihood ratio = 0.14.<br>d-MCV (diaphragmatic mean contraction velocity): AUC = 0.567 (95% CI: 0.440–0.695), cut-off value = 1.56 cm/s, sensitivity = 75.1%, specificity = 47.5%, positive predictive value = 75.4%, negative predictive value = 46.4%, positive likelihood ratio = 1.43, negative likelihood ratio = 0.53.

**Author**	**Xu JH, 2021**
**Country**	China
**Study design**	Prospective observational study, single-centre study
**Population**	- Type of population: Stable COPD patients and healthy controls<br>- Number of participants: 77 (43 COPD patients, 34 healthy controls)<br>- Age: mean 64.5 (7.9) years in Stable COPD patient group and 63.8 years (7.0) in healthy controls group.<br>- Male/female: 39 (90.7-5) for Stable COPD patient group and 29 (85.3%) for healthy controls group.<br>- Detailed information about patients' disease: median mMRC score: 1 [1–2], median CAT score: 23 [18–26], median FEV1: 1.3 L [0.9–1.5], mean FEV1% predicted: 49.1% (SD = 17.2), mean FEV1/FVC: 51.8% (SD = 10.7), median FVC: 2.4 L [2.1–3.0].
**New/advanced ultrasound approach and technical features**	**Ultrasound shear wave elastography (SWE) to assess diaphragm stiffness.**<br><br>• Timing of the assessment: Not specifically mentioned.

	• Position of participant: Supine position with right arm positioned higher than the neck.<br>• Type of breathing effort: Not specifically mentioned, patient were instructed to maintain apnea after expiration.<br>• Hemidiaphragm assessed: Right.<br>• Anatomical placement of the transducer/array: The transducer was placed at the zone of apposition, specifically between the right anterior and midaxillary lines, at the 8th to 10th intercostal space.<br>• Image acquisition procedure: Logiq E9 ultrasound system with 9 MHz linear transducer, SWE mode with musculoskeletal preset. An 8 mm ultrasound gel pad was applied as a coupling agent, and minimal pressure was maintained to avoid compressing the diaphragm. SWE images were continuously acquired at a sampling rate of 2 Hz.<br>• Image analysis procedure: Offline analysis at least 14 days after capturing. Three homogeneous frozen images were selected, and four nonoverlapping circular regions of interest (ROIs) were drawn on the 2-D shear elasticity map to cover as much of the diaphragm as possible in each image.<br>• Number of images analysed to retrieve results: A total of three images were analysed for each participant to calculate the average shear wave velocity.<br>• Ultrasound markers: The primary ultrasound marker retrieved was the shear wave velocity (SWV), measured in meters per second (m/s).
**Comparator(s)**	No comparator
**Reliability/Feasibility**	The intra-observer ICC value for diaphragmatic shear wave velocity (SWV) at functional residual capacity (FRC) was 0.93 (95% CI 0.82, 0.98), indicating good to excellent reliability.
**Physiological and/or technical outcomes**	The diaphragmatic SWV at FRC was linked to:<br>- Forced expiratory volume in one second ($R = -0.30$, $p = .009$)<br>- Forced vital capacity ($R = -0.33$, $p = .003$)
**Clinical outcomes**	The diaphragmatic SWV at FRC was linked to:<br>- Modified Medical Research Council score ($R = 0.30$, $p = .001$)<br>- COPD assessment test score ($R = 0.48$, $p < .001$).<br>COPD group: Median SWV = 2.5 m/s (IQR 2.3–2.7 m/s) versus Control group: Median SWV = 2.1 m/s (IQR 1.8–2.5 m/s), $p = .008$.<br>The SWV for the control group was significantly lower than severe COPD patients ($p = 0.021$). However, no significant difference was observed between the control group and the mild–moderate COPD group ($p = 0.333$). The mild–moderate COPD did not differ from the severe COPD group ($p = 1.000$) in SWV

<table>
<tr><td>Author</td><td>Xu Q, 2022</td></tr>
<tr><td>Country</td><td>China</td></tr>
<tr><td>Study design</td><td>Prospective (study A) and retrospective (study B) observational study, multicentric study (2 centres)</td></tr>
<tr><td>Population</td><td>- Healthy subjects and critically ill mechanically ventilated patients
- Number of participants: Study A: 25 healthy subjects, 20 mechanically ventilated patients; Study B: 96 critically ill patients
- Healthy subjects (study A):
Age: 48.92 ± 16.29
Male: 14 (70%)
This predominantly healthy population had a low prevalence of comorbidities. Hypertension was present in 12% of individuals (3 patients), diabetes in 4% (1 patient), and chronic pulmonary disease in 8% (2 patients).
- Mechanically ventilated patients (study A):
Age: 57.40 ± 16.43
Male: 14 (70%)
This population had an APACHE II score of 15.0 ± 4.73. Comorbidities included hypertension (20%), chronic pulmonary disease (15%), diabetes (10%), chronic kidney disease (10%), malignant solid tumor (5%), and other conditions (10%). Etiologies of mechanical ventilation were sepsis (35%), ARDS (20%), postsurgical respiratory failure (15%), AECOPD (10%), trauma (5%), and others (15%).
- Critically ill patients – weaning success group, n = 59 (study B):
Age: median 64.00 years [54.50–74.00]
Male: 35 (59.3%).
Patients had an APACHE II score of 20.00 [18.00–25.00]. Comorbidities included hypertension (32.2%), diabetes (15.3%), chronic pulmonary disease (13.6%), chronic heart failure (11.9%), malignant solid tumor (11.9%), chronic kidney disease (10.2%), and other conditions (6.8%). The primary etiologies of mechanical ventilation were sepsis (30.5%), ARDS (15.3%), AECOPD (15.3%), postsurgical respiratory failure (13.6%), acute left heart failure (10.2%), trauma (3.4%), and other causes (13.6%). Outcomes included no failed SBT, no reintubation within 48 hours, no death within 7 days of extubation, NPPV in 8.5%, high-flow oxygen therapy in 16.9%, and a 28-day mortality of 13.6%.
- Critically ill patients – weaning failure group, n = 37 (study B):
Age: median 67.00 years [58.00–77.00]
Male: 25 (67.60%)</td></tr>
</table>

	• Patients had an APACHE II score of 18.00 [13.00–21.00]. Comorbidities included hypertension (32.4%), diabetes (16.2%), chronic pulmonary disease (13.5%), chronic heart failure (10.8%), malignant solid tumor (10.8%), chronic kidney disease (8.1%), and other conditions (5.4%). The primary etiologies of mechanical ventilation were sepsis (29.7%), ARDS (16.2%), AECOPD (16.2%), postsurgical respiratory failure (10.8%), acute left heart failure (10.8%), trauma (2.7%), and other causes (10.8%). Outcomes included a failed SBT in 81.1%, reintubation within 48 hours in 10.8%, and 28-day mortality of 43.2%.
**New/advanced ultrasound approach and technical features**	**Two-dimensional (2D) speckle tracking to measure diaphragm longitudinal strain (DLS)**<br><br>• Timing of the assessment: 30 min after a spontaneous breathing test (SBT) or before reventilation.<br>• Position of participant: Participants were positioned in a semirecumbent position at 45 degrees.<br>• Type of breathing effort: The breathing effort assessed included both quiet breathing and deep breathing.<br>• Hemidiaphragm assessed: Right side.<br>• Anatomical placement of the transducer/array: The transducer was placed in the zone of apposition of the diaphragm.<br>• Image acquisition procedure: Images were acquired using a commercial ultrasound machine (Vivid 7 and Vivid S6, GE, USA) with a linear array transducer (4.0–13.0 MHz for Vivid S6 and 4.0–11.0 MHz for Vivid 7). More than 6 seconds of video was collected.<br>• Image analysis procedure: The analysis was performed offline using EchoPacs software. The analysis involved selecting the first respiratory cycle and using a speckle tracking filter to obtain strain curves and maximum strain values. The region of interest (ROI) was adjusted multiple times to ensure accurate tracking. Three segments of the diaphragmatic tracking region of interest (ROI) were verified. By adjusting the yellow and green lines on the left (which typically correspond to the closure of the aortic valve), the starting point of the analysis cycle can be altered to evaluate the maximum longitudinal strain difference between the lowest and highest points of the curve.<br>• Number of images analysed to retrieve results: Overall not explicitly mentioned. For study A, it's mentioned that 3 ultrasound cycleloops were acquired for each measurement, for a total of 747 ultrasound cineloops.

	• New Ultrasound markers: The study retrieved maximum longitudinal strain values (DLS) as the primary marker, measured in percentage (%). The more negative value means the higher degree of deformation (contraction)
**Comparator(s)**	1. Conventional diaphragmatic ultrasound parameters: diaphragmatic excursion (DE), diaphragmatic thickness at end-inspiration (DTei), end-expiration (DTee), and diaphragmatic thickening fraction (DTF)<br>2. Rapid shallow breathing index (RSBI)<br>3. Participants were divided in weaning success and weaning failures groups.
**Reliability/Feasibility**	In healthy volunteers at rest, intraoperator reliability was good for both operators, with ICC values of 0.86 (95% CI: 0.63–0.95) for the first operator and 0.87 (95% CI: 0.74–0.94) for the second. Interoperator reliability was also good, with an ICC of 0.87 (95% CI: 0.73–0.94). During deep breathing, intraoperator reliability remained good, with ICCs of 0.84 (95% CI: 0.66–0.92) for the first operator and 0.80 (95% CI: 0.59–0.90) for the second, while interoperator reliability was good at 0.78 (95% CI: 0.57–0.90).<br>In mechanically ventilated patients, intraoperator reliability was excellent, with ICCs of 0.95 (95% CI: 0.87–0.98) for the first operator and 0.92 (95% CI: 0.82–0.97) for the second. Interoperator reliability was also excellent, with an ICC of 0.94 (95% CI: 0.85–0.97).
**Physiological and/or technical outcomes**	DLS demonstrated a fair linear relationship with DTF (Pearson R^2 = 0.73, p < 0.0001) and DE (Pearson R^2 = 0.61, p < 0.0001), but a weak relationship with diaphragmatic thickness (DTee and DTei, R^2 = 0.01, p = 0.3336; R^2 = 0.07, p = 0.0071, respectively).
**Clinical outcomes**	The DLS (%) values in the diaphragmatic zone of apposition were measured in healthy subjects during eupnoea and deep breathing, as well as in mechanically ventilated patients, yielding values of −15 (12.75 to 17.25), −42.0 (−35.0 to 50.25), and −16 (−10.5 to 34.0), respectively. No significant differences in sex were observed between healthy subjects and mechanically ventilated patients (P > 0.05).<br>Among 269 patients with diaphragmatic ultrasound records, 96 participated in Study B.<br>Significant differences were noted between the success and failure groups for DLS (%) (success: –25.00 [−34 to −16], failure: –13.00 [−18.00 to −7.00]; p < 0.001), DTF (%) (success: 25.40 [21.18 to 32.84], failure: 20.82 [17.05 to 25.86]; p < 0.001), and DE (mm) (success: 14.50 [11.55 to 19.50], failure: 10.80 [8.20 to 13.30]; p < 0.001). However, DTee (mm) and DTei (mm) showed no significant differences between groups (DTee: success 2.80 [2.20 to 3.40], failure 2.70 [2.40 to 3.20]; p = 0.827; DTei: success 3.60 [2.80 to 4.10], failure 3.30 [2.80 to 3.90]; p = 0.564).

	The area under the curve (AUC) values for DLS, RBSI, DTF, and DE in predicting successful weaning were 0.794, 0.794, 0.723, and 0.728, respectively. The optimal cut-off values for predicting weaning success were identified as follows: DLS < -21% (sensitivity 89.19%, specificity 64.41%), DTF > 83 (sensitivity 59.46%, specificity 88.14%), DE > 11.2 (sensitivity 56.76%, specificity 79.66%). Overall, DLS exhibited a high predictive value for successful mechanical weaning. However, DLS offers no advantage over RSBI

**Author**	Ye X, 2013
**Country**	China
**Study design**	Observational study (cross-sectional), single-centre study
**Population**	- Healthy volunteers<br>- Number of participants: 21<br>- Age: 43±11 years<br>- Male/Female: 12/9<br>- No specific diseases: Participants had no history of diaphragm dysfunction, chronic obstructive pulmonary diseases, asthma, thoracic surgery, pleural thickening, or smoking.
**New/advanced ultrasound approach and technical features**	**2D strain ultrasound speckle tracking, which quantifies segmental longitudinal deformation (strain) of the right diaphragm.**<br><br>• Timing of Assessment: Not applicable<br>• Position of participant: The patients were positioned in a supine position.<br>• Type of Breathing Effort: The assessment included both quiet breathing and forced breathing efforts.<br>• Hemidiaphragm Assessed: Right<br>• Anatomical Placement of Transducer: The transducer was placed just below the right costal margin around the midclavicular line, using the right intrahepatic vein branch as an anatomical landmark.<br>• Image Acquisition Procedure: The image acquisition was conducted using a GE Healthcare Vivid E9 ultrasound system with an M5S convex transducer, capturing videos of diaphragm motions (M-Mode) over four continuous cardiac cycles, with an appropriate total gain and depth set. The system operated at 50-70 frames per second.<br>• Image Analysis Procedure: This procedure was conducted offline after the image acquisition, where the recorded videos were analysed to measure diaphragm strain, with Q-analysis software.

	The peritoneum, mid-diaphragm, and pleural border were aligned with the epicardial, midmyocardial, and endocardial lines as defined by the cardiac motion analysis software. After pausing the cursor, the software automatically detected the diaphragm's longitudinal deformation. The value obtained during the maximal inspiratory slope represented the diaphragm's deformation in the inspiratory phase. Tracking occurred from the R wave to aortic valve closure (AVC) within the same cardiac cycle. The right diaphragm's hyper-echogenic curved line was typically divided into three segments: the dome position (highest point), the zone of apposition (cylindrical region against the lower rib cage) in the right side, and the crural region in the left side. A negative value indicated active systole of the diaphragm segment, while a positive value indicated passive stretching.<br>• Number of Images Analysed: A total of four continuous cardiac cycles were analysed to derive the results.<br>• Ultrasound Markers: The primary ultrasound marker retrieved was longitudinal strain, measured in percentage (%), which provided insights into diaphragm anatomy and function.
**Comparator(s)**	In this article, the 2D strain ultrasound speckle tracking technique was not compared to any other methods.
**Reliability/Feasibility**	N/a
**Physiological and/or technical outcomes**	- The 2D strain ultrasound speckle tracking technique was able to detect differences in the deformation patterns of different regions of the diaphragm. The crura of the diaphragm showed strain values of -5.24±3.00% during quiet breathing and -7.42±5.10% during forced breathing (p-value 0.0709). The dome of the diaphragm had strain values of 3.24±1.64% for quiet breathing and 4.10±2.34% for forced breathing (p-value 0.2780). The zone of apposition exhibited strain values of -6.24±2.91% during quiet breathing and -10.00±4.58% during forced breathing, with a significant p-value of 0.0051. Overall, the whole diaphragm displayed strain values of -2.14±1.80% for quiet breathing and -4.62±2.56% for forced breathing, with a significant p-value of 0.0002.<br>- During quiet breathing, no significant difference was observed in the strains of the crura of the right diaphragm and the zone of apposition (P=0.198). However, a significant difference was noted during forced breathing (P=0.024).
**Clinical outcomes**	N/a

**Author**	**Zhang J, 2023**
**Country**	China

**Study design**	Prospective observational study, single-centre study
**Population**	- Type of population: Patients with acute exacerbation of chronic obstructive pulmonary disease (AECOPD)<br>- Number of participants: 112<br>- Age: Group C had a mean age of 72.2 ± 10.4 years, Group D had a mean age of 70.7 ± 11.2 years.<br>- Male/female: 87 males/ 25 females. Group C had 49 males (84%), Group D had with 38 males (70%).<br>- The patients were classified into groups A, B, C, and D based on the GOLD guideline, using CAT scores, mMRC scores, and the number of acute exacerbations within a year: A (low risk, few symptoms), B (low risk, many symptoms), C (high risk, few symptoms), and D (high risk, many symptoms). Among 112 COPD patients with ≥2 acute exacerbations within a year, divided into group C (n = 58) and group D (n = 54).<br>Group C: FEV1 of 1158.0 mL (825.0, 1988.5), FVC of 1985.0 mL (1352.1, 2478.5), and an FEV1/FVC ratio of 45.9 ± 11.5%.<br>Group D: FEV1 of 814.9 mL (667.7, 1739.8), FVC of 1749.3 mL (1276.0, 2280.0), and an FEV1/FVC ratio of 38.8 ± 10.8%.
**New/advanced ultrasound approach and technical features**	**Ultrasound shear wave elastography (SWE) to assess diaphragm stiffness.**<br><br>• Timing of the assessment: The assessments were performed during the hospitalization of AECOPD patients, specifically after their admission to the respiratory department.<br>• Position of participant: Supine. For diaphragmatic SWE measurement, the patient was supine with the right arm above the neck.<br>• Type of breathing effort: Deep breathing (*"Diaphragmatic SWE maps were acquired at total lung capacity and functional residual capacity")*<br>• Hemidiaphragm assessed: Right.<br>• Anatomical placement of the transducer/array: The transducer was placed at the right 8th to 10th intercostal and anterior axillary areas, spanning two rib spaces to avoid compression.<br>• Image acquisition procedure: A GE S8 ultrasonic system and a 9 L linear array transducer (9 MHz) were used to measure DTF and diaphragm SWE. After clearly visualizing the diaphragm, the SWE sampling frame was placed on the diaphragm, and diaphragmatic SWE maps were acquired at total lung capacity and functional residual capacity.<br>• Image analysis procedure: Image analysis was performed immediately after acquisition, with the diaphragm being visualised at both total lung capacity and functional residual capacity. Six circular regions of interest were overlaid on

	the SWE maps to obtain the elastic modulus of shear waves, which were averaged to calculate the diaphragmatic shear wave elasticity.<br>• Number of images analysed to retrieve results: Multiple images were acquired, although the exact number analysed for results was not specified in the provided text.<br>• The ultrasound markers retrieved included diaphragm shear wave elasticity at functional residual capacity (DsweFRC) in kPa and diaphragm stiffening rate (DSR), calculated as DSR = (diaphragmatic shear wave elasticity of total lung capacity -DsweFRC)/ DsweFRC.
**Comparator(s)**	1. Diaphragm thickening fraction (DTF) measured by ultrasound<br>2. Pulmonary function tests (FEV1, FVC, FEV1/FVC)<br>3. Clinical data such as CAT score and mMRC score
**Reliability/Feasibility**	The internal reliability of the measurements taken by the same examiner at different times was found to be good (ICC = 0.789, p = 0.000, 95% CI 0.584–0.915). Similarly, the external reliability of the variables assessed by different examiners was also good (ICC = 0.727, p = 0.001, 95% CI 0.355–0.900)
**Physiological and/or technical outcomes**	In the "high risk, few symptoms" group, DsweFRC showed moderate negative correlations with TFdi and FEV1/FVC (R = −0.408 to −0.492, p < 0.05) and weak negative correlations with FEV1 and FVC (R = −0.293 to −0.373, p < 0.05). DSR (% change) also exhibited moderate negative correlations with TFdi and FEV1/FVC (R = −0.429 to −0.430, p < 0.05) and weak negative correlations with FEV1 and FVC (R = −0.308, p < 0.05).<br>In the "high risk, many symptoms" group, DsweFRC demonstrated strong negative correlations with TFdi (R = −0.697, p < 0.000), a moderate negative correlation with FEV1/FVC (R = −0.538, p < 0.000), and weak negative correlations with FEV1 and FVC (R = −0.306 to −0.373, p < 0.05). DSR (% change) also showed strong negative correlations with TFdi and FEV1/FVC (R = −0.623 to −0.697, p = 0.000) and weak negative correlations with FEV1 and FVC (R = −0.304 to −0.386, p < 0.05).<br>Both DsweFRC and DSR in both groups presented weak positive correlations with $PaCO_2$ (R = 0.308 to 0.328, p < 0.05). They had no correlation with PaO_2 (all p > 0.05).
**Clinical outcomes**	DsweFRC (median 20.58, IQR: 18.08-39.0) and DSR (mean 0.50 ± 0.14) were significantly higher in the "high risk, many symptoms" group compared to the "high risk, few symptoms" group (DsweFRC median 18.52, IQR: 12.2-28.5; DSR mean 0.41 ± 0.17, p = 0.040 and p = 0.004, respectively). In the "high risk, few symptoms" group, DsweFRC showed weak positive correlations with the CAT (R = 0.306) and mMRC scores (R = 0.274). DSR also demonstrated weak positive correlations with the CAT score and mMRC score (R = 0.303-0.398). In the "high risk, many symptoms" group, both DsweFRC and DSR exhibited weak positive

	correlations with the CAT score (R = 0.302-0.395) and mMRC score (R = 0.349, only for DsweFRC). DSR showed a moderate correlation with the mMRC score (R = 0.462).<br>DsweFRC and DSR tended to increase as the number of acute exacerbations within 1 year increased in both groups.

**Author**	**Zhang T, 2024**
**Country**	China
**Study design**	Prospective observational study, single-centre study
**Population**	- Type of population: Healthy subjects with normal spirometry<br>- Number of participants: 212<br>- Age: 43.6 ± 14.4 years<br>- Male/female: 105 males (49.53%), 107 females<br>- BMI of 22.8 ± 2.6 kg/m². Smoking exposure was reported in 9.9% of individuals (n = 21). Regarding activity levels, 31.6% (n = 67) had a sedentary work, while 41.0% (n = 87) were physically active.
**New/advanced ultrasound approach and technical features**	**Ultrasound shear wave elastography (SWE) to assess diaphragm stiffness.**<br><br>• Timing of the assessment: Not applicable – healthy subjects.<br>• Position of participant: Participants were positioned in a supine position during the ultrasound assessments.<br>• Type of breathing effort: Participants were instructed to breath calmly and smoothly.<br>• Hemidiaphragm assessed: Right side.<br>• Anatomical placement of the transducer/array: The transducer was placed in the 8–10 intercostal space of the right midaxillary line, utilizing the liver as an acoustic window.<br>• Image acquisition procedure: The imaging was performed using a 3–11 MHz linear array probe and a Resona 8 ultrasound system (Mindray Medical International, China), with specific attention to obtaining clear two-dimensional images of the diaphragm. The diaphragm was positioned centrally within the sampling frame. After the ruler was five stars, a uniform light blue color was selected in the frame, and a dynamic picture was stored.<br>• Image analysis procedure: The dynamic image was analysed retrospectively to identify the most uniform and consistent color frame of the elastogram within the sampling frame. Measurements were taken for shear modulus (SM) and dispersion (SD) of the diaphragm. The size of the sampling frame for the

	region of interest was adjusted based on the thickness of each individual's diaphragm, ensuring that the entire diaphragm layer was included in the region of interest.<br>• Number of images analysed to retrieve results: A total of three images were analysed.<br>• New Ultrasound markers: Shear modulus (Mean, Max, Min, SD) in kPa.
**Comparator(s)**	No comparator. Even though the authors performed some other conditional diaphragm ultrasound assessments, they did not analyze any relationship with shear wave elastography (SWE).
**Reliability/Feasibility**	N/a
**Physiological and/or technical outcomes**	The study population exhibited the following results for SMdi descriptors: 16.72 ± 4.07 kPa for SMdi mean value descriptor, 25.04 ± 5.58 kPa for SMdi maximum value descriptor, 11.06 ± 3.88 kPa for SMdi minimum value descriptor , and a 2.56 ± 0.98 kPa for SMdi standard deviation (SD) value descriptor.
**Clinical outcomes**	For the age groups, the young group had a SMdi mean values of 17.22 ± 4.06 kPa, the adult group had 16.06 ± 3.87 kPa, and the elderly group had 16.86 ± 4.45 kPa ($p = 0.159$). The SMdi maximum values were 25.73 ± 5.51 kPa for the young group, 24.46 ± 5.61 kPa for the adult group, and 24.46 ± 5.65 kPa for the elderly group ($p = 0.258$). The SMdi minimum values were 11.16 ± 3.74 kPa for the young group, 10.61 ± 3.71 kPa for the adult group, and 11.91 ± 4.60 kPa for the elderly group ($p = 0.253$). The SMdi SD values for the age groups were 2.70 ± 0.92 kPa for the young group, 2.51 ± 1.07 kPa for the adult group, and 2.98 ± 0.90 kPa for the elderly group ($p = 0.107$).<br>In terms of body mass index (BMI) groups, the mean SMdi values for the low weight group were 16.01 ± 3.10 kPa, for the normal weight group it was 17.12 ± 3.98 kPa, for the overweight group it was 16.19 ± 3.68 kPa, and for the obesity group it was 16.82 ± 4.91 kPa ($p = 0.506$). The SMdi maximum values were 24.69 ± 3.96 kPa for the low weight group, 25.06 ± 5.39 kPa for the normal weight group, 24.56 ± 5.13 kPa for the overweight group, and 25.69 ± 6.90 kPa for the obesity group ($p = 0.773$). The SMdi minimum values were 10.09 ± 4.08 kPa for the low weight group, 11.60 ± 3.81 kPa for the normal weight group, 10.83 ± 3.70 kPa for the overweight group, and 10.64 ± 4.13 kPa for the obesity group ($p = 0.307$). The SMdi SD values for the BMI groups were 2.71 ± 0.92 kPa for the low weight group, 2.51 ± 1.00 kPa for the normal weight group, 2.48 ± 0.88 kPa for the overweight group, and 2.73 ± 1.09 kPa for the obesity group ($p = 0.496$).<br>Lastly, for lifestyle groups, the mean SMdi values were 17.29 ± 4.17 kPa for the physical group, 16.27 ± 4.38 kPa for the general group, and 16.37 ± 3.45 kPa for the sedentary group ($p = 0.226$). The SMdi maximum values were 25.66 ± 5.91 kPa for the physical group, 24.78 ± 5.92 kPa for the general group, and 24.40 ± 4.56 kPa for the sedentary group. The SMdi minimum values were 11.31 ± 3.76

	kPa for the physical group, 11.36 ± 3.85 kPa for the general group, and 10.48 ± 4.04 kPa for the sedentary group. The SMdi SD values were 2.61 ± 1.01 kPa for the physical group, 2.41 ± 0.84 kPa for the general group, and 2.64 ± 1.06 kPa for the sedentary group (p = 0.359).
