# Supplementary material for: Ultrasound innovations in diaphragm assessment: an integrative review of expanding clinical applications
Source: Eur Respir Rev. 2025 Oct 8;34(178):250089. doi: 10.1183/16000617.0089-2025 (PMC12505151; doi:10.1183/16000617.0089-2025)
Supplement: Supplementary file 3 [file ERR-0089-2025.SUPPLEMENT3.pdf]

Supplementary information 3
# - Illustration of Ultrasound Techniques

- **The “Area Method”**

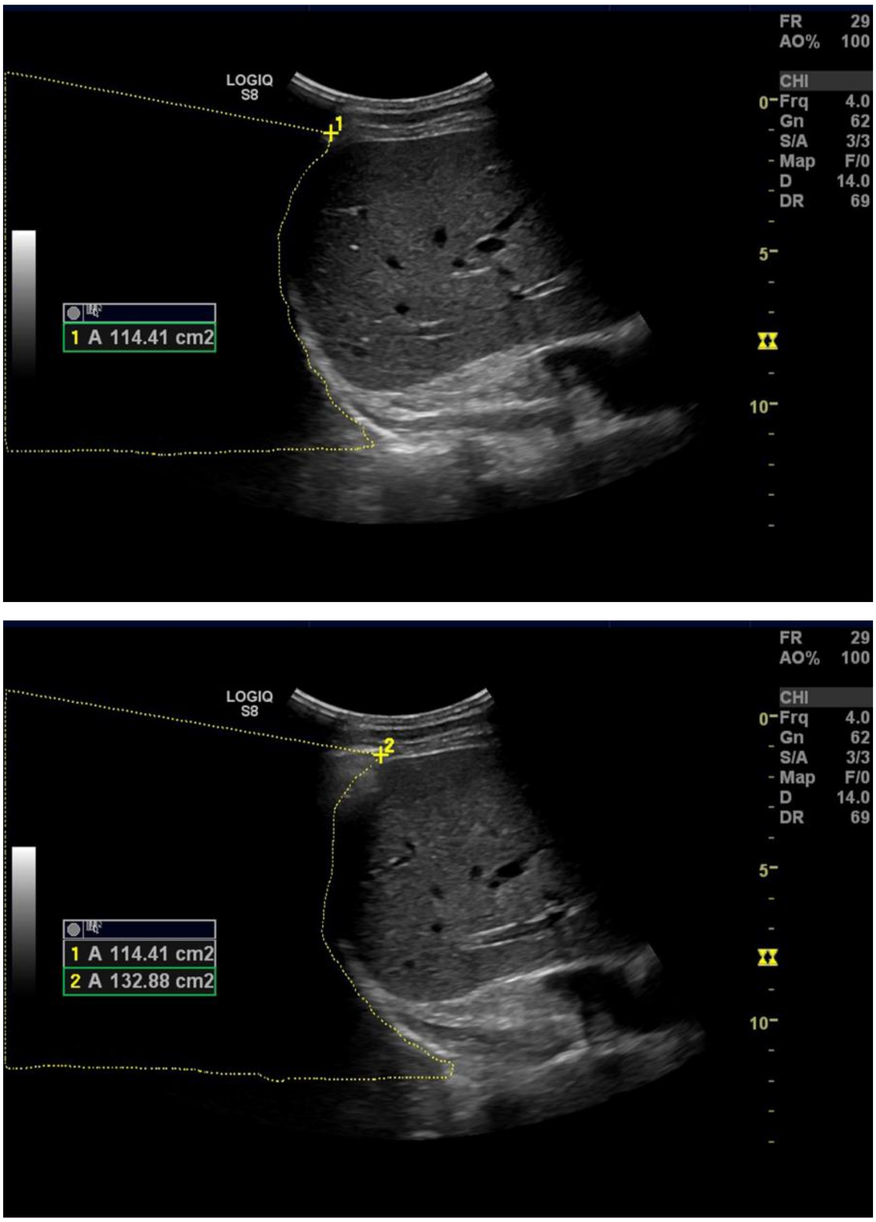

a

b

Figure E1 - An example of the area method for the assessment of diaphragm movement: (a) Full expiration, indicating the minimum area over the diaphragm; (b) Full inspiration, showing the maximum area over the diaphragm. Reproduced from *Skaarup SH., et al., 2018* (1), under the terms of the Creative Commons CC-BY license.

- **Contrast-Enhanced Ultrasound (CEUS)**

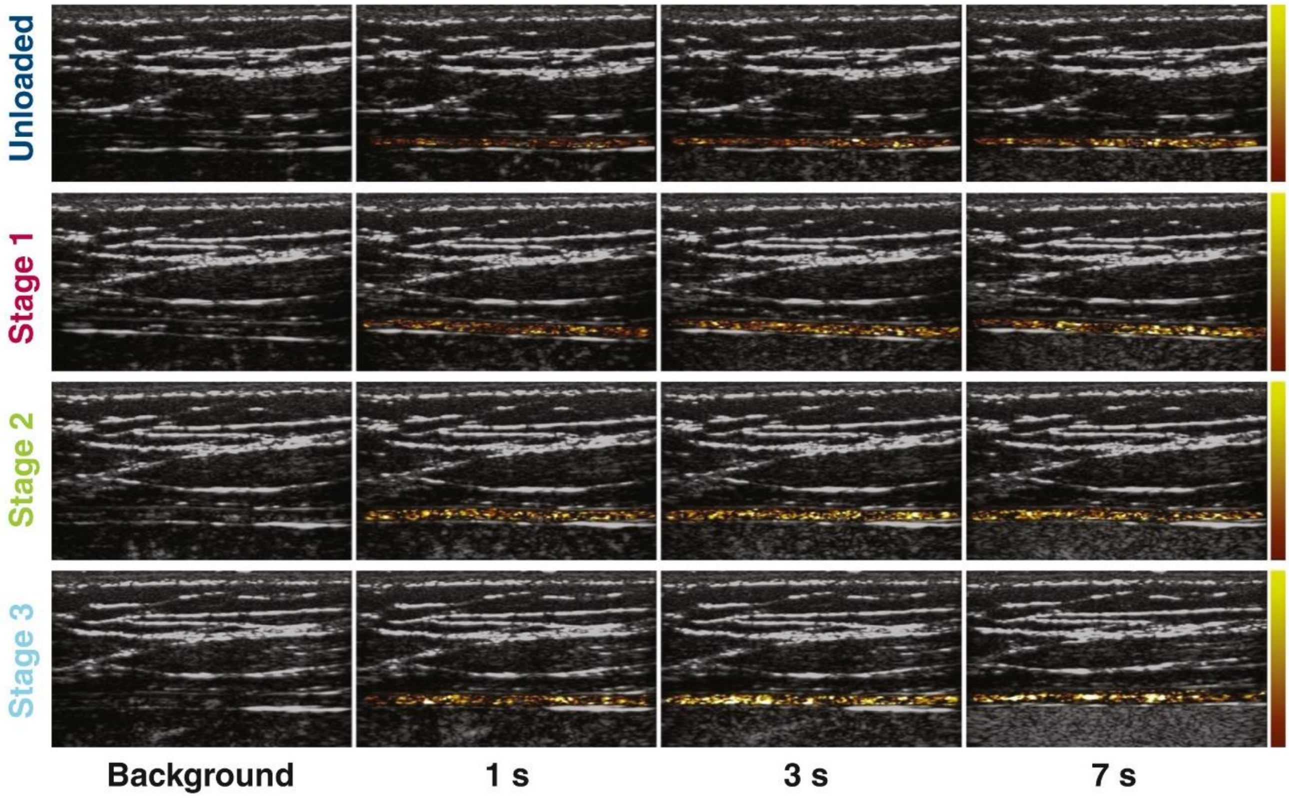

Figure E2 - Example of contrast-enhanced ultrasound measurements of blood flow in the diaphragm and liver, visualized at the costal region with a linear ultrasound probe. Background-subtracted, color-coded contrast-enhanced ultrasound images of a male participant at various levels of inspiratory pressure threshold loading during a single experimental session. Reproduced with permission from *Bird JD., et al., 2024* (2).

- **Echogenicity/Echodensity (ED)**

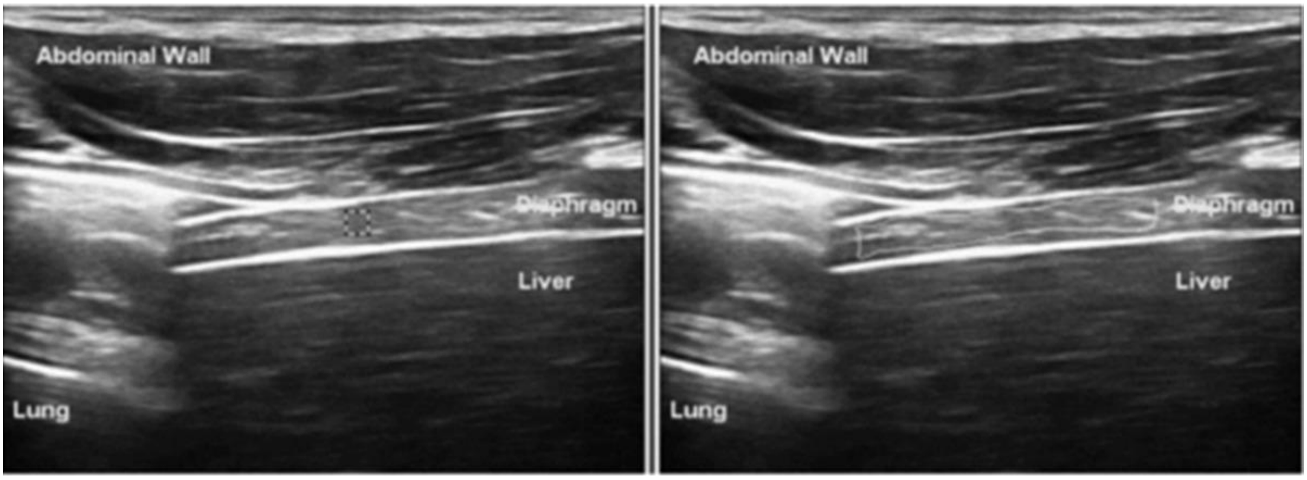

Figure E3 - Echogenicity analysis of the diaphragm muscle is presented as follows: In the left image, a predefined square area of 20 × 20 pixels was utilized (square technique), while in the right image, the anatomical boundary of the muscle was outlined (trace technique). Reproduced with permission from *Sarwal A., et al., 2015* (3).

- **Excursion of the Zone of Apposition (EXdi-ZOA)**

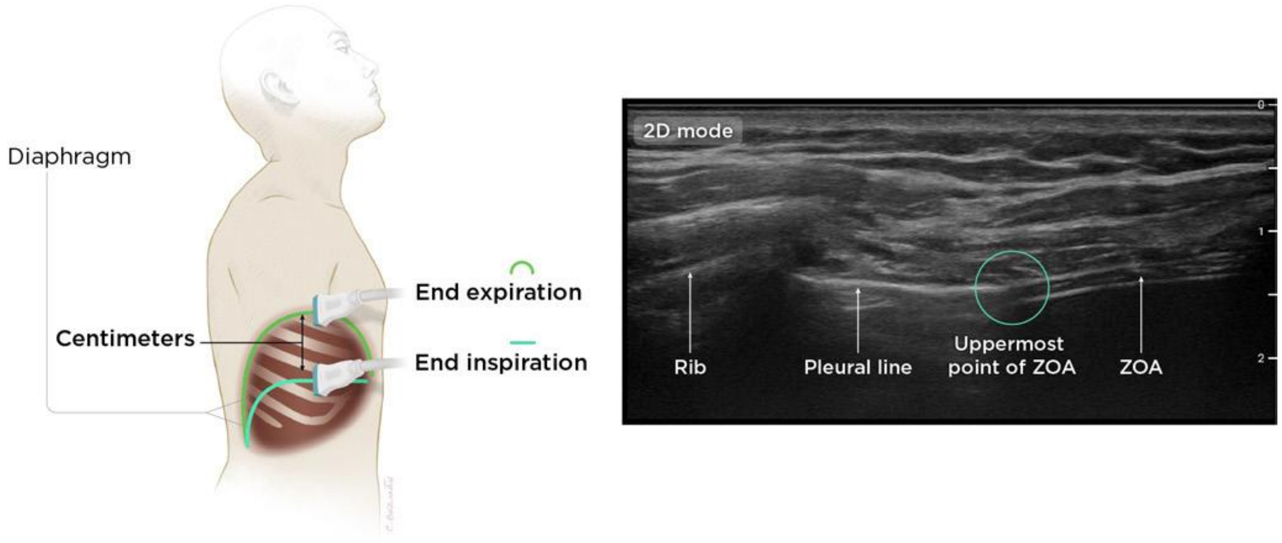

Figure E4 - Illustration of diaphragm excursion assessment, using the uppermost point of the zone of apposition (ZOA) as a reference, measured with a linear probe placed along the mid-axillary line. Reproduced with permission from *Da Conceição D., et al., 2024* (4).

- **Shear Wave Elastography (SWE)**

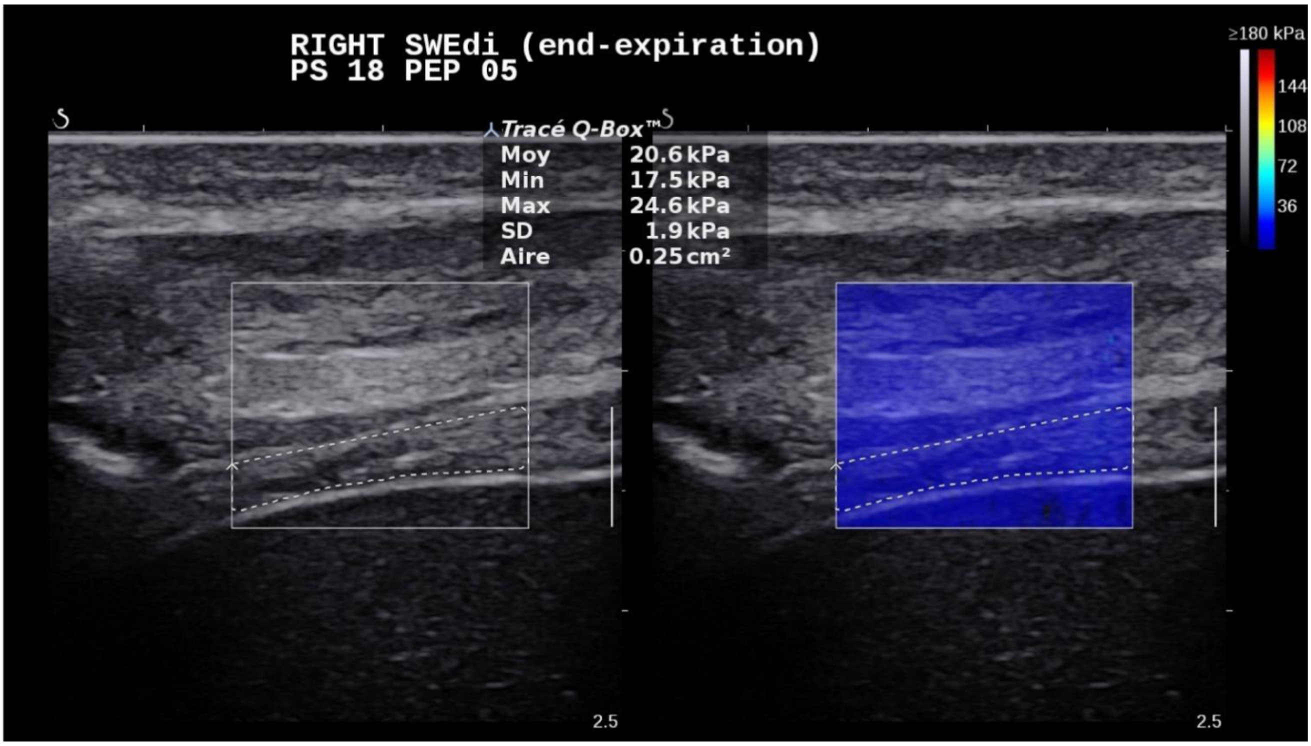

Figure E5 - An example of the image acquisition at the zone of apposition using Shear Wave elastography to assess the right diaphragm at end-expiration in a patient with Immune-Mediated Necrotizing Myopathy, receiving mechanical ventilation with pressure support mode. A region of interest is then traced inside the elastogram map. Reproduced from *Neto Silva I., et al., 2024* (5), under the terms of the Creative Commons CC-BY license.

- **Speckle Tracking (ST)**

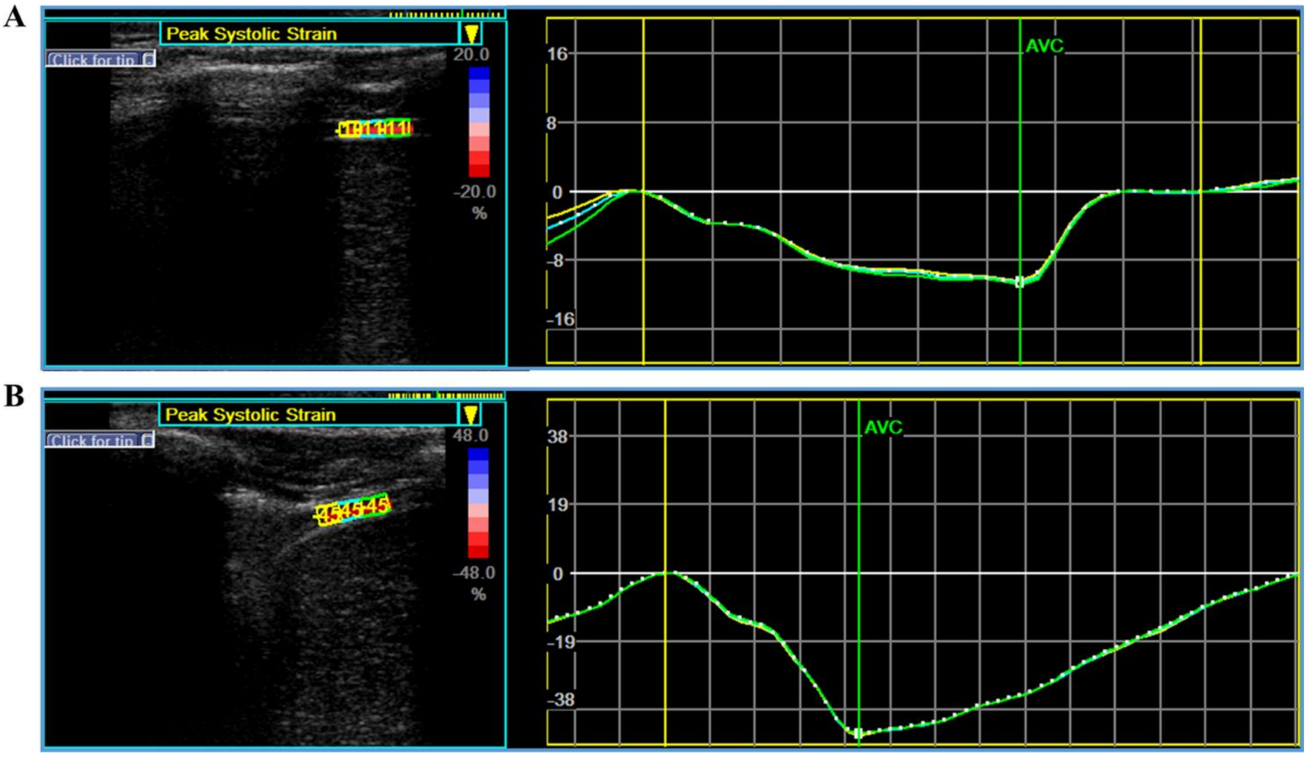

Figure E6 – Region of interest tracked by the software during diaphragmatic longitudinal strain quantification via speckle tracking in eupnoea (A) and deep breathing (B). The curves depict diaphragm strain over time, with more negative values indicating greater deformation (contraction). Reproduced from *Xu Q., et al., 2022* (6), under the terms of the Creative Commons CC-BY license.

- **Pulsed-Wave Tissue Doppler Imaging (PW-TDI)**

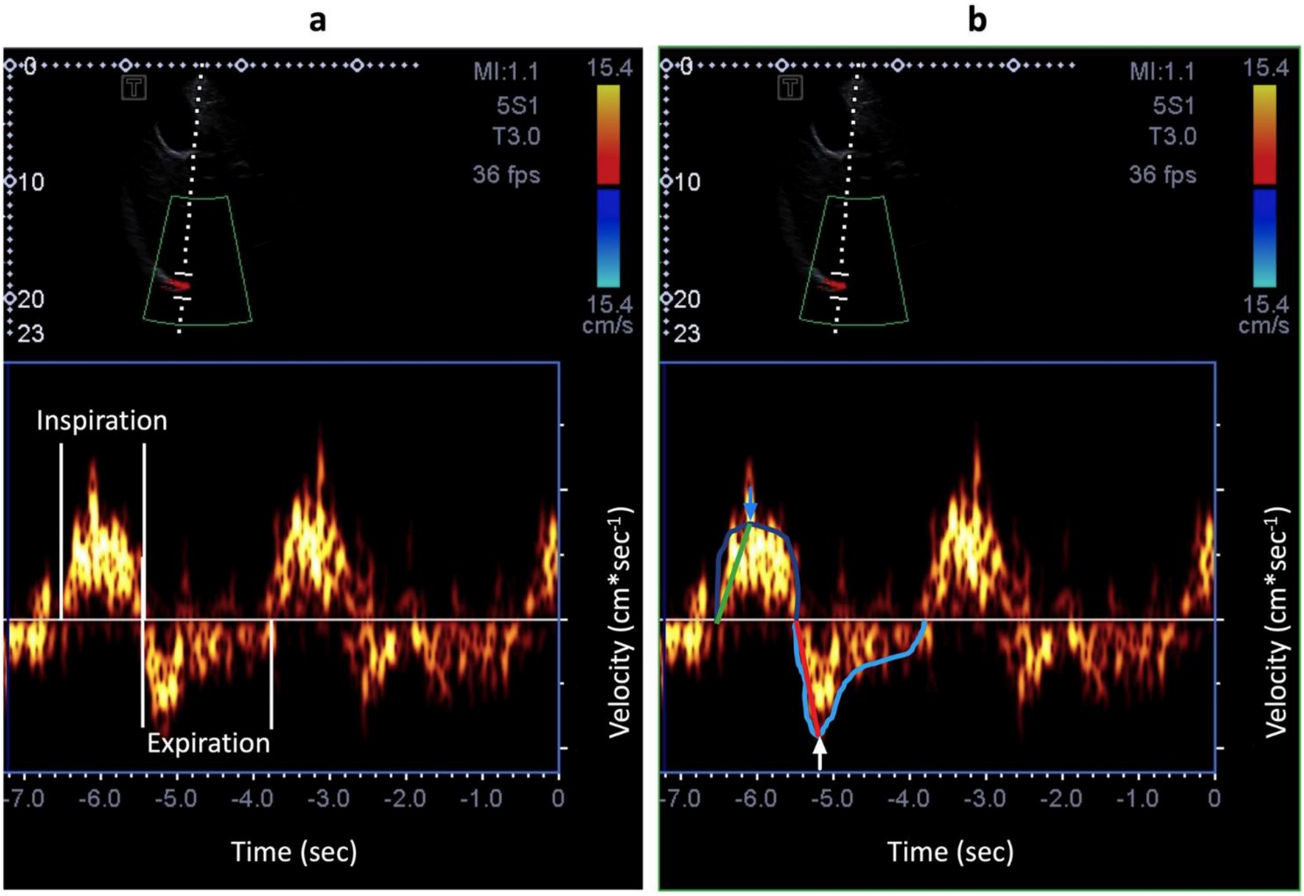

Figure E7 – Illustration of diaphragm assessment using Tissue Doppler Imaging. (a) Presents the range of inspiratory and expiratory diaphragmatic displacement velocity over time, while (b) illustrates diaphragmatic excursion parameters assessed via tissue Doppler imaging: dark blue line, Mean Contraction Velocity (MCVdi); light blue arrow, Peak Contraction Velocity (PCVdi); area under inspiratory velocity curve, Velocity-time Integral (VTIdi) - inspiratory displacement; green line, Inspiratory Acceleration; light blue line, Mean Expiratory Velocity; white arrow, Peak Relaxation Velocity (PRVdi); area under expiratory velocity curve, expiratory displacement; red line, Maximal Relaxation Rate (MRRdi). Reproduced with permission from *Cammarota G., et al., 2020* (7).

References

1. Skaarup SH, Lokke A, Laursen CB. The Area method: a new method for ultrasound assessment of diaphragmatic movement. Crit Ultrasound J. 2018;10(1):15.
2. Bird JD, Lance ML, Banser TRW, Thrall SF, Cotton PD, Lindner JR, et al. Quantifying Diaphragm Blood Flow With Contrast-Enhanced Ultrasound in Humans. Chest. 2024;166(4):821-34.
3. Sarwal A, Parry SM, Berry MJ, Hsu FC, Lewis MT, Justus NW, et al. Interobserver Reliability of Quantitative Muscle Sonographic Analysis in the Critically Ill Population. J Ultrasound Med. 2015;34(7):1191-200.
4. Da Conceicao D, Perlas A, Giron Arango L, Wild K, Li Q, Huszti E, et al. Validation of a novel point-of-care ultrasound method to assess diaphragmatic excursion. Reg Anesth Pain Med. 2024;49(11):800-4.
5. Neto Silva I, Kharat A, Marzano., Marchi E, Duarte JA, Bendjelid K. Unsuccessful Weaning From Mechanical Ventilation in a Patient With An Immune-Mediated Necrotizing Myopathy. A Case Report That Demonstrates the Usefulness of Shear-Wave Elastography. Chest. 2024;165(6):e45-e7.
6. Xu Q, Yang X, Qian Y, Hu C, Lu W, Cai S, et al. Comparison of assessment of diaphragm function using speckle tracking between patients with successful and failed weaning: a multicentre, observational, pilot study. BMC Pulm Med. 2022;22(1):459.
7. Cammarota G, Boniolo E, Tarquini R, Vaschetto R. Diaphragmatic excursion tissue Doppler sonographic assessment. Intensive Care Med. 2020;46(9):1759-60.
